# Simple nutrients bypass the requirement for HLH-30 in coupling lysosomal nutrient sensing to survival

**DOI:** 10.1371/journal.pbio.3000245

**Published:** 2019-05-14

**Authors:** John T. Murphy, Haiyan Liu, Xiucui Ma, Alex Shaver, Brian M. Egan, Clara Oh, Alexander Boyko, Travis Mazer, Samuel Ang, Rohan Khopkar, Ali Javaheri, Sandeep Kumar, Xuntian Jiang, Daniel Ory, Kartik Mani, Scot J. Matkovich, Kerry Kornfeld, Abhinav Diwan

**Affiliations:** 1 Center for Cardiovascular Research and Division of Cardiology, Department of Internal Medicine, Washington University School of Medicine, St. Louis, Missouri, United States of America; 2 John Cochran VA Medical Center, St. Louis, Missouri, United States of America; 3 Department of Developmental Biology, Washington University School of Medicine, St. Louis, Missouri, United States of America; 4 Department of Cell Biology and Physiology, Washington University School of Medicine, St. Louis, Missouri, United States of America; University of Massachusetts Medical School, UNITED STATES

## Abstract

Lysosomes are ubiquitous acidified organelles that degrade intracellular and extracellular material trafficked via multiple pathways. Lysosomes also sense cellular nutrient levels to regulate target of rapamycin (TOR) kinase, a signaling enzyme that drives growth and suppresses activity of the MiT/TFE family of transcription factors that control biogenesis of lysosomes. In this study, we subjected worms lacking basic helix–loop–helix transcription factor 30 (*hlh-30*), the *Caenorhabditis elegans* MiT/TFE ortholog, to starvation followed by refeeding to understand how this pathway regulates survival with variable nutrient supply. Loss of HLH-30 markedly impaired survival in starved larval worms and recovery upon refeeding bacteria. Remarkably, provision of simple nutrients in a completely defined medium (*C*. *elegans* maintenance medium [CeMM]), specifically glucose and linoleic acid, restored lysosomal acidification, TOR activation, and survival with refeeding despite the absence of HLH-30. Worms deficient in lysosomal lipase 2 (*lipl-2*), a lysosomal enzyme that is transcriptionally up-regulated in starvation in an HLH-30–dependent manner, also demonstrated increased mortality with starvation–refeeding that was partially rescued with glucose, suggesting a critical role for LIPL-2 in lipid metabolism under starvation. CeMM induced transcription of vacuolar proton pump subunits in *hlh-30* mutant worms, and knockdown of vacuolar H^+^-ATPase 12 (*vha-12*) and its upstream regulator, nuclear hormone receptor 31 (*nhr-31*), abolished the rescue with CeMM. Loss of Ras-related GTP binding protein C homolog 1 RAGC-1, the ortholog for mammalian RagC/D GTPases, conferred starvation–refeeding lethality, and RAGC-1 overexpression was sufficient to rescue starved *hlh-30* mutant worms, demonstrating a critical need for TOR activation with refeeding. These results show that HLH-30 activation is critical for sustaining survival during starvation–refeeding stress via regulating TOR. Glucose and linoleic acid bypass the requirement for HLH-30 in coupling lysosome nutrient sensing to survival.

## Abstract 2

Lysosomes play a central role in coupling the nutrient state of the cell to growth and survival decisions. This study uncovers a critical role for HLH-30, the nematode ortholog of the mammalian MiT/TFE family of master regulators of lysosome biogenesis, in survival under starvation and refeeding conditions. Refeeding simple nutrients bypasses the requirement for HLH-30 to permit survival.

## Introduction

Lysosomes are membrane-bound subcellular organelles that contain degradative enzymes and are ubiquitously present in all eukaryotic cell types. These specialized organelles compartmentalize an acidified milieu for enzymatic degradation of complex macromolecules and organelles that are trafficked via autophagy for recycling intracellular substrates or taken up from the extracellular space via endocytosis, macropinocytosis, and phagocytosis. Transcription Factor EB (TFEB) and related members of the MiT/TFE (Microphthalmia-associated transcription factor) family of basic helix–loop–helix leucine zipper transcriptional activators (TFE3 and MiTF) were discovered as master regulators of autophagy and lysosome biogenesis programs by analyzing lysosomal gene promoters [1, 2]. TFEB and its family members are activated upon starvation and drive transcriptional up-regulation of autophagy–lysosome machinery genes to sustain survival of mammalian cells via ensuring continued nutrient availability by autophagic breakdown of lipid droplets and intracellular material within lysosomes [1, 3, 4].

Lysosomes play a critical role in sensing the cellular nutrient state and coupling the responses to activation of target of rapamycin (TOR), a nutrient-activated serine–threonine kinase that promotes anabolism and growth and suppresses catabolism [5]. Lysosomal nutrient sensing (LYNUS) also regulates TFEB activation. In the fed state, TFEB associates with the LYNUS complex on the cytoplasmic face of the lysosome, which harbors mammalian target of rapamycin complex 1 (mTORC1) linked to vacuolar ATPase (V-ATPase) via Ragulator [6]. The V-ATPase complex “senses” lysosomal levels of amino acids [6] and cholesterol [7] to transmit the “fed” signal via its interaction with Ragulator, initiating guanine nucleotide exchange factor (GEF) activity for Rag GTPases to activate mTOR. mTOR drives phosphorylation and inactivation of TFEB family members by inducing retention of these transcriptional activators in the cytoplasm [6, 8]. Upon starvation, activation of GTPase activity of RagA/B [9] facilitates Rag A degradation [10], whereby the mTOR-Ragulator complex dissociates and relieves the tonic phosphorylation of TFEB family members. Simultaneous lysosomal calcium channel opening activates calcineurin to dephosphorylate TFEB [11] (and presumably TFE3/MiTF) to unmask its nuclear localization signal, resulting in nuclear translocation where it activates transcription of lysosomal biogenesis and autophagy genes [12]. Importantly, during starvation stress, these transcriptional pathways maintain energy homeostasis via up-regulation of lysosomal acid lipases, which degrade lipid droplets sequestered by lipophagy [13]. Whether lysosomal lipolysis is primarily a source of energy generation or is also critical for generating survival signals remains to be elucidated.

Recent studies have identified a feedback loop whereby MiT/TFE family members transcriptionally up-regulate mTOR complex components during the starvation phase to permit mTOR reactivation upon refeeding [14] to channel the resumption of nutrient supply towards growth. Interestingly, prior studies in worms indicate that RNA polymerase accumulates at the promoters of growth genes in preparation for prompt activation of transcription upon resumption of nutrient supply [15]. Whether the MiT/TFE family members are critical for organismal survival with refeeding following starvation is not known.

The nematode *C*. *elegans* offers numerous advantages as a model system to evaluate the effects of starvation and refeeding on survival of the whole organism, given the various adaptations it has evolved to sustain survival in the face of nutrient deprivation [16]. One such phenomenon is the developmental arrest at the first larval stage (L1); upon hatching, L1 worms arrest postembryonic development when they fail to sense nutrients in their environment. Arrested L1 larvae can survive for weeks while retaining their ability to resume development and a normal life span upon refeeding [16]. Importantly, HLH-30 is the sole *C*. *elegans* ortholog of the mammalian MiT/TFE family of transcription factors [17], and animals with a loss of function of *hlh-30* display increased sensitivity to starvation stress [3, 18]. Taken together with the critical role for activation of MiT/TFE family members under starvation stress in mammalian cells [19], these data suggest that lysosomal function under starvation conditions is evolutionarily conserved. We modeled starvation stress in *hlh-30*–deficient worms and discovered that all attempts to rescue these animals by refeeding with bacteria, a complex food source, failed. Instead, refeeding with *C*. *elegans* maintenance medium (CeMM), a fully defined liquid that contains basic metabolites, rescued the starvation-induced lethality despite the absence of HLH-30. The critical ingredients of CeMM are glucose and lipids, especially linoleic acid (a polyunsaturated ω-6 fatty acid), since these nutrients were both necessary and sufficient to overcome *hlh-30* deficiency. Interestingly, CeMM refeeding promotes lysosomal acidification, restores the lysosomal compartment, and permits reactivation of TOR. Our findings indicate that in contrast to a prominent role for amino acids in LYNUS in mammals, glucose and lipids can be “sensed” to activate TOR signaling and resume growth after a period of starvation via pathways that bypass HLH-30 (and, by extension, the mammalian MiT/TFE family members).

## Results

### *hlh-30* was necessary for survival during starvation and recovery with refeeding

**Fig 1:**
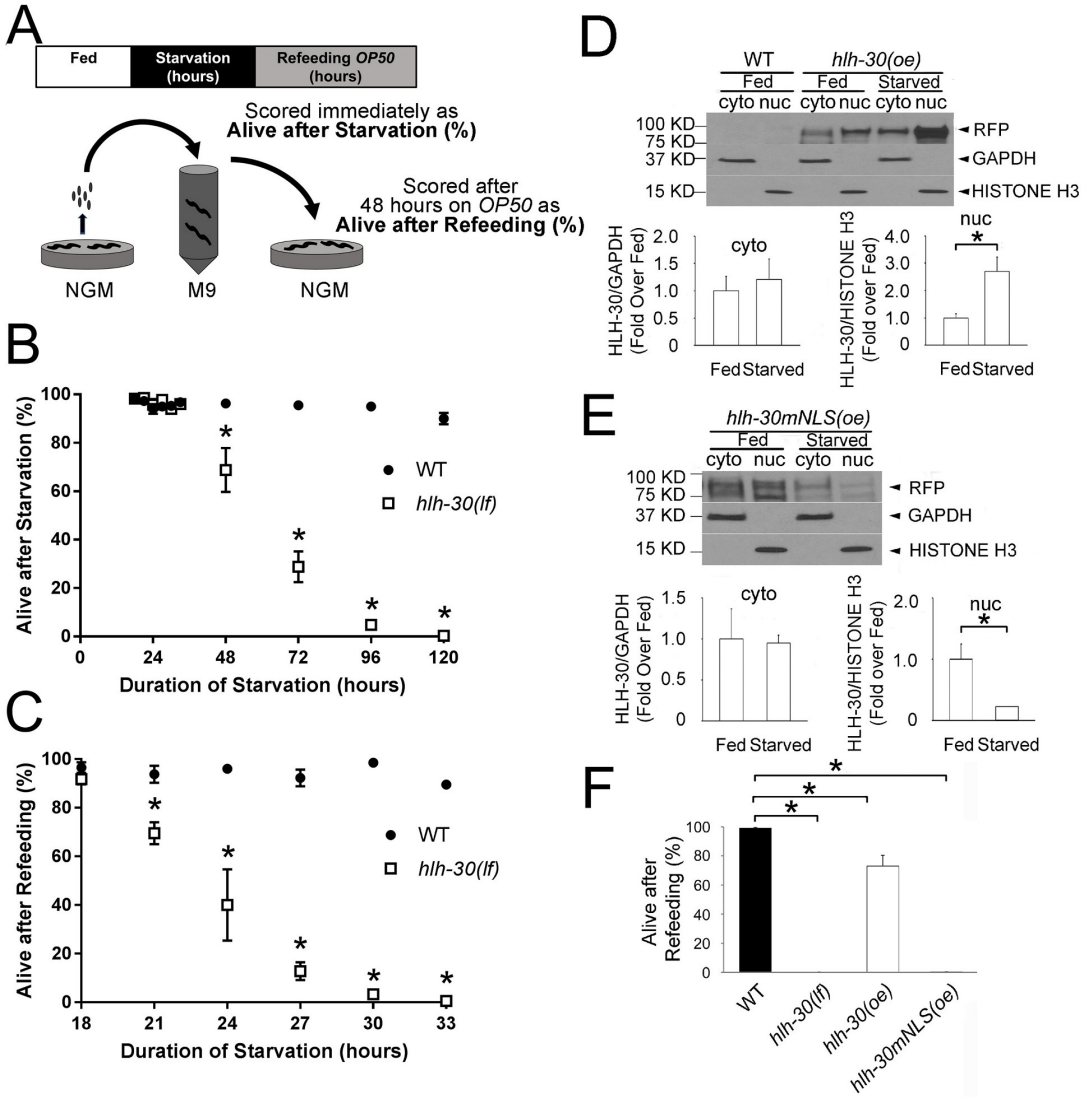
*hlh-30* was necessary for survival during starvation and upon refeeding, and starvation promotes HLH-30 nuclear localization. (A) Schematic depicting starvation and refeeding assay. Gravid adults were bleached with alkaline hypochlorite to obtain eggs, which were transferred to buffered salt solution (M9). Upon hatching, larvae arrest development at the L1 stage. Aliquots of starved L1 worms were placed on NGM dishes seeded with *E*. *coli* (*OP50*), and worms were immediately scored as alive or dead based on spontaneous movement; the fraction alive were defined as “Alive After Starvation.” After two days, worms were again scored as alive or dead based on spontaneous movement; the fraction alive compared to two days previously were defined as “Alive After Refeeding.” Because “Alive After Starvation” and “Alive After Refeeding” are calculated using different denominators, either value may be higher. (B, C) WT and *hlh-30(lf)* worms were analyzed after variable periods of starvation (*N* = 4 biological replicates with 50 worms/time point; values indicate mean ± SEM). *P < 0.05 by post hoc test for comparison between the two genotypes at the indicated time points following two-way ANOVA. (D, E) Animals that overexpress RFP fused to WT *hlh-30(oe)* or to *hlh-30(mNLS)(oe)* were analyzed at the L1 stage in the fed state or after 33 hours of starvation. Protein extracts were separated to obtain cyto and nuc fractions. Representative immunoblots (above) illustrate protein levels, and quantification of HLH-30 levels in the cytosol (normalized to GAPDH levels, fed state set equal to 1.0) and nucleus (normalized to Histone H3 levels, fed state set equal to 1.0) are shown below. *N* = 3 biological replicates/group. Bars indicate mean ± SEM. *P < 0.05 by *t* test. Lanes 1 and 2 of the immunoblot in panel D contain extracts from WT worms lacking RFP expression and document the specificity of RFP detection. (F) WT, *hlh-30(lf)*, *hlh-30(oe)*, and *hlh-30(mNLS)(oe)* worms were analyzed after 33 hours of starvation for “Alive after Refeeding.” *N* = 3 biological replicates/group of approximately 50 worms. Bars indicate mean ± SEM. *P < 0.05 by post hoc test after one-way ANOVA. Raw data for B–F are in S1 Data. cyto, cytoplasmic; GAPDH, glyceraldehyde 3-phosphase dehydrogenase; *hlh-30*, basic helix–loop–helix transcription factor 30; *hlh-30(mNLS)(oe)*, overexpressed HLH-30 with a mutant nuclear localization signal; *hlh-30(lf)*, loss-of-function *tm1978* mutation *hlh-30*; *hlh-30(oe)*, overexpressed HLH-30; L1, first larval stage; NGM, nematode growth medium; nuc, nuclear; RFP, red fluorescent protein; SEM, standard error of the mean; WT, wild type.

To understand the role of *hlh-30* in sustaining survival during starvation and preparing for refeeding, we modeled starvation-induced arrest of L1 stage worms deficient in *hlh-30* using the strong loss-of-function *tm1978* mutation (a deletion allele lacking two exons, heretofore termed as *hlh-30(loss-of-function)* or *hlh-30(lf)*; S1 Table) [18]. Upon hatching in a nutrient-free salt solution, L1 worms were starved for variable times, and aliquots of starved worms were refed on nematode growth medium (NGM) dishes containing *Escherichia coli OP50*. Survival was scored immediately by visual observation for spontaneous movement, defined as alive after starvation (Fig 1A). Fig 1B shows that periods of starvation of 48 hours or more significantly increased lethality in *hlh-30(lf)* worms. By contrast, wild-type worms were able to withstand nutrient deficient conditions for much longer (Fig 1B, S1A Fig).

To determine the role of *hlh-30* in recovery upon refeeding, we examined survival in worms refed on *E*. *coli OP50* dishes following a sublethal duration of starvation (Fig 1A and 1C). Importantly, starved wild-type worms demonstrated near complete recovery upon refeeding despite starvation lasting up to 10 days (Fig 1C, S1B Fig; see S1 and S2 Movies depicting wild-type worms after starvation for 33 hours and subsequent refeeding with *E*. *coli OP50* for 48 hours, respectively), demonstrating the remarkable ability of L1-arrested worms to withstand starvation, as previously described [16]. In contrast, while *hlh-30(lf)* worms survived short durations of starvation (18–48 hours, Fig 1B; see S3 Movie depicting mutant worms after starvation for 33 hours), refeeding was unable to rescue them, with fully penetrant lethality observed after only 33 hours of starvation (Fig 1C; see S4 Movie depicting *hlh-30(lf)* worms starved for 33 hours and refed with *E*. *coli OP50* for 48 hours). This finding suggests critical roles for *hlh-30* in sustaining survival during starvation as well as priming for recovery following refeeding with bacteria.

Given previous observations that HLH-30 signaling is essential in the innate immune response to infection [20], we examined the possibility that starved *hlh-30(lf)* worms are sensitized to *E*. *coli* pathogenicity by feeding animals with UV-inactivated bacteria. Dead *E*. *coli* supported the growth of starved wild-type worms but failed to rescue starved *hlh-30(lf)* mutants (S2A Fig). *C*. *elegans* may prefer other bacteria as a food source such as *Comamonas* [21]. Feeding *Comamonas* also did not support recovery of starved *hlh-30(lf)* worms (S2B Fig). Also, starved *hlh-30(lf)* worms were not rescued by liquid medium containing concentrated *OP50* (S-medium) (S2C Fig), indicating that multiple sources of complex nutrients cannot be accessed by starved *hlh-30* mutant worms to permit recovery following refeeding.

To explore the defect in *hlh-30(lf)* worms, we examined physiological responses to starvation and refeeding. One such possibility is a failure of developmental arrest, such as observed during starvation in *daf-16(lf)* mutants (with loss of function of *daf-16*, the *C*. *elegans* ortholog of the FOXO Forkhead transcription factor family) that display rapid death [22, 23]. As a marker of developmental progression, we examined the division of the precursor M cell that occurs upon transition from the L1 to second larval (L2) stage in starved L1 worms carrying the integrated transgene, a transcriptional fusion of basic helix–loop–helix transcription factor 8 promoter and GFP (*phlh-8*::*gfp*) [24]. All L1 worms examined demonstrated only one green fluorescent protein (GFP)-expressing cell after 33 hours of starvation, confirming that *hlh-30(lf)* and wild-type worms undergo and sustain L1 arrest upon starvation at this time point (S3A Fig). The rate of pharyngeal pumping is reduced during starvation and restored upon refeeding to permit uptake of food following starvation [25]. The rate of pharyngeal pumping one hour after refeeding with *E*. *coli OP50* was comparable in starved *hlh-30(lf)* and wild-type worms (S3B Fig), suggesting that pharyngeal dysfunction is not the cause of death upon refeeding. To determine whether *hlh-30(lf)* worms were capable of ingesting bacteria upon refeeding following starvation, we refed starved worms with a mixture of *E*. *coli OP50* and fluorescent microspheres of the same diameter as the average bacterium. Multiple fluorescent microspheres were observed in the intestinal lumen of mutant animals (S3C Fig). Importantly, we did not observe any evidence of live bacteria within the intestines of starved *hlh-30(lf)* mutants refed with GFP-expressing *E*. *coli OP50* (S3D Fig), ruling out a pharyngeal defect in crushing bacteria that could allow intestinal colonization and pathogenicity [26]. These data indicate that the failure of the *hlh-30(lf)* worms to recover upon refeeding following starvation is not due to a physiological defect such as inability to arrest development or failure to ingest food.

### *hlh-30* was necessary for transcriptional activation of multiple genes and maintenance of energy stores during starvation–refeeding stress

HLH-30 target genes have been identified that encode for the autophagy–lysosomal machinery and lysosomal enzymes [18, 27]. We examined a panel of 13 such genes, and they displayed several different patterns of regulation (S4 Fig). Lysosomal genes such as lysosome membrane protein 1 (*lmp-1*) (S4B Fig), cysteine protease related 1 (*cpr-1*) (S4G Fig), and aspartyl protease 1 (*asp-1*) (S4H Fig) and autophagy pathway genes such as autophagy yeast Atg homolog 18 *atg-18* (S4K Fig) and *atg-9* (S4M Fig) displayed significantly lower levels in starved *hlh-30(lf)* mutant animals compared to starved wild-type animals, indicating that *hlh-30* is necessary to promote the expression of these genes during starvation. Importantly, of these genes, only *atg-18* and *atg-9* displayed significantly higher levels in starving wild-type animals compared to fed wild-type animals. In contrast, genes such as *hlh-30* (S4A Fig), sulfatase domain proteins *sul-1* (S4C Fig) and *sul-3* (S4E Fig), *cpr-1* (S4G Fig), related to yeast vacuolar protein sorting factor 11 *vps-11* (S4I Fig), and the ortholog of mammalian LC3, GABARAP and GATE-16 family *lgg-1* (S4L Fig) displayed significantly higher levels following *E*. *coli OP50* refeeding in starved wild-type animals compared to fed wild-type animals. *hlh-30* was necessary for *sul-3* and *cpr-1* to display these higher levels. Thus, while *hlh-30* was necessary to sustain the expression of some target genes during starvation and refeeding, this analysis did not uncover a gene that was dependent on *hlh-30* for its up-regulation during starvation as well as refeeding with *E*. *coli OP50* and could explain the lethality of *hlh-30(lf)* mutants under these conditions.

To investigate additional target genes that could play a mechanistic role in the *hlh-30* phenotype, we performed a whole-transcriptome–wide RNA sequencing (RNAseq) analysis followed by Kyoto Encyclopedia of Genes and Genomes (KEGG) pathway analysis for significantly regulated genes in the wild-type and *hlh-30(lf)* groups subjected to 33 hours of starvation (S5A Fig). While pathways of fatty acid metabolism, degradation, and biosynthesis of unsaturated fatty acids were altered in both genotypes, the *hlh-30(lf)* mutant displayed changes in genes relevant to multiple additional metabolic pathways (S5A Fig, group B on the heat map in S5B Fig; also see S2 Table). Unsupervised hierarchical clustering revealed lower levels of oxidative phosphorylation and lysosome gene transcripts in the starved *hlh-30(lf)* worms compared to the wild type (groups B and C on the heat map in S5B Fig; also see S2 Table). Taken together with the established role for HLH-30 (and TFEB) signaling in induction of autophagy to generate energy from breaking down intracellular components during starvation [4, 18], these data point to energetic insufficiency in *hlh-30(lf)* mutants as the potential cause for observed lethality with starvation. To investigate this, we measured ATP levels in worm lysates. In fed animals, ATP levels were not significantly different between *hlh-30(lf)* and wild-type worms (ATP levels in nmol/mg DNA: 62 ± 3 in *hlh-30(lf)* versus 51 ± 11 in the wild type, *P* = not significant [NS], *N* = 3/group). However, *hlh-30(lf)* worms starved for 33 hours displayed a marked energy deficit compared to starved wild-type worms (ATP levels in nmol/mg DNA: 37 ± 2 in *hlh-30(lf)* versus 171 ± 4 in the wild type, *P* < 0.001 by *t* test, *N* = 3/group), despite both being alive at this time point (Fig 1B; S1 and S3 Movies). Importantly, while starved wild-type worms displayed higher levels of oxidative phosphorylation, lysosomal, and fatty acid metabolism gene transcripts compared with their fed counterparts (group D on heat map in S5B Fig; S2 Table), starved *hlh-30(lf)* worms displayed higher levels of amino-acid metabolism gene transcripts and a different set of lysosomal gene transcripts compared with their fed counterparts (group E on heat map in S5B Fig; S2 Table), indicating induction of alternative metabolic pathways as compensatory mechanisms in the absence of *hlh-30* signaling.

### Nuclear localization of HLH-30 was necessary to rescue the starvation–refeeding lethal phenotype

To investigate the role of *hlh-30* nuclear translocation in conferring starvation resistance, we performed rescue experiments in *hlh-30(lf)* worms. Wild-type HLH-30 or a mutant protein deficient in nuclear translocation (based upon homology with mammalian TFEB protein [28]) was fused with a C-terminal red fluorescent protein (RFP). Twelve transgenic lines expressing wild-type HLH-30::RFP and two transgenic lines expressing mutant nuclear localization signal HLH-30(mNLS)::RFP were obtained. One transgenic line from each group was selected for further analysis based on a similar level of robust expression of *hlh-30* transcripts (approximately 8-fold over wild-type levels, S6A Fig, S3 Table), henceforth referred to as *hlh-30(oe)* or *hlh-30(mNLS)(oe)* (overexpression abbreviated as “oe”). Fluorescence microscopy revealed that starvation caused HLH-30::RFP to translocate to the nucleus, whereas HLH-30(mNLS)::RFP remained in the cytoplasm (S6B Fig). Biochemical fractionation confirmed that HLH-30::RFP protein levels increased in the nuclear fraction of starved worms compared with fed worms (Fig 1D) without a change in cytosolic HLH-30::RFP levels. In contrast, levels of the nuclear localization signal mutant, HLH-30(mNLS)::RFP, were reduced in the nuclear fraction of starved worms as compared with their fed counterparts (Fig 1E). This indicates starvation-induced nuclear translocation of the wild-type HLH-30::RFP protein, which induces its own transcription in an autoregulatory manner, whereas the mutant HLH-30(mNLS)::RFP protein levels declined in the nuclear fraction and were perhaps not replenished because of loss of autoregulation [18]. Expression of the wild-type HLH-30::RFP protein rescued the starvation–refeeding lethality in *hlh-30(lf)* worms (Fig 1F, S6C Fig); expression of similar levels of the mutant HLH-30(mNLS)::RFP protein did not result in rescue, suggesting that survival during starvation is dependent on nuclear translocation of HLH-30. The observed rescue was not complete, which might result from reduced activity of the HLH-30::RFP fusion protein or an inability of the transgene to restore HLH-30 to its endogenous level or timing.

### Simple nutrients can rescue the starvation-induced lethality in *hlh-30*–deficient worms

**Fig 2:**
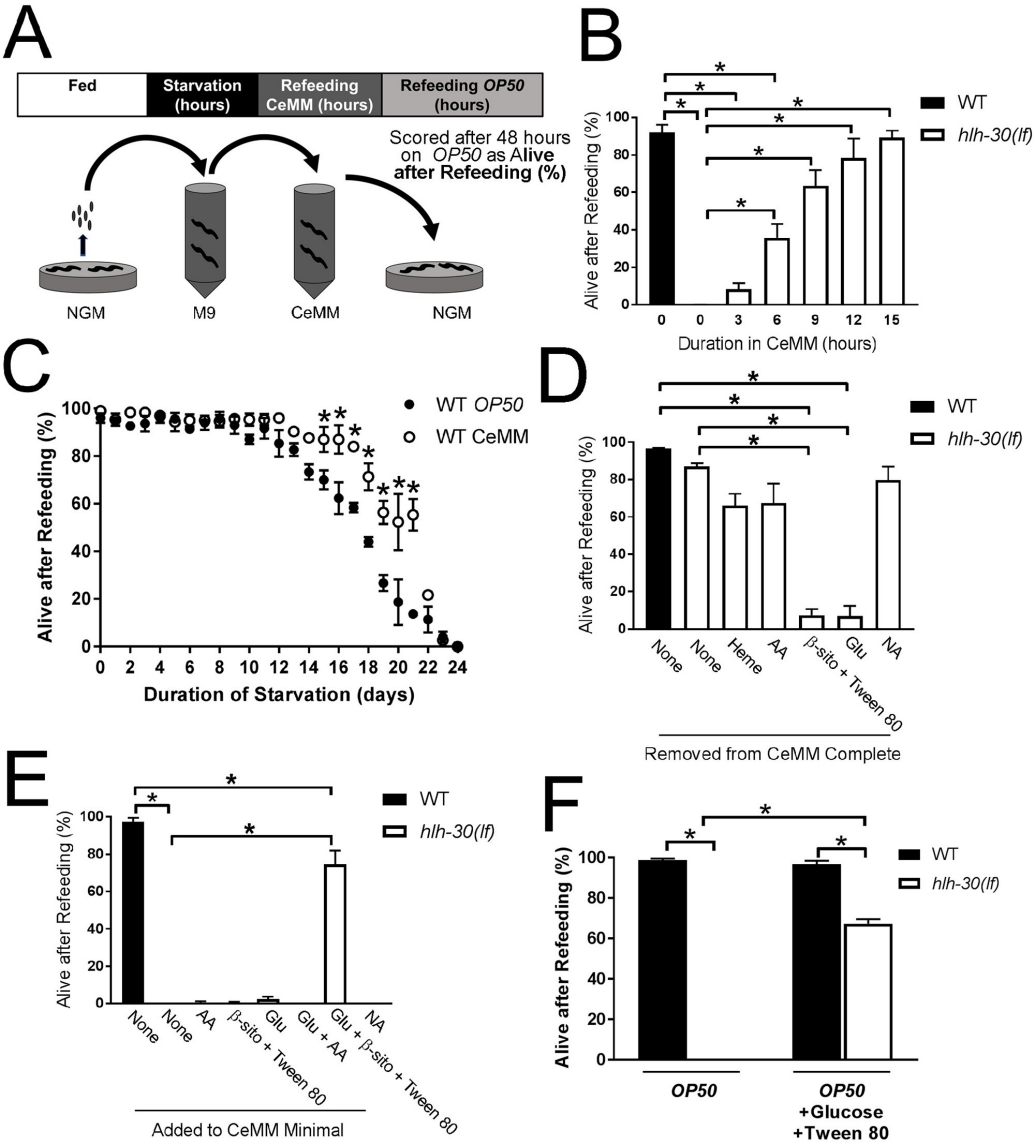
CeMM rescued impaired survival in starved *hlh-30(lf)* worms. (A) Schematic depicting the starvation/refeeding protocol with the addition of a variable period of CeMM exposure after starvation in M9 and before transfer to NGM dishes with *E*. *coli OP50* (see Fig 1A legend for details). (B) WT and *hlh-30(lf)* worms were analyzed after 33 hours of starvation and variable periods of CeMM exposure followed by 48 hours on *E*. *coli OP50* as “Alive after Refeeding.” *N* = 3 biological replicates with 50 worms/time point for all experiments. Bars indicate mean ± SEM. *P < 0.05 compared to the WT by post hoc test after one-way ANOVA. (C) WT worms were starved for the indicated duration of time (on the *y*-axis) and refed with *E*. *coli OP50* or CeMM and analyzed for “Alive after Refeeding,” as described in in the legend for Fig 1A. *N* = 3 biological replicates with approximately 50 worms/time. Values indicate mean ± SEM. *P < 0.05 by post hoc test after two-way ANOVA. (D, E) WT and *hlh-30(lf)* worms were analyzed after 33 hours of starvation and 15 hours of exposure to modified formulations of CeMM followed by 48 hours on *E*. *coli OP50* as “Alive after Refeeding.” For removal experiments (panel D), otherwise complete CeMM was prepared lacking heme, AAs, β-sito in Tween 80, Glu, or NAs. For addition experiments (panel E), CeMM minimal solution lacking Glu, β-sito in Tween 80, AAs, and NAs was supplemented with individual nutrients. *N* = 3 biological replicates with 50 worms/time point for all experiments. Bars indicate mean ± SEM. *P < 0.05 compared to WT by post hoc test after one-way ANOVA. (F) WT and *hlh-30(lf)* worms were analyzed for “Alive after Refeeding” after 33 hours of starvation and 48 hours of refeeding with standard *E*. *coli OP50* or *E*. *coli OP50* supplemented with glucose and Tween 80. Bars indicate mean ± SEM. *N* = 3 biological replicates/group with approximately 50 worms/condition. *P < 0.05 by post hoc test after two-way ANOVA. Raw data for B–F are in S1 Data. AA, amino acid; CeMM, *C*. *elegans* maintenance medium; Glu, glucose; *hlh-30*, basic helix–loop–helix transcription factor 30; *hlh-30(lf)*, loss-of-function *tm1978* mutation *hlh-30*; NA, nucleic acid; NGM, nematode growth medium; SEM, standard error of the mean; WT, wild type; β-sito, β-sitosterol.

Dysfunction of autophagy or the LYNUS complex results in reduced levels of simple metabolites, such as amino acids and sugars, and lethality in mouse pups under conditions of starvation [29, 30]. Interestingly, supplying simple nutrients such as glucose or amino acids alone results in modest prolongation of survival but does not rescue these animals from starvation-induced lethality [29, 30]. By analogy to these observations, we reasoned that refeeding *hlh-30(lf)* worms with bacteria may not be sufficient for survival because critical metabolites required for recovery are not generated from breakdown of complex substrates as a result of defective lysosomes in this mutant. To test the hypothesis that a mixture of simple nutrients can bypass defective lysosomes and rescue the lethality of *hlh-30(lf)* worms, we employed *C*. *elegans* maintenance medium (CeMM), a fully defined chemical medium that contains various simple nutrients and is sufficient to sustain growth and development [31]. When starved worms were incubated in CeMM prior to exposure to complex bacterial food, the starvation–refeeding lethality was rescued (Fig 2A and 2B; also see S5 and S6 Movies depicting wild-type and *hlh-30(lf)* worms starved for 33 hours, followed by 15 hours of CeMM treatment and subsequent *E*. *coli OP50* refeeding for 48 hours). Rescue was time dependent such that 3–6 hours of incubation caused partial recovery, whereas 9–15 hours of incubation caused complete recovery (Fig 2B). Incubation with CeMM was effective in rescuing *hlh-30(lf)* worms starved for as long as 4 days but was no longer effective after 6 days of starvation (S7 Fig). This observation suggests that the simple nutrients in CeMM provide metabolites that promote recovery in mutant worms, whereas wild-type worms may derive similar or identical metabolites from lysosomal breakdown of complex substrates. Intriguingly, wild-type worms also derived a modest survival benefit with CeMM after a prolonged period of starvation (Fig 2C), suggesting that brief periods of starvation in *hlh-30(lf)* mutants and prolonged periods of starvation in wild-type animals may result in similar deficits.

To determine which component of CeMM is necessary for rescue, we systematically removed each major class of macronutrients. Removal of heme, amino acids, or nucleic acids did not significantly impair recovery, indicating these factors are not necessary, whereas removal of lipids or carbohydrates abolished rescue, indicating these factors are necessary (Fig 2D). To determine which component(s) are sufficient for rescue, we added each major class of macronutrients to a minimal preparation of CeMM. Solutions with only amino acids, nucleic acids, lipids, or carbohydrates were not sufficient to promote rescue, but minimal CeMM containing the two necessary components, carbohydrates and lipids, was sufficient for recovery (Fig 2E).

### CeMM restores tricarboxylic acid cycle and linoleic acid metabolites in starved *hlh-30(lf)* worms

To elucidate the mechanisms whereby CeMM rescued starved *hlh-30(lf)* worms, we performed global metabolomic profiling in starved and CeMM-refed worms. As shown in S8A Fig, unsupervised hierarchical clustering accurately segregated the various groups based upon their metabolomic signature. Interestingly, random forest analysis identified complex lipid metabolites (along with glucose) to be highly enriched in the subset of metabolites (24/30) that accurately distinguished CeMM-refed *hlh-30(lf)* worms (that ultimately survive) from starved *hlh-30(lf)* worms (that do not survive, S8B Fig). We determined that the effective carbohydrate is glucose, and substitution of glucose with nonmetabolizable monosaccharides, L-glucose and 2-deoxy-D-glucose, did not promote rescue (S9A Fig).

**Fig 3:**
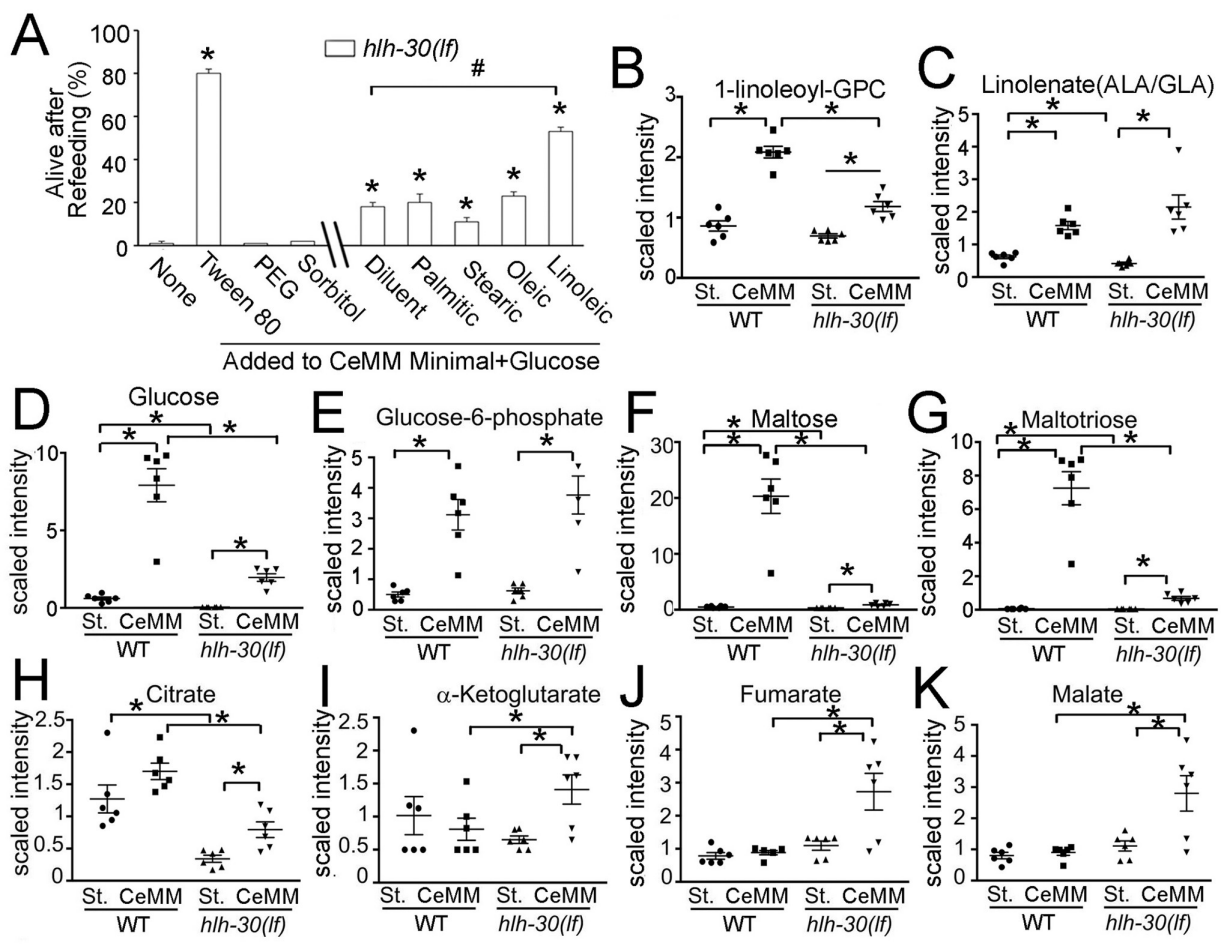
Metabolomic profiling reveals linoleic acid, a CeMM component, as essential for rescue of starved *hlh-30(lf)* worms. (A) *hlh-30(lf)* worms were analyzed after 33 hours of starvation and 15 hours of exposure to modified formulations of CeMM, followed by 48 hours on *E*. *coli OP50* (as in Fig 2A). CeMM minimal solution lacking glucose, β-sitosterol in Tween 80, amino acids, and nucleic acids was supplemented with glucose and individual nutrients shown below. *N* = 3 replicates of 50 worms each. A mixture of BSA and ethanol was used to solubilize fatty acids and tested as a control (labeled as diluent). Bars indicate mean ± SEM. *P < 0.05 versus none and #*P* < 0.05 versus diluent by post hoc test after one-way ANOVA. (B–K) WT and *hlh-30(lf)* worms were subjected to 33 hours of starvation (St.) or worms were refed with CeMM for 15 hours following the 33 hours of starvation (CeMM). Abundance of 1-linoleoyl-GPC (B), linolenate (C, both α- and γ-linolenic acid, ALA or GLA), glucose (D), glucose-6-phosphate (E), maltose (F), and maltotriose (G) and tricarboxylic cycle metabolites, namely citrate (H), α-ketoglutarate (I), fumarate (J), and malate (K) was assessed as scaled intensity (please see Methods for details). *N* = 6 biological replicates/group. Bar and whisker indicate mean ± SEM. *P < 0.05 by post hoc test after two-way ANOVA. Raw data for A–K are in S1 Data. BSA, bovine serum albumin; CeMM, *C*. *elegans* maintenance medium; *hlh-30*, basic helix–loop–helix transcription factor 30; *hlh-30(lf)*, loss-of-function *tm1978* mutation *hlh-30*; linoleoyl-GPC, linoleoyl glycerolphosphocholine; SEM, standard error of the mean; WT, wild type.

To better characterize the nature of the lipid contribution to the rescue, we performed additional control experiments. *C*. *elegans* requires exogenous cholesterol [32], and CeMM contains β-sitosterol dissolved in Tween 80 to meet this requirement. Surprisingly, β-sitosterol was not necessary for CeMM to promote recovery of the *hlh-30(lf)* worm (S9B Fig). Rather, Tween 80 was crucial for recovery along with glucose, and Tween 80 exhibited a dose-dependent effect (S9C Fig). Moreover, supplementation of glucose and Tween 80 to *E*. *coli OP50* on NGM dishes was sufficient to rescue starved *hlh-30(lf)* worms (Fig 2F). According to the manufacturer, Tween 80 is a PEGylated sorbitan bound with an ester linkage to a lipid moiety that may be derived from oleic, linoleic, palmitic, or stearic acid. To determine which lipid component of Tween 80 promotes rescue, we delivered glucose and each lipid component separately in CeMM. Linoleic acid, an 18-carbon ω-6 polyunsaturated fatty acid, significantly promoted rescue (Fig 3A). By contrast, oleic acid, an 18-carbon ω-9 monounsaturated fatty acid, or the two saturated fatty acid components, namely palmitic (16-carbon) and stearic acid (18-carbon), did not significantly promote rescue compared with the diluent ethanol. Ethanol, which can itself be utilized by wild-type worms as a nutrient under starvation conditions [33], conferred modest rescue in combination with glucose (Fig 3A).

This metabolomics analysis showed that linoleic acid moieties are enriched among lipids (7/24) in the subset of metabolites with the highest importance to group separation in the random forest analysis (S8B Fig; see S4 Table for the list of metabolites evaluated). Also, the abundance of linoleoyl glycerolphosphocholine (linoleoyl-GPC), the metabolite with the highest importance to group separation (S8B Fig), was significantly increased in refed *hlh-30(lf)* worms compared to their starved counterparts (Fig 3B). While linoleate levels were not different (S10 Fig), the abundance of α- and γ-linolenate (α- and γ-FFA; downstream metabolites of linoleic acid) was significantly increased in refed *hlh-30(lf)* worms compared to starved *hlh-30(lf)* worms (Fig 3C), indicating that administered linoleic acid was being metabolized. Taken together, these findings indicate that ω-6 fatty acids are required (along with glucose) to confer survival in the setting of *hlh-30* deficiency under starvation conditions.

In wild-type worms, CeMM feeding resulted in increased levels of glucose and its metabolite, glucose-6-phosphate, as well as of maltose and maltotriose (Fig 3D–3G), indicating a shift in glucose metabolism towards glycogen storage (also see S11 Fig). Increased levels of myristoleate and palmitoleate indicate stimulation of anabolic functions such as de novo fatty acid synthesis (see S10 Fig and S4 Table). In contrast, starved *hlh-30(lf)* mutants demonstrated reduced glucose, maltose, and maltotriose levels compared to the starved wild type (Fig 3D, 3F and 3G), consistent with substrate depletion in the face of energy crisis (reflected in the approximately 5-fold reduction in ATP stores; vide supra). Citrate levels were also significantly reduced in starved *hlh-30(lf)* mutants compared with the starved wild type (Fig 3H), along with lower levels of oxidative phosphorylation gene transcripts (S2 Table), which was reversed with provision of CeMM. Also, these metabolic changes were accompanied by increased levels of other tricarboxylic acid cycle (TCA) metabolites with CeMM refeeding of starved *hlh-30(lf)* mutants (α-ketoglutarate, fumarate, and maleate, Fig 3I–3K). The increased level of glucose with CeMM refeeding was not as dramatic in starved *hlh-30(lf)* mutants compared to the wild type (Fig 3D) and was accompanied by increases in glucose-6-phosphate (Fig 3E) and other glycolytic intermediates (S11 Fig). Similarly, while glycogen metabolites displayed higher levels with CeMM refeeding in starved *hlh-30(lf)* mutants, they continued to be markedly reduced compared with CeMM-refed wild type (Fig 3F and 3G; S11 Fig). Importantly, the levels of a majority of these metabolites were significantly lower in *E*. *coli OP50*-refed *hlh-30(lf)* mutant worms after starvation compared to CeMM-refed animals (S12 Fig). This difference in metabolites correlates with the probability of survival, which is zero percent in *E*. *coli OP50*-refed versus near complete in CeMM-refed *hlh-30(lf)* mutant worms (as described in Figs 1C and 2B). Taken together, these data point to an increased flux of exogenously supplied glucose through glycolysis and the TCA to generate energy in the starved *hlh-30(lf)* worms that permits survival. Indeed, CeMM refeeding led to the restoration of ATP levels, indicating that CeMM corrected the marked energetic insufficiency observed in starved *hlh-30(lf)* worms (ATP levels in nmol/mg DNA: 108 ± 11 in CeMM-refed *hlh-30(lf)* versus 72 ± 15 in CeMM-refed wild type, *P* > 0.05, *N* = 3/group). Also, while levels of diacylglycerols, endocannabinoids, and phospholipids robustly rebounded in CeMM-refed versus starved wild-type worms, comparable increases were not observed in starved *hlh-30(lf)* mutants upon CeMM feeding (S13 and S10 Figs, and S4 Table). Levels of amino acids and markers of protein breakdown were increased in CeMM-refed wild-type worms but not in CeMM-refed *hlh-30(lf)* mutants, consistent with impaired autophagic protein breakdown during starvation with *hlh-30* deficiency (S13 Fig). Interestingly, nucleic acid metabolism after refeeding in wild-type worms appeared to be aimed at salvage and recycling of nucleotides and their precursors, whereas evidence for increased breakdown of nucleotides (increased 3-ureidopropionate and β-alanine levels) was more prevalent in *hlh-30(lf)* mutants after refeeding (S14 Fig and S4 Table). These results suggest that although CeMM enabled survival of *hlh-30(lf)* mutants, it did not restore a metabolic phenotype identical to the wild type.

### *lipl-2*, an HLH-30 target, encodes a lysosomal lipase that was necessary for starvation resistance and recovery upon refeeding

**Fig 4:**
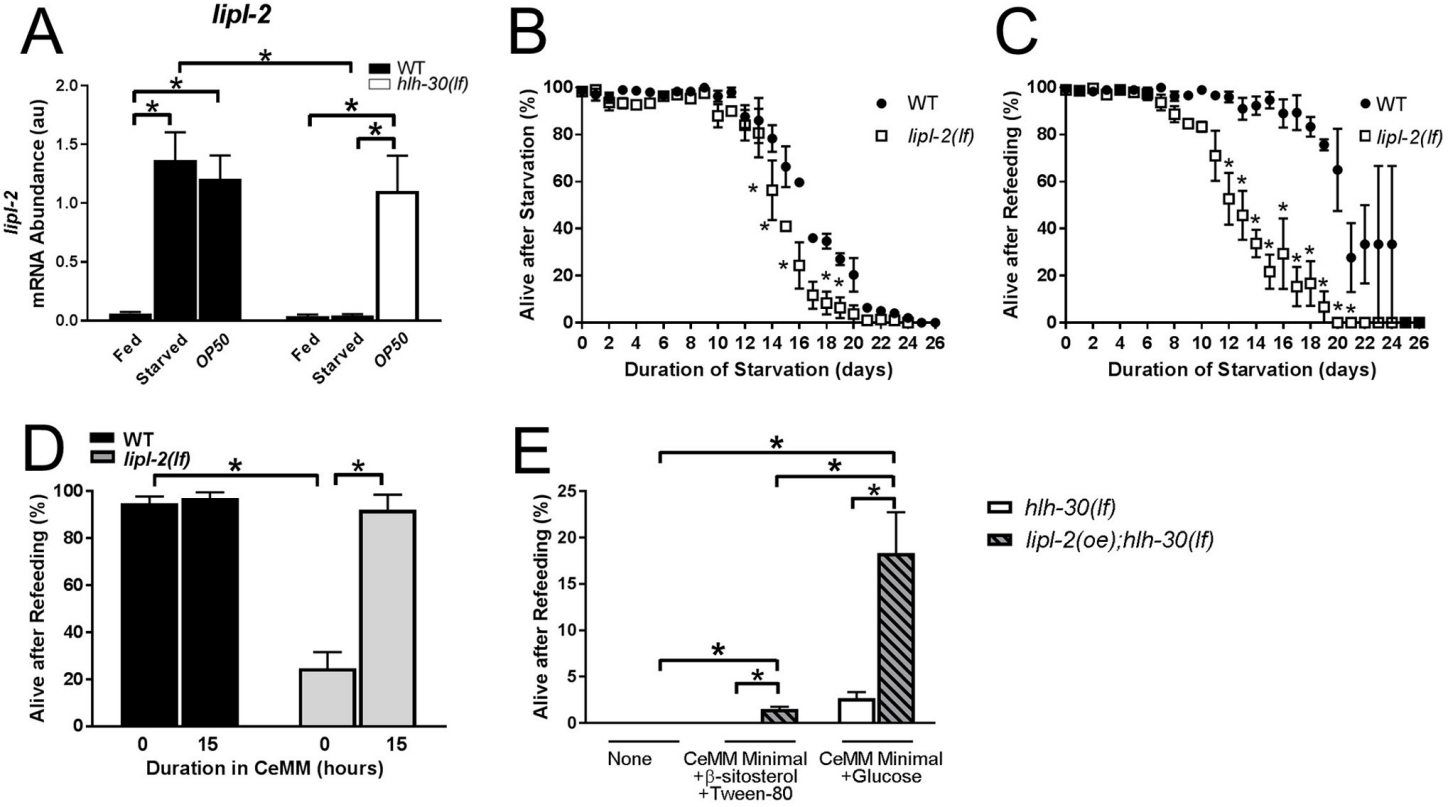
Worms deficient in *lipl-2*, an HLH-30 target gene, displayed starvation–refeeding mortality that was rescued by CeMM. (A) *lipl-2* mRNA abundance in au with values normalized to the control gene *ama-1* determined by qPCR in L1 stage WT and *hlh-30(lf)* animals in the fed state (fed), after starvation for 33 hours (starved), and after starvation for 33 hours followed by refeeding on *E*. *coli OP50* for 15 hours (*OP50*). *N* = 3 biological replicates/group. *P < 0.05 by post hoc test after two-way ANOVA. (B, C) “Alive after Starvation” (B) and “Alive after Refeeding” (C) as described in Fig 1A for *lipl-2(lf)* mutant animals and WT controls. *P < 0.05 versus WT by post hoc test after two-way ANOVA. (D) WT and *lipl-2(lf)* worms were analyzed after 10 days of starvation and 0 or 15 hours of complete CeMM exposure, followed by 48 hours on *E*. *coli OP50*. *P < 0.05 by post hoc test after two-way ANOVA. (E) Survival of *hlh-30(lf)* worms with *lipl-2* overexpression (*lipl-2(oe);hlh-30(lf)*) and *hlh-30(lf)* (as controls) analyzed after 33 hours of starvation followed by exposure for 15 hours to CeMM minimal solution (lacking glucose, β-sitosterol in Tween 80, amino acids, and nucleic acids) supplemented with β-sitosterol in Tween 80 (lipids) or glucose, followed by 48 hours on *E*. *coli OP50*. Similarly modeled worms without exposure to CeMM are shown as control. *P < 0.05 by post hoc test after two-way ANOVA. *N* = 3 biological replicates/group with 50 worms/time point for panels B–D. Bars and values indicate mean ± SEM. Raw data for A–E are in S1 Data. *ama-1*, amanitin-binding subunit of RNA polymerase II; au, arbitrary unit; CeMM, *C*. *elegans* maintenance medium; *hlh-30*, basic helix–loop–helix transcription factor 30; *hlh-30(lf)*, loss-of-function *tm1978* mutation *hlh-30*; *lipl-2*, lysosomal lipase 2; *lipl-2(lf)*, loss-of-function mutation *lipl-2*; *lipl-2(oe);hlh-30(lf)*, *hlh-30(lf)* with overexpressed *lipl-2*; L1, first larval stage; qPCR, quantitative PCR; SEM, standard error of the mean; WT, wild type.

Our data indicate that a source of lipids, i.e., linoleic acid, was required for the rescue with CeMM in *hlh-30*–deficient worms. TFEB and HLH-30 play important roles in lysosomal lipolysis during starvation stress [3, 18], and our whole-transcriptome–wide RNAseq profiling indicates significant regulation of lipid metabolism genes in the starved worms (S5 Fig; S2 Table). Accordingly, we examined the expression of lysosomal lipase genes and found that starvation induces higher levels of *lipl-2* (but not *lipl-1*, *3*, *4*, and *5*) in an *hlh-30*–dependent manner (Fig 4A, Supplementary S15A–S15D Fig). To investigate the functional significance of this response, we examined the starvation sensitivity of worms deficient in *lipl-1*, *2*, *3*, and *4* isoforms with the available deletion mutants (see S1 Table). The *lipl-2(tm4324)* mutation is a deletion of multiple exons likely to cause a strong loss of function (*lipl-2(lf)*); these worms displayed impaired survival during starvation compared to wild-type worms (Fig 4B) and a marked defect in recovery with refeeding (Fig 4C). Worms deficient in *lipl-1*, *lipl-3*, and *lipl-4* were indistinguishable from the wild type in these assays (S15E and S15F Fig).

Remarkably, CeMM was sufficient to rescue the starvation–refeeding defect observed in *lipl-2(lf)* worms (Fig 4D). Taken together, these observations suggest that *lipl-2* plays a role in generating critical lipid metabolites in wild-type animals that are starved and refed, and supplementation with linoleic acid in the absence of *hlh-30* signaling can substitute for the function of *lipl-2*. Accordingly, we evaluated whether overexpressed *lipl-2* (*lipl-2(oe)*) could rescue *hlh-30(lf)* worms. *lipl-2(oe)* did not rescue the lethality of *E*. *coli OP50*-refed *hlh-30(lf)* worms after 33 hours of starvation (Fig 4E). This is consistent with the observation that *E*. *coli OP50* increases expression levels of *lipl-2* (as well as *lipl-1*, *3*, *4*, and *5*; see Fig 4A, S15A–S15D Fig) in *hlh-30(lf)* worms but does not confer survival. However, *lipl-2(oe)* was sufficient to confer a modest rescue of starved *hlh-30(lf)* worms when CeMM minimal was supplemented with glucose (approximately 18% survival), which was significantly greater than the rescue observed when CeMM minimal was supplemented with lipids (approximately 2% survival; Fig 4E). We also determined whether *hlh-30* overexpression was sufficient to rescue starvation lethality of *lipl-2(lf)* animals. *hlh-30(oe);lipl-2(lf)* animals displayed a small but significant rescue compared to *lipl-2(lf)* animals, but they were still impaired compared to the wild type (S16 Fig). Overexpression of *hlh-30* may regulate alternative pathways that partially overcome the deficit in lysosomal lipolysis. Taken together, these observations indicate that lysosomal lipolysis is essential but not sufficient for surviving starvation and recovering upon refeeding.

Prior studies indicate that 20-carbon polyunsaturated fatty acids (PUFAs) accumulate in starved *C*. *elegans* [34], and recent work points to activation of autophagy signaling by 20-carbon ω-6 (but not ω-3) PUFAs in conferring life span extension in the *lipl-4* transgenic worms [35], suggesting that starvation-induced lipolysis may confer starvation resistance via PUFA-induced autophagy. To examine for autophagic structures and evaluate cellular ultrastructure, we employed transmission electron microscopy. The intestinal brush border and mitochondrial morphology in the starved *hlh-30(lf)* worms was similar to their fed counterparts and wild-type controls (S17 Fig). Although autophagic structures (both autophagosomes and autolysosomes) were detectable in the starving *hlh-30(lf)* worms, there appeared to be fewer, and the number appeared to be restored by CeMM refeeding (S17 Fig). To determine whether induction of autophagy signaling plays a role in the beneficial effects of linoleic acid, we used RNAi to reduce the activity of the *C*. *elegans* ortholog of human BECN1 (*bec-1*), a gene critical for autophagosome formation [36, 37]. Reducing *bec-1* mRNA levels by more than 90% did not attenuate (and even enhanced) the beneficial effect of CeMM supplementation in starved *hlh-30(lf)* worms (S18A–S18C Fig).

### CeMM restored proton pump components and acidified lysosomes in *hlh-30(lf)* worms

**Fig 5:**
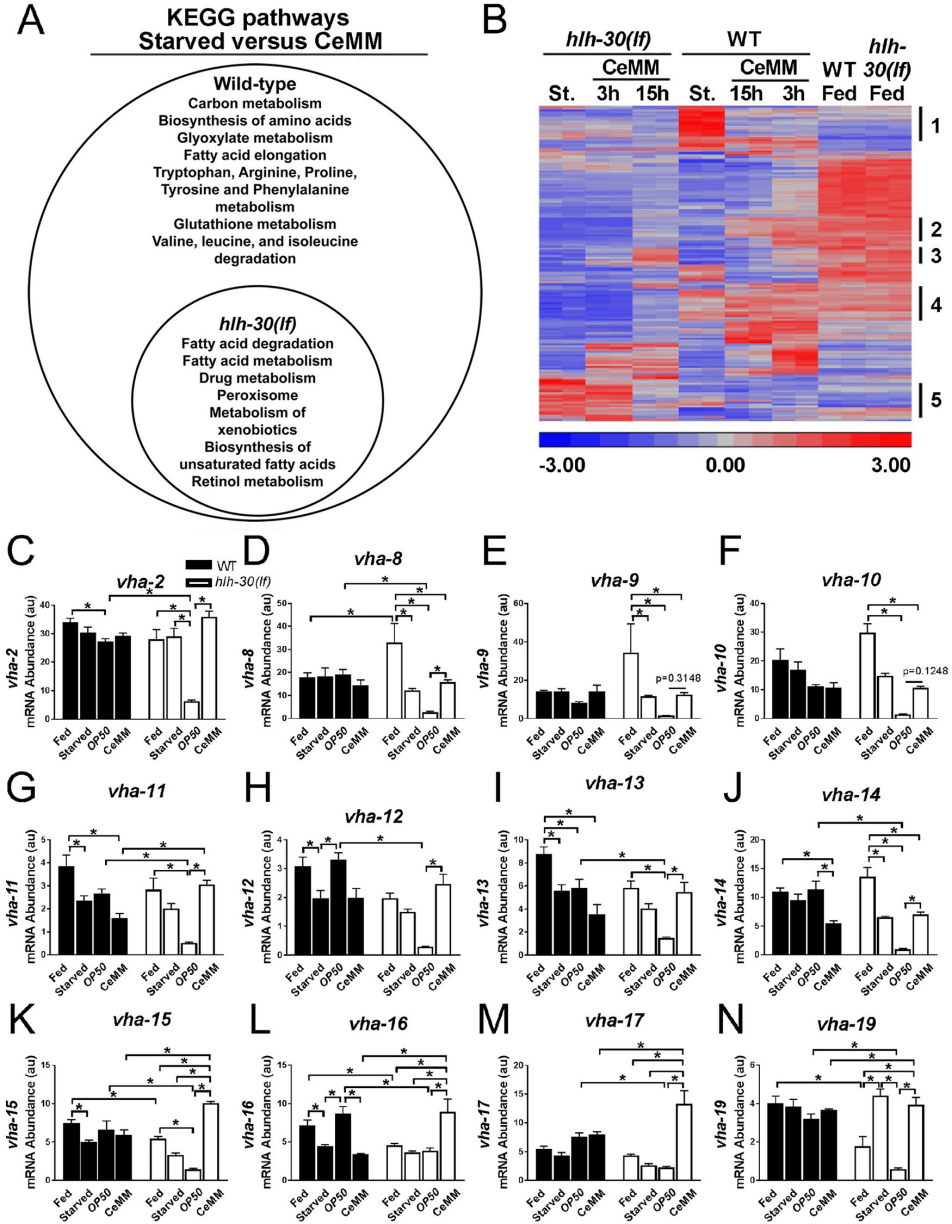
CeMM restored expression of *vha* genes in starved *hlh-30(lf)* mutants. (A) Venn diagram depicting significantly regulated KEGG pathways (both up-regulated as well as down-regulated; see S5 Table for details) by RNAseq analysis in WT and *hlh-30(lf)* L1 worms starved for 33 hours followed by incubation in CeMM for 0 or 15 hours versus starved worms in respective groups. *N* = 2 biological replicates/group. (B) Unsupervised hierarchical clustering of significantly altered transcripts in WT and *hlh-30(lf)* L1 worms starved for 33 hours followed by incubation in CeMM for 0, 3, or 15 hours or their fed counterparts. *N* = 2/group. Lists of genes in rows marked as 1–5 are presented in S5 Table. (C–N) mRNA abundance in au with values normalized to the control gene *ama-1* determined by qPCR for genes encoding for proton pump subunits (as named) in L1 stage WT and *hlh-30(lf)* animals in the fed state (fed), after starvation for 33 hours (starved), and after starvation for 33 hours followed by refeeding on *E*. *coli OP50* or CeMM for 15 hours. *N* = 3–8 biological replicates/group. Bars indicate mean ± SEM. *P < 0.05 by post hoc test after two-way ANOVA. Raw data for C–N are in S1 Data. *ama-1*, amanitin-binding subunit of RNA polymerase II; au, arbitrary unit; CeMM, *C*. *elegans* maintenance medium; *hlh-30*, basic helix–loop–helix transcription factor 30; *hlh-30(lf)*, loss-of-function *tm1978* mutation *hlh-30*; KEGG, Kyoto Encyclopedia of Genes and Genomes; L1, first larval stage; qPCR, quantitative PCR; RNAseq, RNA sequencing; SEM, standard error of the mean; *vha*, vacuolar H^+^-ATPase; WT, wild type.

To elucidate how CeMM rescues starvation lethality in *hlh-30(lf)* worms, we compared gene expression changes in CeMM-refed worms at 0, 3, and 15 hours using RNAseq profiling and identified significantly regulated KEGG pathways (Fig 5A and 5B). Refeeding with CeMM up-regulated a broad range of biosynthetic pathways in wild-type worms (Fig 5A), indicating resumption of growth with availability of nutrients. In contrast, starved *hlh-30(lf)* worms only up-regulated a subset of these pathways focused upon lipid metabolism, indicating their potentially critical role in the observed rescue (Fig 5A). Unsupervised hierarchical clustering revealed multiple patterns of gene expression changes in CeMM-refed *hlh-30(lf)* mutants that were distinct from their wild-type counterparts (Fig 5B, S5 Table). We focused on the transcripts that displayed higher levels only in the *hlh-30(lf)* worms with 15 hours of CeMM exposure (and not with 3 hours of exposure) corresponding to the observed rescue (see group 3 from Fig 5B in S5 Table, also see Fig 2B). Of the 16 genes in group 3, 11 encode V-ATPase subunits of the lysosomal proton pump [38]. Quantitative PCR analysis was used to examine the expression of these *vha*-subunit genes in *hlh-30(lf)* and wild-type worms that were fed, starved, or refed CeMM or *E*. *coli OP50* (Fig 5C–5N). Two distinct patterns were observed: 1) *vha-8*, *vha-9*, and *vha-14* displayed significantly lower transcript levels in starved *hlh-30(lf)* worms compared to fed worms, but not in starved wild-type worms compared to fed worms (Fig 5D, 5E and 5J); 2) *vha-2*, *vha-8*, *vha-11*, *vha-12*, *vha-13*, *vha-14*, *vha-15*, and *vha-19* displayed significantly lower transcript levels in *E*. *coli OP50*-refed *hlh-30(lf)* worms compared to the *E*. *coli OP50*-refed wild type (Fig 5C, 5D, 5G, 5H, 5I, 5J, 5K and 5N). These patterns indicate that *hlh-30* is necessary to increase the expression of these genes during starvation stress or restore expression to wild-type levels with *E*. *coli OP50* refeeding following starvation. Importantly, 9/11 of these *vha* genes (except *vha-9* and *vha-10*) displayed significantly higher transcript levels in *hlh-30(lf)* mutants after CeMM refeeding compared to *E*. *coli OP50* refeeding (Fig 5C–5D and 5G–5N). Furthermore, treatment with glucose and linoleic acid was sufficient to significantly increase transcript levels of *vha* genes (S19 Fig). In contrast, 12/19 of the autophagy–lysosome pathway genes or lysosomal lipase genes displayed significantly lower transcript levels in CeMM-refed compared to *E*. *coli OP50*-refed *hlh-30(lf)* mutants (S20C, S20F, S20I–S20L and S20N–S20S Fig). Four of the remaining five genes displayed lower transcript levels in CeMM treated *hlh-30(lf)* mutants compared with the similarly treated wild type (S20B, S20G, S20H and S20M Fig). Taken together, these data point to a role for CeMM-induced up-regulation of the proton pump genes in the rescue of starving *hlh-30(lf)* mutant worms.

**Fig 6:**
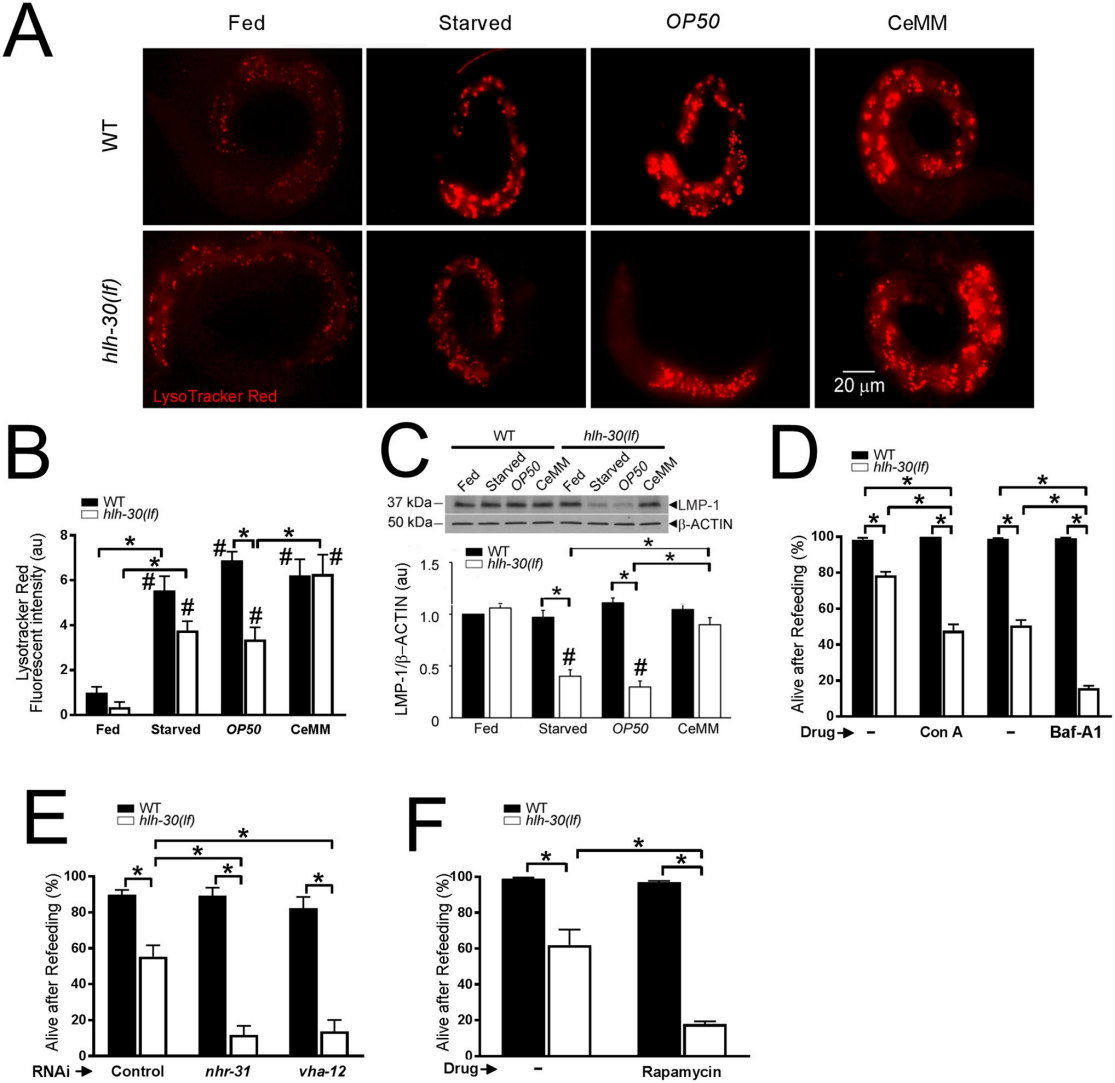
CeMM bypasses *hlh-30* to restore lysosome acidification and TOR activation and confer survival in *hlh-30(lf)* worms. (A, B) Representative fluorescence images (A) with quantification of fluorescence intensity (B) of LysoTracker Red staining. WT and *hlh-30(lf)* L1 worms were analyzed in the fed state (Fed) or after 33 hours of starvation (Starved) followed by 15 hours on *E*. *coli OP50*-seeded NGM dishes (*OP50*) or 15 hours of incubation in CeMM (CeMM). Scale bar is 20 μm. For panel B, WT fed value was set equal to 1.0, and other values were normalized to this value. *N* = 8–23 worms/condition. *P < 0.05 for comparisons as indicated by post hoc test after one-way ANOVA. #*P* < 0.05 versus respective fed state by post hoc test after two-way ANOVA. (C) Representative immunoblot (*top*) and quantification (*bottom*) of LMP-1 protein abundance (normalized to β-ACTIN as a loading control) in WT and *hlh-30(lf)* L1 worms cultured as described in A. WT fed value was set = 1.0. *N* = 5–7 biological replicates/group. *P < 0.05 for comparisons as indicated by post hoc test after two-way ANOVA. #*P* < 0.05 versus the respective fed state by post hoc test after one-way ANOVA. (D) Measurement of “Alive after Refeeding” as in Fig 1A in WT and *hlh-30(lf)* worms starved as L1 stage larvae for 33 hours, then transferred to CeMM for 15 hours in the presence of Con A or Baf-A1 with DMSO as control (depicted as “–”), and transferred to *E*. *coli OP50* dishes. DMSO concentrations employed to dissolve Con A and Baf-A1 were 0.34% and 1.2%, respectively. *N* = 4–5 biological replicates/group. *P < 0.05 by post hoc test after two-way ANOVA. (E) Assessment of “Alive after Refeeding” in WT and *hlh-30(lf)* worms starved as L1 stage larvae for 33 hours, transferred to CeMM containing dsRNA targeting *nhr-31*, *vha-12*, or L4440 as control for 15 hours, and transferred to *E*. *coli OP50* dishes for 48 hours. *N* = 4–9 biological replicates/group. *P < 0.05 by post hoc test after two-way ANOVA. (F) “Alive after Refeeding” measured as described in Fig 1A in WT and *hlh-30(lf)* worms starved as L1 stage larvae for 33 hours, then transferred to CeMM for 15 hours in the presence of rapamycin with DMSO (100%, employed as diluent; indicated as “–”) followed by transfer to *E*. *coli OP50* dishes containing rapamycin. *N* = 6 biological replicates/group. *P < 0.05 by post hoc test after two-way ANOVA. In all cases, bars indicate mean ± SEM. Raw data for B–F are in S1 Data. Baf-A1, bafilomycin A1; CeMM, *C*. *elegans* maintenance medium; Con A, concanamycin A; dsRNA, double-stranded RNA; *hlh-30*, basic helix–loop–helix transcription factor 30; *hlh-30(lf)*, loss-of-function *tm1978* mutation *hlh-30*; LMP-1, lysosome membrane protein 1; L1, first larval stage; NGM, nematode growth medium; *nhr-31*, nuclear hormone receptor 31; RNAi, RNA interference; SEM, standard error of the mean; TOR, target of rapamycin; *vha*, vacuolar H^+^-ATPase; WT, wild type.

We reasoned that higher transcript levels of proton pump genes caused by CeMM restores lysosome acidification and numbers in starved *hlh-30(lf)* worms, which could account for the observed rescue upon subsequent refeeding with bacterial food, a complex source of nutrients. To examine this premise, we performed staining with LysoTracker Red, an acidophilic dye, and evaluated expression of LMP-1, a lysosome membrane protein (Fig 6A–6C). LysoTracker Red expression was significantly increased in starved as well as refed worms of both genotypes compared with their respective fed state (Fig 6A and 6B). Importantly, LysoTracker Red expression was significantly lower in starved *hlh-30(lf)* worms refed with *E*. *coli OP50* compared to similarly treated wild-type worms (Fig 6A and 6B), and refeeding with CeMM was sufficient to restore LysoTracker Red expression to wild-type levels. Furthermore, LMP-1 levels were significantly lower in starved *hlh-30(lf)* worms compared to starved wild-type worms (Fig 6C). Refeeding with CeMM (but not *E*. *coli OP50*) restored LMP-1 abundance to wild-type levels in the *hlh-30(lf)* worm (Fig 6C), indicating that CeMM restores lysosome acidification and abundance, likely via bypassing the requirement for *hlh-30*.

To investigate the functional importance of *vha* genes, we used the specific V-ATPase inhibitors concanamycin A or bafilomycin A1 (Baf-A1) [39, 40]. Both drugs reduced survival of starved *hlh-30(lf*) worms refed with CeMM (Fig 6D). In addition, the method of RNAi was used to reduce the activity of *vha-12*, a proton pump gene that was restored to wild-type levels by CeMM, but not *E*. *coli OP50*, refeeding in starved *hlh-30(lf*) worms (Fig 5H). Reducing *vha-12* activity also attenuated the CeMM-mediated rescue of starved *hlh-30(lf)* worms refed with CeMM (Fig 6E), indicating the proton pump encoded by *vha-12* is necessary for this rescue. Interestingly, nuclear hormone receptor 31 (*nhr-31*), which encodes a *C*. *elegans* ortholog of mammalian hepatocyte nuclear factor 4α (HNF-4α) (with linoleic acid as an endogenous ligand [41]), has been identified as a specific transcriptional activator of *vha* gene transcription in worms [42]. RNAi targeting of *nhr-31* transcripts reduced CeMM-induced rescue of starved *hlh-30(lf)* mutants (Fig 6E), indicating the nuclear receptor encoded by *nhr-31* is necessary for this rescue. To further evaluate *nhr-31*, we determined whether overexpression of *nhr-31* was sufficient to rescue *hlh-30(lf)* mutants supplemented with either glucose or linoleic acid. A significant increase in survival was not observed, pointing to a critical role for both the nutrients (glucose and linoleic acid) in entraining *nhr-31* signaling to confer rescue (S21 Fig).

### CeMM bypasses the requirement for *hlh-30* to restore TOR activation in refed *hlh-30(lf)* worms to confer survival

In mammals, activation of TOR signaling on lysosomes in response to refeeding is mediated via sensing amino acids and/or cholesterol and is essential for survival and resumption of growth [7, 30, 43]. Recent studies have ascribed a critical role for TFEB in transcriptionally up-regulating RagD during starvation to permit mTOR activation with refeeding [14]. Our data demonstrate that while amino acids and cholesterol are not essential for conferring rescue in *hlh-30(lf)* mutants (Fig 2C, S9B Fig), two different classes of nutrients, glucose and linoleic acid, drive increased transcript levels of V-ATPase genes, which may facilitate LYNUS to TOR activation.

To investigate the role of TOR activation, we attempted CeMM rescue in the presence of rapamycin, a known inhibitor of TOR activity [44]. Treatment with rapamycin significantly attenuated CeMM-mediated rescue of the starved *hlh-30(lf)* mutants (Fig 6F), pointing to a critical role for TOR reactivation in survival of starved *hlh-30(lf)* mutants.

**Fig 7:**
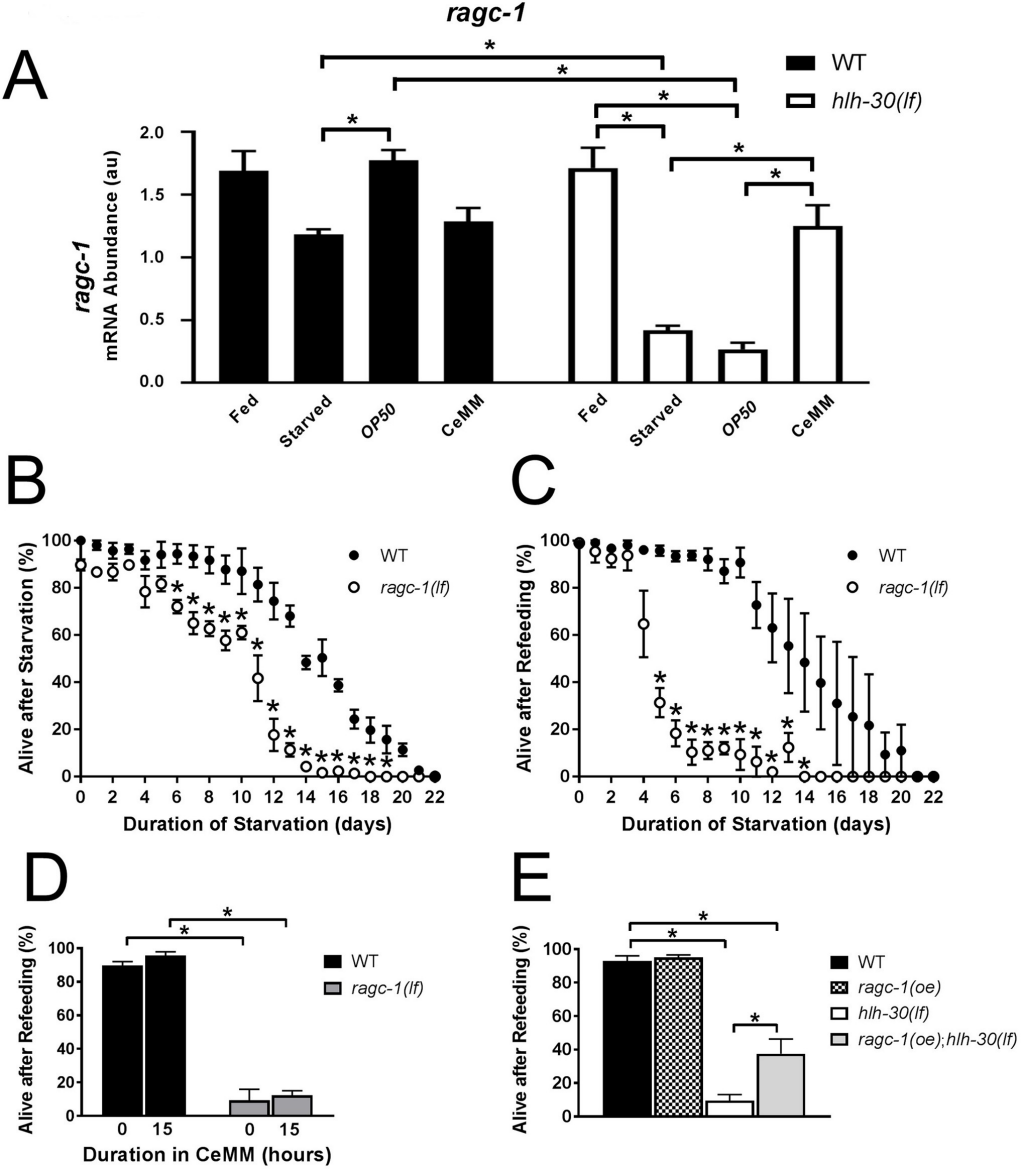
*ragc-1*–deficient worms were sensitive to starvation–refeeding stress, and *ragc-1* overexpression rescued *hlh-30(lf)*. (A) *ragc-1* mRNA abundance in au with values normalized to the control gene *ama-1* determined by qPCR in L1 stage WT and *hlh-30(lf)* animals in the fed state (fed), after starvation for 33 hours (starved), and after starvation for 33 hours followed by refeeding on *E*. *coli OP50* or CeMM for 15 hours. *N* = 6 biological replicates/group. *P < 0.05 by post hoc test after two-way ANOVA. (B, C) WT and *ragc-1(lf)* worms were analyzed for “Alive after Starvation” (B) and “Alive after Refeeding” (C) as described in the legend for Fig 1A. *N* = 3 biological replicates with approximately 50 worms/time point. *P < 0.05 by post hoc test after two-way ANOVA. (D) WT and *ragc-1(lf)* L1 stage worms were starved for 11 days and transferred to CeMM for 0 or 15 hours, followed by transfer to *E*. *coli OP50*-seeded NGM dishes for 48 hours. “Alive after Refeeding” was determined as described in the Fig 1 legend. *N* = 3 biological replicates/group with approximately 50 worms/time point. *P < 0.05 by post hoc test after two-way ANOVA. (E) “Alive after Refeeding” was measured as described in Fig 1A. A homogenous population of WT, *hlh-30(lf)*, and *hlh-30(lf);amEx324* (*ragc-1(oe);hlh-30(lf)*) worms that overexpress *ragc-1* from an extrachromosomal array were starved in M9 for 36 hours as L4 stage larvae, transferred to *E*. *coli OP50*-seeded NGM dishes, and scored for survival after 72 hours. *N* = 4 biological replicates. *P < 0.05 by post hoc test after two-way ANOVA. Bars and values indicate mean ± SEM. Raw data for A–E are in S1 Data. *ama-1*, amanitin-binding subunit of RNA polymerase II; au, arbitrary unit; CeMM, *C*. *elegans* maintenance medium; *hlh-30*, basic helix–loop–helix transcription factor 30; *hlh-30(lf)*, loss-of-function *tm1978* mutation *hlh-30*; *ragc-1(oe);hlh-30(lf)*, *hlh-30(lf)* worms that overexpress *ragc-1* from an extrachromosomal array; L1, first larval stage; L4, fourth larval stage; NGM, nematode growth medium; qPCR, quantitative PCR; *ragc-1*, the ortholog for mammalian RagC/D GTPases; *ragc-1(lf)*, loss-of-function mutation *ragc-1*; SEM, standard error of the mean; WT, wild type.

To investigate regulation of TOR pathway transcripts, we employed quantitative PCR. Transcripts for *let-363* (the *C*. *elegans* ortholog of mammalian mTOR, S22A Fig), *daf-15* (*C*. *elegans* ortholog of mammalian raptor, S22B Fig), *rsks-1* (the *C*. *elegans* ortholog of mammalian S6 kinase, a TOR target; S22F Fig), and *ragc-1* (the *C*. *elegans* ortholog of RAG-C/D required for TOR activation [14], Fig 7A) displayed significantly lower levels in starved *hlh-30(lf)* worms compared to the starved wild type. These findings suggest *hlh-30* is necessary to promote transcription of these genes during starvation. Importantly, refeeding with CeMM but not *E*. *coli OP50* restored the expression of *let-363* and *ragc-1* to wild-type levels in *hlh-30(lf)* mutants (S22A Fig and Fig 7A). We focused on the function of *ragc-1* because its regulatory control was strongly correlated with survival: *E*. *coli OP50* refeeding significantly increased *ragc-1* transcript levels in starved wild-type but not *hlh-30(lf)* worms, whereas CeMM exposure increased *ragc-1* transcript levels in *hlh-30(lf)* worms. We analyzed a strong loss-of-function *ragc-1(tm1974)* mutation caused by a deletion spanning multiple exons (S3 Table), termed *ragc-1(lf)*. *ragc-1(lf)* animals displayed striking sensitivity to starvation-induced lethality (Fig 7B). Refeeding either with *E*. *coli OP50* or CeMM did not restore viability (Fig 7C and 7D). Overexpression of *ragc-1* in *hlh-30(lf)* mutants was sufficient to confer partial rescue on starved animals fed *E*. *coli OP50* (Fig 7E, S23 Fig). Taken together, these data indicate that TOR activation was downstream of the observed CeMM-mediated rescue.

## Discussion

**Fig 8:**
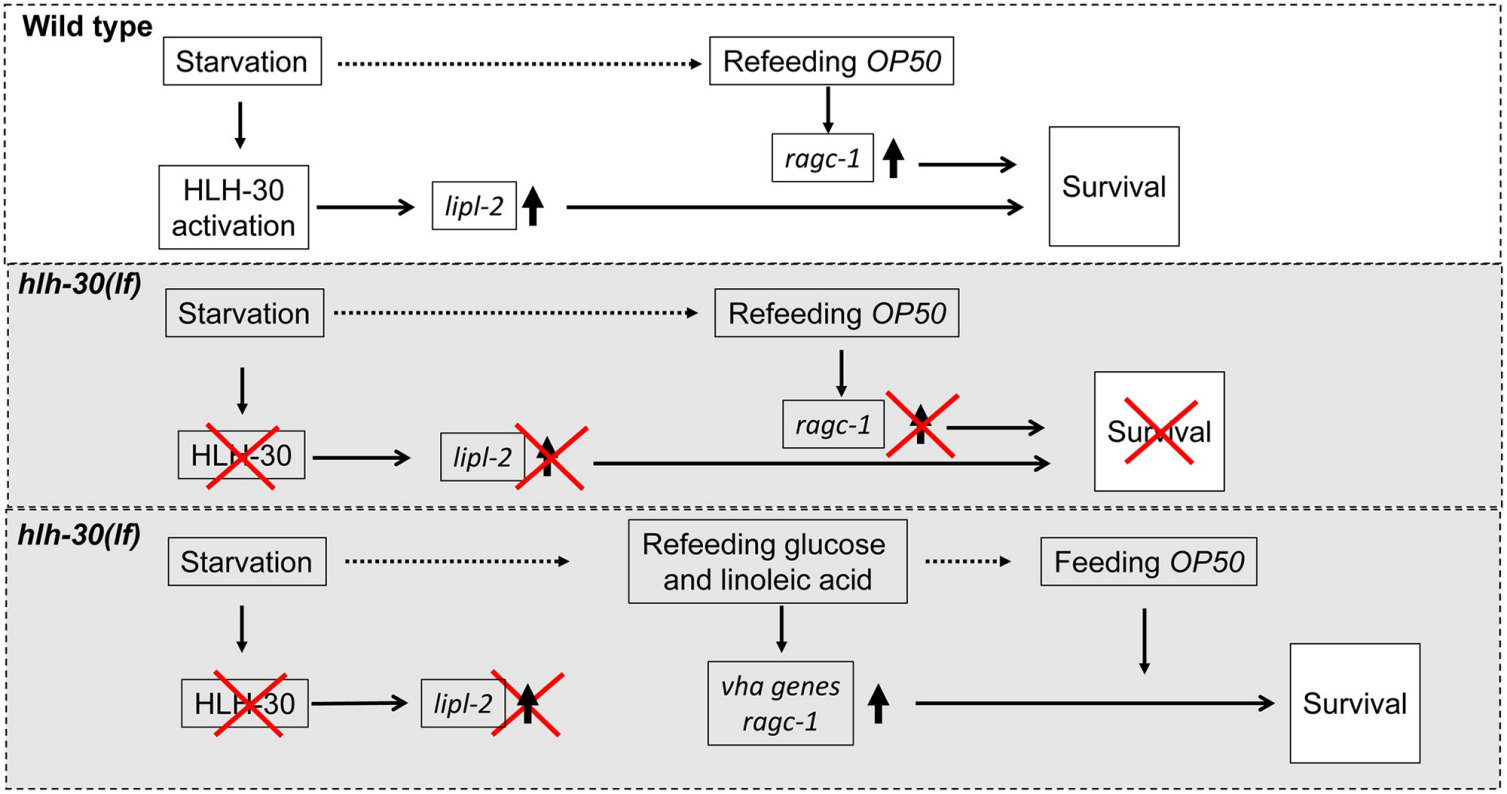
Schematic depicting pathways whereby simple nutrients bypass *hlh-30* to promote survival under starvation conditions. Under starvation stress, wild-type worms activate *hlh-30*–dependent expression of target genes such as *lipl-2* (a lysosomal lipase) via inducing cytosol to nuclear translocation of HLH-30 (*top panel*). *lipl-2* induction plays a critical role in starvation survival. Refeeding with *E*. *coli OP50* triggers increased *ragc-1* transcript levels, which couple nutrient sensing on acidified lysosomes and TOR activation to survival (*top panel*). In starved *hlh-30*–deficient worms, lack of HLH-30–induced transcription impairs *lipl-2* up-regulation and is accompanied by reduced *ragc-1* levels upon refeeding (*middle panel*). Starved *hlh-30*–deficient worms demonstrate marked energy deficiency with decreased lysosome abundance, leading to death despite refeeding with *E*. *coli OP50* (*middle panel*). Refeeding starved *hlh-30*–deficient worms with CeMM provides simple nutrients, namely glucose and linoleic acid, which restore ATP levels and induce *nhr-31*–mediated *vha* gene transcription (*bottom panel*). CeMM refeeding also restores *ragc-1* expression and lysosome acidification and abundance and sustains survival with subsequent *E*. *coli OP50* feeding (*bottom panel*). Impaired TOR activation with rapamycin treatment and loss of *ragc-1* prevent nutrient sensing to TOR upon refeeding following starvation, and rapamycin prevents CeMM rescue in starved *hlh-30*–deficient worms. Horizontal black arrows indicate causal relation. Horizontal dotted arrows indicate transition from starvation to refeeding. Upward arrows indicate increased levels. Red cross marks indicate absence of the indicated event. CeMM, *C*. *elegans* maintenance medium; *hlh-30*, basic helix–loop–helix transcription factor 30; *hlh-30(lf)*, loss-of-function *tm1978* mutation *hlh-30*; *lipl-2*, lysosomal lipase 2; *nhr-31*, nuclear hormone receptor 31; *ragc-1*, the ortholog for mammalian RagC/D GTPases; TOR, target of rapamycin.

Our studies have uncovered a novel, to our knowledge, pathway that bypasses *hlh-30* to drive *vha* gene transcription and couple lysosome nutrient sensing to TOR activation, which permits survival upon refeeding following starvation. The following lines of evidence support this claim. First, *hlh-30* deficiency markedly sensitizes worms to starvation-induced death, which cannot be rescued upon refeeding. Second, HLH-30 activation (nuclear translocation) is essential to permit survival with starvation and refeeding stress. Third, *hlh-30*–dependent induction of *lipl-2*, a lysosomal lipase, during starvation is critical to permit survival with starvation and refeeding stress. Fourth, exogenous delivery of glucose and linoleic acid restores energy stores, up-regulates *vha* gene transcription, restores lysosome acidification, and drives TOR reactivation to permit survival upon refeeding with complex nutrients in *hlh-30*–deficient and *lipl-2*–deficient worms. Fifth, knockdown of *nhr-31* (an upstream master regulator of *vha* gene transcription), knockdown of *vha-12* (a proton pump subunit), and pharmacologic inhibition of the proton pump or TOR activity inhibit the rescue observed with simple nutrients in starved and refed *hlh-30*–deficient worms. Sixth, the *ragc-1*–deficient mutant displays starvation–refeeding sensitivity and cannot be rescued with simple nutrients after starvation–refeeding stress. Seventh, forced activation of TOR with RAGC-1 overexpression is sufficient to rescue starved *hlh-30*–deficient worms upon refeeding. A model that emerges (Fig 8) indicates that HLH-30 activation during starvation stimulates induction of *lipl-2* and *ragc-1*, which play critical roles in facilitating survival—presumably via generation of lipid metabolites that act as signaling moieties to activate gene transcription—and in coupling lysosome nutrient sensing to TOR activation upon *E*. *coli OP50* refeeding, respectively. In *hlh-30*–deficient worms, exogenous provision of glucose and linoleic acid entrains an alternative transcriptional program via NHR-31 to bypass the requirement for HLH-30 to stimulate *vha* gene transcription, and CeMM induces *ragc-1* transcription to drive TOR activation and permit survival.

The novel, to our knowledge, observation that underlies our discovery is that a brief duration of starvation is sufficient to impair critical survival processes and prevent recovery despite refeeding in the *hlh-30*–deficient worms. Presumably, starved *hlh-30*–deficient worms cannot up-regulate autophagy, specifically lipophagy, as previously described [3, 18] to generate energy, and CeMM corrects the energy deficiency by providing glucose as fuel. However, while the non-metabolizable glucose equivalents (L-glucose and 2-deoxy-2-D-glucose) are unable to restore viability, D-glucose is insufficient by itself and requires Tween 80. Remarkably, our data point to an essential role for an ω-6 PUFA, linoleic acid (a component of Tween 80), for rescuing the consequences of lysosomal insufficiency during starvation, while a monounsaturated fatty acid, oleic acid, and two saturated fatty acids (palmitic and stearic acids) were not sufficient to confer rescue. This points to a selective deficiency of linoleic acid metabolites in the setting of *hlh-30* deficiency. Conceivably, HLH-30–stimulated *lipl-2* up-regulation generates the necessary metabolite/s via lysosomal lipolysis under starvation stress because loss of function of *lipl-2* mimics *hlh-30* deficiency. Intriguingly, starved worms are enriched in 20-carbon PUFAs (all are known to be linoleic acid derivatives) [45], namely arachidonic acid (AA), eicaso-pentaenoiec acid (EPA), and di-homo-gamma-linoleic acid (DGLA), and prior studies have established that the worm possesses the entire machinery to synthesize all species of required fatty acids (including linoleic acid) de novo [45]. However, our data suggest that *hlh-30(lf)* worms cannot generate sufficient linoleic acid from de novo synthesis under starvation conditions and require its supplementation exogenously, presumably because these worms lack the *hlh-30*–dependent transcriptional up-regulation of the fatty acid desaturase *fat* genes involved in de novo biosynthesis of unsaturated fatty acids, as described previously [3, 46], or are deficient in two-carbon precursors. These hypotheses will need to be tested in future studies. Also, consistent with the observation that glucose is required—presumably to correct deficient energy stores—but is insufficient to restore survival by itself, refeeding with *E*. *coli OP50* restored *lipl-2* expression in *hlh-30(lf)* worms but was also not sufficient to confer survival. Our findings also implicate a critical role for HLH-30 translocation to the nucleus because a mutant HLH-30 protein lacking the nuclear localization signal did not translocate to the nucleus and was ineffective in sustaining survival. This indicates a role of *hlh-30*–induced transcription of its target genes, such as *lipl-2* and *ragc-1*, in the observed response and is less likely to be due to other factors such as a potentially toxic role of cytosolic HLH-30 protein with a mutated nuclear localization signal.

A major finding of our study is that CeMM up-regulates proton pump (*vha* gene) transcripts and restores acidified lysosomes in the *hlh-30*–deficient worms. Furthermore, our studies implicate an important role for NHR-31, a nuclear hormone receptor, in transducing CeMM-mediated restoration of viability in starved *hlh-30*–deficient worms. NHR-31 is expressed in the worm intestine and excretory cell throughout development and identified as a master regulator of *vha* gene transcription and resistance to osmotic stress [42]. Interestingly, NHR-31 is one of the worm orthologs of the mammalian nuclear receptor HNF-4α, which has been described to bind linoleic acid as an endogenous ligand [41] and to control hepatic lipid metabolism via controlling peroxisome proliferator-activated receptor α (PPARα) signaling [47]. Our data indicate that CeMM activates *nhr-31* signaling to restore lysosome pH. This, along with CeMM-mediated restoration of lysosome abundance, facilitates LYNUS [12] and permits TOR activation in *E*. *coli OP50*-refed *hlh-30*–deficient worms. Indeed, refed *hlh-30*–deficient worms demonstrate growth on *E*. *coli OP50* only after prior incubation in CeMM because TOR activity is required for accessing nutrients to drive worm growth and development [48]. A recent study indicates that mammalian TFEB activation during starvation is essential to drive RAGD transcription during ongoing starvation to replenish the mTOR machinery and prepare the cells to switch from a primary catabolic to anabolic state [14]. Our data demonstrate that *ragc-1* transcript levels are significantly reduced by starvation–refeeding stress in the *hlh-30(lf)* mutant and are only rescued by CeMM treatment (but not by *E*. *coli OP50*). Accordingly, the increased sensitivity of the *ragc-1* mutant to starvation–refeeding stress demonstrates that TOR activation is essential as a final step for survival with refeeding following starvation that cannot be rescued by CeMM. Importantly, forced up-regulation of the TOR machinery by RAGC-1 overexpression was sufficient to rescue the obligate mortality in the starved *hlh-30*–deficient worms upon refeeding, attesting to a central role for lysosome nutrient sensing in survival in worms as observed in mammals [30]. The precise roles of TOR signaling in fostering survival likely involve its role in *C*. *elegans* growth and development and warrant careful experimental dissection of its multifaceted effects in future studies. Interestingly, amino acids that have been demonstrated to activate TOR signaling by both *rag*-dependent [43, 49] and *rag*-independent mechanisms [50] and cholesterol, which was also shown to be sufficient to activate TOR signaling [7], were unable to rescue starvation lethality, suggesting that transcriptional restoration of the lysosomal proton pump was a key upstream requirement in restoring lysosome nutrient sensing. Furthermore, the observation that CeMM can modestly increase survival of refed wild-type worms after a prolonged duration of starvation suggests that the CeMM-induced signaling pathway may be relevant to physiologic responses to starvation even in the *hlh-30*–sufficient state, a premise that should be further explored for potential therapeutic benefit in humans facing prolonged periods of fasting.

Recent studies indicate that lysosomal lipolysis generates signaling metabolites such as oleyl ethanolamine (OEA) by activation of *lipl-4* that binds to chaperones and activates nuclear signaling via NHRs, namely NHR-49 and NHR-80, to confer life span prolongation [51]. This process appears to be conserved in mammals since OEA has been demonstrated to be an endogenous ligand for PPARα, the mammalian ortholog of NHR-49 [52], and ablation of PPARα markedly increases sensitivity to fasting stress [53], pointing to a potentially critical role for lysosomal generation of lipid ligands to facilitate cytoprotective signaling during starvation/fasting stress. Lysosomal lipase signaling has also been implicated in conferring life span extension via NHR-49– and NHR-80–mediated suppression of vitellogenins [54], and knockdown of vitellogenin genes *vit-1* and *vit-5* led to a modest extension of duration of survival in starved *hlh-30* mutant worms [46]. Our data indicate that OEA levels are significantly reduced in starving *hlh-30* mutants compared to starving wild-type worms, but CeMM feeding induces only a modest up-regulation in the *hlh-30*–deficient worms in contrast to the increase observed in CeMM-refed wild type. Whether endogenous OEA generation or the NHR-49/NHR-80 signaling axis play a role in CeMM-mediated rescue of starved *hlh-30*–deficient worms and whether exogenous OEA can confer rescue in this setting remains to be determined.

Another mechanistic possibility whereby linoleic acid and PUFAs may confer survival signaling is via regulation of lipid stores, such as observed with deficiency of *daf-16*, which is essential to survive starvation because *daf-16*–deficient worms fail to undergo L1 arrest and die without exogenous nutrients [23]. Indeed, *daf-16* regulates expression of multiple genes involved in biosynthesis of unsaturated lipids, namely *fat-2*, *fat-6*, *fat-7*, and the fatty acid elongase *elo-2* [45, 55], and PUFAs can restore lipid stores in *daf-16* mutant worms [55], presumably via bypassing the requirement for these genes involved in biosynthesis of unsaturated lipids. Another possibility is that exogenous linoleic acid induces autophagy [35] in an *hlh-30*–independent fashion to overcome the autophagy deficiency under starvation conditions and confer rescue upon refeeding. Our data demonstrating that concomitant knockdown of *bec-1* gene expression does not prevent CeMM-mediated rescue rule out the role of canonical autophagy in this process. Interestingly, oleic acid, a PUFA, was sufficient to induce noncanonical *bec-1*–independent autophagy in *C*. *elegans* embryos and mammalian cells [56]. Whether linoleic acid can activate noncanonical autophagy signaling under conditions of lysosomal insufficiency remains to be determined.

The discovery of lysosomal metabolites with signaling roles and the ability to replenish them exogenously may have profound therapeutic implications in human disease with both genetic and acquired dysfunction of the lysosomal machinery. Indeed, lysosome storage diseases (LSDs) are inborn errors of metabolism clinically characterized by a “failure to thrive,” with generalized metabolic abnormalities beyond those expected from deficiency of individual lysosomal enzymes [57]. Multiple murine models of LSDs—namely mucopolysaccharidoses MPSI, MPSIIIB, MPSVII, Niemann-Pick type A/B, and infantile ceroid lipofuchsinosis (INCL or Batten disease)—display reduced adipose stores, reduced circulating free fatty acids and triglycerides [58], and reduced content of nearly all lipid metabolites examined in the liver tissue [59]. Careful experimental observations show that these effects are not due to reduced food intake, fat malabsorption, or adipocyte abnormalities [58]. Instead, the mutant mice displayed reduced content of simple sugars and evidence for increased protein breakdown [59], indicating a compensatory up-regulation of catabolism of other major macronutrient classes, likely as a source of energy. These observations support the emerging paradigm that the deficiency of individual lysosomal enzymes (or structural proteins) causes a generalized lysosomal dysfunction, which results in global dysregulation of lipid metabolism [60, 61]. In addition, disruption of lysosomal function has been implicated in multiple neurodegenerative diseases such as Alzheimer’s, Parkinson’s, and Huntington’s [62]. Our data raise the intriguing possibility that supplementation with simple sugars and PUFAs may ameliorate disease manifestation in these disorders, which requires further study.

## Methods

### Maintenance and source of *C*. *elegans* strains

All *C*. *elegans* strains were maintained at 20 °C on NGM dishes seeded with the *E*. *coli* strain *OP50*. The wild-type strain was Bristol N2. The mutant strains *hlh-30(tm1978)*, *lipl-1(tm1954)*, *lipl-2(tm4324)*, *lipl-3(tm4498)*, *lipl-4(tm4417)*, and *ragc-1(tm1974)* strains (please see S1 Table) were generously provided by the Mitani lab through the National BioResource Project of the MEXT, Japan. The *phlh-8*::*gfp* strain was generously provided by Dr. Toshiyaki Katada from the University of Tokyo, Tokyo, Japan, and crossed into *hlh-30(lf)*; see S3 Table. All strains were outcrossed three times to N2 before analysis. *E*. *coli OP50* and *Comomonas* were purchased from the *C*. *elegans* Genetic Center, University of Minnesota. To inactivate *E*. *coli OP50*, the bacteria were grown overnight on NGM dishes and exposed to UV light in a UV-Crosslinker (Stratagene, 2400; La Jolla, CA, USA) at 3,000 joules. A culture was subsequently inoculated in Luria broth (LB) and tested for growth at 37 °C on a shaker for 18 hours. The absence of bacterial growth confirmed inactivation.

### Starvation and refeeding assay

Gravid adults were bleached with alkaline hypochlorite to kill all contaminants and all worms not protected by the eggshell, then washed and placed in M9 [63, 64]. The volume of M9 was adjusted to yield a final density of four to six worms per μL. 15-ml polypropylene tubes containing the worms were shaken horizontally on a Labnet Orbit 1000 shaker (Labnet, Edison, NJ, USA) set at 100 revolutions per minute at 20 °C. At specific times, worms were placed on NGM dishes seeded with *E*. *coli OP50*, *Comamonas*, or UV-killed *E*. *coli OP50* or placed in S-medium containing *E*. *coli OP50* in suspension. The number of live worms, defined as worms exhibiting spontaneous movement, was scored by visual inspection immediately and after 48 hours. The metric “Alive after Starvation” was assessed as percentage of worms alive immediately upon placement on the food source, and the metric “Alive after Refeeding” was assessed as number of worms alive 48 hours later divided by the number of worms alive immediately upon placement on the food source. Fed worms were L1 stage larvae collected 18 hours after placement of a synchronized population of eggs on seeded NGM Petri dishes. Movies depicting spontaneous movement in these worms were obtained with a Leica M80 microscope (Leica, Wetzlar, Germany) with a 1.25× objective and an IC80 HD camera.

### Assessment of pharyngeal pumping

L1 worms were starved 33 hours in M9, then placed on NGM dishes seeded with *E*. *coli OP50* for one hour. Pharyngeal pumping was scored as described [65].

### Generation of *hlh-30(oe)* and *hlh-30(mNLS)* constructs

The plasmid used to generate *hlh-30(oe)* transgenic strains was generated by PCR amplification of a 1,497 base pair (bp) fragment containing the coding sequence for W02C12.3a from pDONR201 (Dharmacon, Lafayette, CO, USA) and a 2,000 bp fragment containing W02C12.3a promoter (Dharmacon) and subcloning these into the pBlueScript SK+ vector pDG219 to create *hlh-30p*::*hlh-30*::*rfp*. *hlh-30(oe)* carries the *amEx272* extrachromosomal array. The plasmid used to generate *hlh-30(mNLS)* transgenic strains was created by site-directed mutagenesis of the putative nuclear localization sequence found in *hlh-30p*::*hlh-30*::*rfp*, based on homology with the mammalian TFEB protein [28]. Specifically, codons for four amino acids were changed from arginine to alanine at positions 247, 248, 249, and 250 with the QuikChange Lightning kit (Stratagene, 210518–5), following the manufacturer’s instructions. *hlh-30mNLS(oe)* carries the *amEx291* extrachromosomal array. The co-marker pRF4 (*rol-6(su1006)*) was coinjected along with the *hlh-30(oe)* or *hlh-30mNLS(oe)* constructs. Please see the list of strains in S3 Table.

### Generation of RAGC-1 overexpressor in the *hlh-30*–deficient background

The *ragc-1(oe)* was made through microinjection of *ragc-1p*::*ragc-1*::*rfp* and the co-marker *sur-5*::*GFP* into the gonad arm of *hlh-30(lf)*. *ragc-1* was PCR amplified from genomic DNA to produce a 2,016 bp amplimer that extends 560 bp upstream of the translational start and 219 bp downstream of the translational stop codon. Constructs were subcloned into pBlueScript SK+, and the sequence was verified. Fed L4 worms were collected in 15-mL Falcon tubes, washed eight times with M9 to remove debris and bacteria, and starved in M9 at a density of one worm per μL. The 15-mL tubes were shaken horizontally on a Labnet Orbit 1000 at 100 revolutions per minute at 20 °C. An aliquot was taken every 24 hours, the worms were placed on NGM dishes, and the number of live worms compared to total worms in the aliquot was scored to assess “Alive after Starvation (%).” *ragc-1(oe)* transgenic animals were identified by expression of GFP prior to the starvation–refeeding assay. Worms were starved for 36 hours, then refed by placement on NGM dishes seeded with *OP50*. The number of live worms was scored upon refeeding 72 hours later and scored as Alive After Refeeding (as a percentage of worms alive at *T* = 0 upon refeeding). This extrachromosomal array was named *amEx324*. Please also see the list of strains in S3 Table.

### Generation of LIPL-2 overexpression strain in the *hlh-30(lf)* background

The *lipl-2(oe)* was made through microinjection of *lipl-2p*::*lipl-2*::*rfp* and the co-marker pRF4 (*rol-6(su1006)*) into the gonad arm of *hlh-30(lf)*. *lipl-2* was generated through PCR amplification of fosmid DNA. The construct extends 1,468 bp upstream of the ATG translational start site and 1,631 bp downstream to include the unspliced *lipl-2* genomic sequence without the stop codon. This construct was subcloned into pDG219, a modified pBlueScript SK+ plasmid containing RFP and 3′ UTR of *unc-54*. Please also see the list of strains in S3 Table.

### Generation of HLH-30 overexpression strain in the *lipl-2(lf)* background

The plasmids used to generate *hlh-30(oe)* transgenic strain and the co-marker pRF4 (*rol-6(su1006)*) were injected into the gonad arm of *lipl-2(tm4324)* to generate *hlh-30(oe);lipl-2(lf)*. Please also see the list of strains in S3 Table.

### Generation of NHR-31 overexpression strain in the *hlh-30(lf)* background

gBlock fragments (Integrated DNA Technologies, Coralville, IA, USA) containing the proximal and distal halves of *nhr-31p*::3XFLAG::*nhr-31* were subcloned into pBlueScript SK+ to produce a 3,414 bp amplimer that extends 1,000 bp upstream of the translational start for C26B2.3a.1, contains a DYKDHD-DYKDHD-DYKDDDD (3×FLAG) motif, and is connected to the coding sequence for C26B2.3a.1 CDS and its 593 bp 3′ UTR via a linker. The sequence was verified, followed by microinjection of *nhr-31p*::3XFLAG::*nhr-31* and the co-marker *sur-5*::*GFP* into the gonad arm of *hlh-30(lf)* to generate *nhr-31(oe);hlh-30(lf)* worms. Please also see the list of strains in S3 Table.

### Experiments with *C*. *elegans* harboring extrachromosomal arrays

All *C*. *elegans* lines expressing extrachromosomal arrays as above were generated with coinjection markers as indicated in S3 Table. Variable transmission rates between 30%–50% were noted. Only worms expressing the coinjection markers were analyzed for the endpoints reported with these strains.

### Preparation of CeMM

2× CeMM medium was prepared as described, using D-glucose as the energy source [31]. The vitamin, nucleic acids, trace metals, heme and salt solutions were prepared and stored at 4 °C the day prior to compounding the final solution. The β-sitosterol and amino acids were made on the day of compounding. The nonessential amino acids and essential amino acids were made as one solution to reduce loss of compounds. Glassware was soaked overnight in mild detergent, thoroughly rinsed with distilled water, soaked in 0.5M HCl overnight, rinsed again with distilled water, dried, and autoclaved before use. All transfers between glassware were done quantitatively with water to rinse the container of residue. After filtration with a 0.22-μm cellulose acetate filter, 2× CeMM was wrapped in aluminum foil to protect from light and stored at 4 °C. Water purified with the Milli-Q Synthesis A10 system (MilliporeSigma, Burlington, MA, USA) was used to make all solutions. CeMM minimal was prepared by combining vitamins, growth factors, salts, trace metals, and heme. It lacks the four major classes of macronutrients, namely amino acids, carbohydrates, lipids, and nucleic acids. For some experiments, D-glucose (Sigma-Aldrich, G8270; St. Louis, MO, USA), L-glucose (Sigma-Aldrich, G5500), or 2′-deoxy-D-glucose (Sigma-Aldrich, D8375) were added to CeMM Minimal Medium at equimolar concentrations along with a lipid component comprised of β-sitosterol or cholesterol dissolved in Tween 80 (Sigma-Aldrich, P8074).

### Conjugation of free fatty acids to bovine serum albumin

Free fatty acids were conjugated to fatty-acid–free bovine serum albumin (BSA, Seracare, 1900–0011; Milford, MA, USA) as previously described [66]. A 20% BSA solution was made in sterile phosphate-buffered saline (PBS), filtered with a 0.22-μm cellulose acetate filter, and stored at 4 °C. 20 mM palmitic acid stock was prepared with mixing 5 mL of water, 50 μL of 1 M NaOH, and 26.4 mg of palmitic acid (Cayman Chemical, 10011298; Ann Arbor, MI, USA) in a 15-mL Falcon tube, vortexed immediately, and placed in a 72 °C water bath. To solubilize the saturated fatty acids, 20 μL of 1 M NaOH was added every 10 minutes, immediately vortexed, and returned to the 72 °C water bath. Addition of 110 μL of 1 N NaOH was sufficient to solubilize palmitic acid. To conjugate palmitic acid to BSA at a molar ratio of 4:1, 0.6 mL of 20 mM palmitic acid was added to 1.98 mL of 37 °C 20% BSA and immediately mixed by pipetting. To make a 1 mM final concentration of palmitic acid in 1× CeMM, 2.15 mL palmitic acid:BSA conjugate was added to 5 mL 2× CeMM and 2.85 mL water in a 15-mL Falcon tube and immediately mixed by pipetting. CeMM containing conjugated palmitic acid was stable at 20 °C. A similar approach was used to make 20 mM stock solutions of stearic acid (Cayman Chemical, 10006627), linoleic acid (Cayman Chemical, 90150), and oleic acid (Sigma, O1383). Free fatty acid–BSA complexes were prepared at a molar ratio of 4:1 by adding 20 mM stock solutions to 20% BSA. Since linoleic acid is supplied as a solution in ethanol, each 20 mM stock solution of lipid was supplemented to contain an equal percentage of ethanol (1.88%), which was also employed as a diluent. The solutions were mixed by pipetting, then immediately added to 1× CeMM, in which the final concentration of each lipid was 1 mM.

### Feeding with fluorescent-labeled microspheres and GFP-expressing *OP50*

Fluoresbrite YG Carboxylate (0.5-μm diameter) microspheres (Polysciences, Inc., 15700–10; Warrington, PA, USA) were mixed with *E*. *coli OP50* in an equimolar ratio, spread on the NGM dish and allowed to dry, and placed in a 20 °C incubator for one hour. Bacterial concentration was estimated using the method described [67]. The final concentration of microspheres was 1 × 10^9^ per dish. To measure intact bacteria in the intestine, we seeded NGM plates with a strain of *E*. *coli OP50* expressing GFP obtained from the Caenorhabditis Genome Center (CGC), as previously described [26]. Worms were starved for 33 hours, placed on prepared dishes for duration of time as indicated, washed with M9, and imaged with fluorescent microscopy.

### Assays with proton pump inhibitors

Worms were starved 33 hours, then placed in CeMM containing 25 μM Baf-A1 (LC Laboratories, B-1080; Woburn, MA, USA), 400 nM concanamycin A (Sigma C9705), or respective controls 0.34% DMSO or 1.2% DMSO for 15 hours, followed by transfer to *E*. *coli OP50*-seeded NGM dishes, and scored as Alive After Refeeding 48 hours later.

### Assay with rapamycin treatment

Rapamycin (LC Laboratories, R5000) was dissolved in 100% DMSO at 50 mg/mL and added to plate agar, M9, and CeMM at 200 μM. Controls contained an equivalent DMSO concentration. Eggs were placed on NGM dishes containing rapamycin and *OP50*. Worms were grown to L4 stage, washed seven times in M9, adjusted to a density of one worm per μL, then placed in M9 containing 200 μM rapamycin. After 36 hours at 20 °C, worms were washed in M9, then transferred to CeMM containing 200 μM rapamycin for 15 hours. Then, worms were washed in M9 and placed on NGM dishes containing *OP50* and 200 μM rapamycin for 72 hours, where the number alive after refeeding was scored.

### LysoTracker Red staining

For all experiments, LysoTracker Red DND-99 (Invitrogen, L7528; Carlsbad, CA, USA) was used at a final concentration of 1 μM, and worms were incubated at 20 °C. For collecting fed worms, gravid adults were bleached, and eggs were washed and resuspended in 1 mL M9 containing LysoTracker. A glass Pasteur pipet was used to transfer eggs from M9 to an NGM dish containing dried *E*. *coli OP50* that was grown overnight with LysoTracker. 18 hours after bleaching, worms were washed twice in fresh M9 (without LysoTracker) and imaged. For collecting starved worms, worms were bleached and placed in M9 containing LysoTracker and starved for 33 hours, followed by two washes with fresh M9 (without LysoTracker) and imaged. For collecting refed worms, worms were starved for 33 hours in M9 with LysoTracker as described, then refed with CeMM containing LysoTracker for 15 hours, washed twice with fresh M9 (without LysoTracker), and imaged. To allow for operator viewing, all fluorescent images were configured in ImageJ to have a maximum brightness and contrast of 50. Worms were outlined and measured for area and integrated density. The corrected total worm fluorescence (CTWF) was calculated for each worm by “CTWF = Integrated density − (Area × mean fluorescence of control),” where the control was taken from worms not exposed to LysoTracker.

### Immunoblotting

To generate *C*. *elegans* protein extracts, we washed worm pellets (containing approximately 300,000 worms/sample) three times with M9 and homogenized in 200 μL of ice-cold buffer containing 50 mM Tris HCl (pH 7.4), 2.5 mM EDTA, 25 mM NaCl, 0.2% NP 40, 10 mM EGTA, 20 mM NaFl, 25 mM Na4O7P2, 2 mM Na3VO4 with protease and phosphatase inhibitor (Halt Protease and Phosphatase Inhibitor, Single-Use Cocktail, Thermo Fisher Scientific, cat# 78442; Waltham, MA, USA). Mechanical action was used to break apart worm tissue. Samples were then centrifuged 200 × *g* for 20 minutes at 4 °C, and supernatant was transferred to a fresh 1.7-mL Eppendorf tube waiting on ice. Protein was measured with the Bradford assay performed on a Bio-Rad Smart Spec Plus Spectrophotometer (Bio-Rad, Hercules, CA, USA). 10 μg of protein was loaded on SDS-PAGE gels, and immunoblotting was performed as previously described [68]. Antibodies employed were as follows: anti-RFP (MBL International, PM005, at 1:1,000 dilution; Woburn, MA, USA), 1:100 dilution of anti-LMP-1 (Developmental Studies Hybridoma Bank, University of Iowa, LMP-1, at 1:200 dilution; Iowa City, IA, USA), anti-FLAG, anti-GAPDH (EMD Millipore, MAB374, at 1:500 dilution), anti-HISTONE H3 (Abcam, ab1791, at 1:2,000 dilution; Cambridge, UK), and anti-β-ACTIN (Sigma-Aldrich, A8266, at 1:2,000 dilution). Secondary antibodies employed were as follows: Hrp-linked anti-rabbit IgG (Cell Signaling Technology, 7074S, at 1:5,000 dilution; Danvers, MA, USA) and Hrp-linked anti-mouse IgG (Cell Signaling, 7076S, at 1:5,000 dilution). ImageJ software was employed for quantitative analysis. Protein abundance was normalized to GAPDH, HISTONE H3, or β-ACTIN protein expression and reported as fold change versus control.

### ATP assay

L1 stage worms (containing approximately 250,000 worms/sample) were harvested and washed three times in M9 buffer, then followed by three freeze/thaw cycles and boiled for 15 minutes. The samples were spun at 11,000g for 10 minutes at 4 °C. The supernatant was collected and measured for ATP activity by the ATP determination kit (Thermo Fisher Scientific, A22066) and further normalized to the amount of dsDNA with Quant iT PicoGreen dsDNA reagent (Thermo Fisher Scientific, P11496) as described [69].

### Methods for RNAi delivery

To deliver dsRNA to worms by feeding, gravid wild-type and *hlh-30(lf)* adults were bleached, and eggs were placed on NGM dishes containing 5 mM IPTG and 25 μg/ml carbenicillin. An *HT-115(DE3)* bacterial colony containing L4440 or *bec-1* plasmid [70] was inoculated in LB broth containing 25 μg/mL carbenicillin, and grown for eight hours in a 37 °C shaker. The bacteria were plated on the NGM dish containing IPTG and carbenicillin an hour prior to addition of the worms. The bacterial solution was permitted to dry on the surface of the dish. Worms were added to NGM dishes containing IPTG, carbenicillin, and freshly plated bacteria. To starve worms, we washed L4 worms off dishes with M9, washed the worms seven times, then resuspended them at a density of one worm per μL in a 15 mL tube containing M9. The tubes were laid flat on a horizontal shaker at 20 °C for 36 hours, then the worms were incubated in CeMM for 15 hours and placed on NGM dishes seeded with bacteria containing L4440 or *bec-1* plasmid. Worms were scored alive after refeeding 48 hours later. For RNAi to *nhr-31*, we employed dsRNA soaking. An *nhr-31* RNA construct was generated through PCR amplification of 1,569 bp from GE Dharmacon *C*. *elegans* clone C26B2.3 ORF. To check for potential cross interference, defined by greater than 80% identity over 200 bp, a nucleotide Blast search was performed using WormBase. There were no matches greater than 200 bp except for exon sequences found in *nhr-31*. The amplimer was subcloned into the multiple cloning site of L4440 at the *HindIII* and *NotI* restriction site, between the T7 RNA polymerase binding sites, which was verified with sequencing. The clone for expressing RNAi targeting *vha-12* was obtained from the Ahringer RNAi library. To generate dsRNA from these clones, a PCR produced a linear template of dsDNA that included the subcloned *nhr-31* fragment or the *vha-12* fragment and the flanking T7 RNA polymerase binding sites. The product of the DNA polymerase reaction was purified using the Monarch PCR and DNA Cleanup Kit (New England BioLabs [NEB], T10305; Ipswich, MA, USA). Subsequently, in vitro transcription was performed with 500 ng of purified dsDNA using the HiScribe T7 RNA Polymerase Kit (NEB, E2040S). The product of the RNA polymerase reaction was purified using the RNeasy MinElute Kit (Qiagen, 74204; Hilden, Germany). The starvation and refeeding assay for L1 stage larvae was performed as described above in the presence of 1 μg of L4440, *nhr-31*, or *vha-12* dsRNA.

### Microscopy

LysoTracker Red DND-26 (Invitrogen, L7528) was diluted in M9 and dispensed on NGM dishes, M9, or CeMM to yield a final concentration of 1 μM. Worms were cultured on these dishes or liquid solutions in the dark and transferred to NGM dishes or fresh liquid without dye for 30 min. The animals were paralyzed with 25 mM levamisole (Sigma-Aldrich, 196142) in M9, mounted on 3% agarose pads on microscope slides, and imaged with an Olympus FV1200 Confocal Microscope (Olympus, Tokyo, Japan).

### Transmission electron microscopy

Samples containing L1 worms in fed, starved, CeMM, and refed *OP50* conditions were prepared and subjected to transmission electron microscopy analyses as described [71]. After three washes in M9, worms were resuspended in M9 containing 25 mM levamisole and 20% BSA. Samples were processed to cut thin sections onto standard 200 mesh grids and poststained with uranyl acetate and lead citrate. Images were taken with a JEOL JEM-1400Plus 120kV Transmission Electron Microscope (JEOL, Tokyo, Japan) equipped with an AMT XR111 high-speed 4,000 × 2,000 pixel phosphor-scintillated 12-bit CCD camera.

### Quantitative PCR analysis

Worms were collected in Eppendorf tubes and washed three times in M9. We added 400 μL of TRIzol (Thermo Fisher Scientific, 15596026) to the worm pellet (containing approximately 50,000 worms/sample), and the tube was frozen immediately in liquid nitrogen and stored at −80 °C. Following three cycles of freeze–thaw, 200 μL of TRIzol was added with incubation at room temperature for five minutes. Thereafter, 140 μL of chloroform (Sigma-Aldrich, 319988) was added, and the samples were shaken by hand to mix for 60 seconds and incubated at room temperature for two minutes, followed by centrifugation at 12,000 × *g* for 15 minutes at 4 °C. The aqueous phase and an equal volume of 70% ethanol were added to a fresh 1.7-mL tube. Nucleic acids were isolated with the RNeasy Mini Kit (Qiagen, 74104) following manufacturer’s instructions. cDNA was generated with the iScript Reverse Transcription Supermix for RT-qPCR (Bio-Rad, 172–5121). cDNA was mixed with Syber Green Master Mix (Bio-Rad, 172–5124) as recommended by the manufacturer and analyzed on an Applied Biosystems 7500 Real Time PCR System. Cycle threshold (Ct) values were normalized to the control gene *ama-1* or *act-1*. See S6 Table for a list of primers employed.

### RNAseq profiling

Transcriptomics analyses were carried out using RNAseq. An Illumina HiSeq 2500 (San Diego, CA, USA) was used to obtain single-ended 50-nucleotide reads. Two samples were prepared for wild-type and *hlh-30(lf)* worms (containing approximately 150,000 worms/sample) at L1 stage under fed, starved, and CeMM-refed conditions (at 3 and 15 hours), necessitating the multiplexing of multiple libraries into two lanes for sequencing. Total reads per individual sample averaged 39.3 million, with 99% alignment to the *C*. *elegans* genome (average across samples) to the Ensembl release 76 assembly with STAR version 2.0.4b. Sequencing performance was assessed for total number of aligned reads, total number of uniquely aligned reads, genes and transcripts detected, ribosomal fraction, known junction saturation, and read distribution over known gene models with RSeQC version 2.3. Gene counts were derived from the number of uniquely aligned unambiguous reads by Subread:featureCount version 1.4.5. All gene-level counts were then imported into the R/Bioconductor package EdgeR, and TMM normalization size factors were calculated to adjust for samples for differences in library size. Genes not expressed in any samples (i.e., with zero counts across all samples) were excluded from further analysis. The TMM size factors and the matrix of counts were then imported into R/Bioconductor package Limma, and weighted likelihoods based on the observed mean-variance relationship of every gene were then calculated for all samples with the Voom function. Overall similarity and difference of the samples was assessed with a Spearman correlation matrix and multidimensional scaling plots (see S24 Fig). Read modeling was assessed with plots of residual standard deviation of every gene to their average log-count with a robustly fitted trend line of the residuals. Generalized linear models with robust dispersion estimates were then created to test for gene-level differential expression. Differentially expressed genes were then filtered for FDR adjusted *p*-values less than or equal to 0.05. To enhance biological interpretation, grouping of genes based on functional similarity was achieved using the R/Bioconductor packages GAGE and Pathview, together with generation of maps on known signaling and metabolism pathways curated by KEGG. To narrow down areas of interest, genes were obtained from all significantly regulated KEGG pathways: carbon metabolism #01200; phenylalanine metabolism #00360; tyrosine metabolism #00350; tryptophan metabolism #00380; glycine, serine, and threonine metabolism #00260; valine, leucine, and isoleucine degradation #00280; metabolism of xenobiotics #00980; arginine and proline metabolism #00330; glyoxylate metabolism #00630; glutathione metabolism #00480; ABC transporters #02010; ribosome biogenesis #03008; drug metabolism #00982 and #00983; fatty acid elongation #00062; biosynthesis of unsaturated fatty acids #01040; fatty acid degradation #00071; oxidative phosphorylation #00190; biosynthesis of amino acids #01230; retinol metabolism #00830; peroxisome #04146; lysosome #04142; and phagosome #04145. The CPM values for all 16 samples for each of these genes were further analyzed by unsupervised hierarchical clustering to generate heatmaps.

### Metabolomic profiling

Wild-type and *hlh-30(lf) C*. *elegans* samples (containing approximately 200,000 worms/sample) were starved for 33 hours in M9 media devoid of nutrients or starved 33 hours, then refed with a fully defined liquid diet (CeMM) for 15 hours; then worms were pelleted, snap frozen, and submitted for global metabolic profiling through Metabolon Inc. (Morrisville, NC, USA). Small biochemicals in the samples were extracted, separated by ultra-high-performance liquid chromatography, and identified in a semiquantitative manner by accurate mass tandem mass spectrometry as previously described [72]. A total of 265 compounds of known identity were analyzed. Following normalization to Bradford protein concentration, the raw data were median scaled with the median value across all samples set equal to 1.0 (to allow depiction of values across a wide range), and scaled intensity was calculated with log transformation and imputation of missing values, if any, with the minimum observed value for each compound. The *y*-axis for metabolite data reflects scaled intensity for each metabolite. ANOVA contrasts were used to identify biochemicals that differed significantly between experimental groups. An estimate of the false discovery rate (q-value) was calculated to take into account the multiple comparisons that normally occur in metabolomic-based studies and is reported alongside the *P*-value. Instrument variability was determined by calculating the median relative standard deviation (RSD) for the internal standards that were added to each sample prior to injection into the mass spectrometers. Overall process variability was determined by calculating the median RSD for all endogenous metabolites (i.e., noninstrument standards) present in 100% of the Client Matrix samples, which are technical replicates of pooled samples. Median RDS for instrument variability and total process variability were 2% and 8%, respectively. Hierarchical cluster analysis was performed with Array Studio (OmicSoft, Cary, NC, USA). Supervised statistical analysis was performed with random forest analysis, which classifies samples based on a consensus of decision trees built from the biochemical profiles [73]. For random forest classification model building, a random subset of the data with known class identity was selected to build the tree (“training set”), and then the remaining “out-of-bag” (OOB) data were used to test the model and obtain a class prediction for each sample. Iteration of the process thousands of times produced a forest, and the final classification of each sample was subjected to class prediction frequency for the OOB data over the whole forest [73].

### Targeted metabolomics analysis

Liquid chromatography–tandem mass spectrometry (LC-MS/MS) targeted metabolomics was performed for detecting linoleoyl-GPC, linoleoyl-lyso phosphatidyl choline (LPC)(18:3), α-FFA(18:3), γ-FFA(18:3), glucose, glucose-6-phosphate, maltose, maltotriose, citrate, α-ketoglutarate, malate, and fumarate. An internal standard solution for *C*. *elegans* (200 μg/mL ^13^C6-glucose-6-phosphate, 1 μg/mL d4-citric acid, 100 μg/mL ^13^C6-glucose, 100 μg/mL ^13^C12-maltose, 100 μg/mL ^13^C18-maltotriose, 200 μg/mL d2-alpha-ketoglutaric acid, 1 μg/mL d3-malic acid, 1 μg/mL ^13^C4-fumaric acid, 1 μg/mL d4-α-FFA[18:3], and 1 μg/mL of LPC[17:0]) in methanol–water (4:1) was prepared. The *C*. *elegans* samples were homogenized in internal standard solution (2 nL/worm) using an Omni Bead Ruptor (Omni International, Kennesaw, GA, USA) and centrifuged to obtain clear homogenate. For analysis of LPC(18:3), clear homogenate was directly injected into the LC-MS/MS system. For analysis of glucose, glucose-6-phosphate, maltose, maltotriose, citrate, α-ketoglutarate, malate, and fumarate, the clear homogenate (0.5 mL) was dried, the residue was partitioned between water (0.2 mL) and chloroform (0.75 mL), and the aqueous phase was injected into the LC-MS/MS system. For analysis of α-FFA(18:3) and γ-FFA(18:3), clear homogenate (0.2 mL) was dried and derivatized with N-(4-aminomethylphenyl)pyridium, and the derivatives were dissolved in methanol for LC-MS/MS analysis. Measurement of LPC(18:3), α-FFA(18:3), and γ-FFA(18:3) was performed on a Shimadzu 10A HPLC system (Kyoto, Japan) coupled to a Thermo Fisher Scientific TSQ Quantum Ultra triple quadrupole mass spectrometer, and data processing was conducted with Xcalibur (Thermo Fisher Scientific). The analysis of glucose, glucose-6-phosphate, maltose, maltotriose, citrate, α-ketoglutarate, malate, and fumarate was performed on a Shimadzu 20AD HPLC system coupled to an AB Sciex 4000QTRAP mass spectrometer (Sciex, Framingham, MA, USA), and data processing was conducted with Analyst 1.5.2. Quality control (QC) samples were prepared by pooling the aliquots of the study samples and were used to monitor the instrument stability. The QC samples were injected six times in the beginning to stabilize the instrument and were injected between every five study samples. The relative quantification was provided, and the data were reported as the peak area ratios of the analytes to the corresponding internal standards. All metabolites in QC samples show CV < 15%, except for maltose and maltriose, which were undetectable.

### Statistical methods

All results are expressed as mean ± standard error of the mean (SEM). Each “replicate” indicates a biological replicate performed at a different time along with the controls (which were tested at the same time as that replicate). Statistical differences were assessed with the unpaired Student *t* test for two independent groups and one-way ANOVA or two-way ANOVA for comparing results across multiple groups for one or two variables, respectively, with GraphPad Prism software. Bonferroni’s post hoc test was employed after ANOVA for testing all pairwise comparisons. A 2-tailed value of *P* < 0.05 was considered statistically significant.

## Supporting information

S1 Fig *hlh-30* was necessary for starved L1 worms to survive during starvation and refeeding. Wild-type and *hlh-30(lf)* worms were analyzed after variable periods of starvation for Alive after Starvation (A) and Alive after Refeeding (B) as described in the legend for Fig 1A. *N* = 5 biological replicates of approximately 50 worms/time point; values indicate mean ± SEM. *P < 0.05 by post hoc test following two-way ANOVA. The data are similar to Fig 1, but the extended durations of starvation reveal the sensitivity of wild-type worms. Raw data are located in S2 Data. *hlh-30*, basic helix–loop–helix transcription factor 30; *hlh-30(lf)*, loss-of-function *tm1978* mutation *hlh-30*; L1, first larval stage; SEM, standard error of the mean.
(TIF)

S2 Fig *hlh-30(lf)* lethality was still observed when starved L1 larvae were fed UV-killed *E*. *coli*, live *Comamonas*, or live *E*. *coli* in liquid medium. Wild-type and *hlh-30(lf)* worms were analyzed after refeeding for 48 hours following variable periods of starvation (Alive after Refeeding), as described in the legend for Fig 1A. Worms were refed with live *E*. *coli OP50* on NGM dishes (+ *OP50*), UV-killed *E*. *coli OP50* on NGM dishes (+ UV-killed *OP50*, panel A), live *Comamonas* bacteria on NGM dishes (+ *Comamonas*, panel B), or live *E*. *coli OP50* in S-basal liquid medium (+ *OP50* in S-medium, panel C). *N* = 50 worms/time point from one biological replicate. Raw data are located in S2 Data. *hlh-30*, basic helix–loop–helix transcription factor 30; *hlh-30(lf)*, loss-of-function *tm1978* mutation *hlh-30*; L1, first larval stage; NGM, nematode growth medium; S-medium, liquid medium containing concentrated *OP50*.
(TIF)

S3 Fig *hlh-30(lf)* mutant animals displayed L1 arrest during starvation and food ingestion following refeeding. (A) Transgenic worms with the integrated array *phlh-8*::*gfp*, which expresses GFP in the precursor M cell, were imaged by fluorescent microscopy. See S3 Table for the strain description. Scale bar is 20 μm. Representative fluorescence and DIC overlay images show a single green precursor M cell (white arrow) in starved L1 worms with a wild-type copy of *hlh-30* (*hlh-30*(+)) and *hlh-30(lf)*. If the *hlh-30(lf)* mutation abrogated the L1 arrest, then development would progress, resulting in M cell division and multiple green cells. Fifty worms per genotype were examined. (B) Pharyngeal pumping rate was scored using a dissecting microscope. *hlh-30(lf)* and wild-type L1 worms were scored after 33 hours of starvation and 1 hour of refeeding on NGM dishes with live *E*. *coli OP50*. Values are the average of 50 worms, and bars are standard error. The values were not significantly different (NS, *P* = 0.96). Raw data are located in S2 Data. (C) *hlh-30(lf)* and wild-type L1 worms were starved for 33 hours in M9 medium and cultured for 1 hour on NGM dishes with live *E*. *coli OP50* admixed with fluorescent microspheres (in a ratio of 1:1). Representative confocal images without (*left*) and with merged bright-field (DIC) images (*right*) show green fluorescence (white arrows) that indicates ingestion of the microspheres. 100 worms were examined in each group. Scale bar is 20 μm. (D) *hlh-30(lf)* and wild-type L1 worms were starved for 33 hours followed by 48 hours of refeeding on NGM dishes seeded with live GFP-expressing *E*. *coli OP50*. Representative confocal images with merged bright-field (DIC) and GFP fluorescence demonstrate lack of green bacteria inside the worm intestines. GFP-expressing bacteria were only visualized within and proximal to the pharynx (see arrows). 100 worms were examined in each group. Scale bar is 20 μm. DIC, differential interference contrast; GFP, green fluorescent protein; *hlh-30*, basic helix–loop–helix transcription factor 30; *hlh-30(lf)*, loss-of-function *tm1978* mutation *hlh-30*; L1, first larval stage; NGM, nematode growth medium; NS, not significant; *phlh-8*::*gfp*, transcriptional fusion of basic helix–loop–helix promoter and GFP integrated into the genome.
(TIF)

S4 Fig Regulation of autophagy and lysosomal transcripts in *hlh-30(lf)* mutant and wild-type animals under starvation and refeeding conditions. (A–N) mRNA abundance in au with values normalized to the control gene *ama-1* determined by qPCR for autophagy and lysosomal machinery genes (as named) in L1 stage wild-type and *hlh-30(lf)* animals in the fed state (fed), after starvation for 33 hours (starved), and after starvation for 33 hours followed by refeeding on *E*. *coli OP50* for 15 hours (OP50). *N* = 3 biological replicates/group. Bars indicate mean ± SEM. *P < 0.05 by post hoc test after one-way ANOVA. Raw data are located in S2 Data. *ama-1*, amanitin-binding subunit of RNA polymerase II; au, arbitrary unit; *hlh-30*, basic helix–loop–helix transcription factor 30; *hlh-30(lf)*, loss-of-function *tm1978* mutation *hlh-30*; L1, first larval stage; qPCR, quantitative PCR; SEM, standard error of the mean.
(TIF)

S5 Fig Starvation regulates multiple additional metabolic pathways in *hlh-30(lf)* worms compared with the wild type. (A) Venn diagram depicting significantly regulated (both up-regulated as well as down-regulated; see S2 Table) KEGG pathways in wild-type and *hlh-30(lf)* L1 worms that were fed or starved for 33 hours and subjected to RNAseq analysis. *N* = 2 biological replicates/group. (B) Unsupervised hierarchical clustering of significantly altered transcripts from A. Lists of genes identified under groups labeled A–E are presented in S2 Table. *hlh-30*, basic helix–loop–helix transcription factor 30; *hlh-30(lf)*, loss-of-function *tm1978* mutation *hlh-30*; KEGG, Kyoto Encyclopedia of Genes and Genomes; L1, first larval stage; RNAseq, RNA sequencing.
(TIF)

S6 Fig Overexpression of HLH-30::RFP via the *hlh-30* promoter resulted in nuclear localization in response to starvation. (A) *hlh-30* mRNA abundance determined by qPCR was analyzed in the wild type; *hlh-30(lf)*; *hlh-30(lf);amEx272*, a strain that overexpresses HLH-30::RFP (*hlh-30(oe)*); and *hlh-30(lf);amEx291*, a strain that overexpresses HLH-30::RFP with a mutation of the nuclear localization signal: *hlh-30(mNLS)(oe)*. All worms were analyzed at the L1 stage in the fed state. *N* = 3 biological replicates/group. Bars indicate mean ± SEM. *P < 0.05 by post hoc test after one-way ANOVA. (B) *hlh-30(oe)* and *hlh-30(mNLS)(oe)* animals were analyzed at the L1 stage in the fed state or after 33 hours of starvation. Representative images display DIC (*left*) and fluorescence to reveal HLH-30::RFP (*center*), and 2.5× magnified insets (*right*, outlined in the left and center images) to reveal starvation-induced nuclear localization of HLH-30::RFP (arrows). Scale bar is 20 μm. (C) Wild-type, *hlh-30(lf)*, *hlh-30(oe)*, and *hlh-30(mNLS)(oe)* worms were analyzed after 33 hours of starvation for “Alive after Starvation” (C) as described in the Fig 1 legend. *N* = 3 biological replicates/group of approximately 50 worms. Bars indicate mean ± SEM. *P < 0.05 by post hoc test after one-way ANOVA. Data for *hlh-30(oe)* and *hlh-30(mNLS)(oe)* are the analysis of one transgenic strain depicted in A. Eleven other independently derived *hlh-30(oe)* strains and one other independently derived *hlh-30(mNLS)(oe)* strain displayed similar results in these assays. Raw data are located in S2 Data. DIC, differential interference contrast; *hlh-30*, basic helix–loop–helix transcription factor 30; *hlh-30(mNLS)(oe)*, overexpressed HLH-30 with a mutant nuclear localization signal; *hlh-30(lf)*, loss-of-function *tm1978* mutation *hlh-30*; *hlh-30(oe)*, overexpressed HLH-30; qPCR, quantitative PCR; RPF, red fluorescent protein; SEM, standard error of the mean.
(TIF)

S7 Fig CeMM rescued starvation-induced lethality in *hlh-30(lf)* mutants for up to 4 days of starvation. *hlh-30(lf)* worms were starved for the indicated duration of time (on the *y*-axis), refed with *E*. *coli OP50* or CeMM, and analyzed for “Alive after Refeeding” as described in the legend for Fig 2A. *N* = 3 biological replicates with approximately 50 worms/time. Data are shown as mean ± SEM. *P < 0.05 versus *hlh-30(lf)* CeMM by post hoc test after two-way ANOVA. Raw data are located in S2 Data. CeMM, *C*. *elegans* maintenance medium; *hlh-30*, basic helix–loop–helix transcription factor 30; *hlh-30(lf)*, loss-of-function *tm1978* mutation *hlh-30*; SEM, standard error of the mean.
(TIF)

S8 Fig Metabolomic profiling points to a role for complex lipids and glucose in the rescue of starved *hlh-30(lf)* worms. (A) Hierarchical cluster analysis of metabolites measured in wild-type and *hlh-30(lf)* L1 stage worms subjected to 33 hours of starvation and analyzed immediately (St.) or analyzed after 15 hours in complete CeMM. *N* = 6 biological replicates/group with approximately 150,000 animals per replicate. (B) Random forest analysis of metabolites that accurately segregated *hlh-30(lf)* worms into starved or refed groups. See S4 Table for the entire list of measured metabolites. Glucose and lipid metabolites with common acyl group (linoleoyl) are labeled. CeMM, *C*. *elegans* maintenance medium; *hlh-30*, basic helix–loop–helix transcription factor 30; *hlh-30(lf)*, loss-of-function *tm1978* mutation *hlh-30*; L1, first larval stage.
(TIF)

S9 Fig Glucose and Tween 80 are the components of CeMM that are critical for rescue of starvation-induced lethality in *hlh-30(lf)* worms. (A–C) Wild-type and *hlh-30(lf)* worms were analyzed after 33 hours of starvation and 15 hours of exposure to complete or modified formulations of CeMM for “Alive after Refeeding” as described in the legend for Fig 2A. Individual nutrients (shown below) were added to CeMM minimal solution (which lacks glucose, β-sitosterol in Tween 80, amino acids, and nucleic acids) in panel A, CeMM minimal solution containing glucose in panel B, or CeMM minimal solution containing amino acids, glucose, and nucleic acids in panel C. Bars indicate mean (± SEM). *N* = 3 biological replicates/group for panels A and B, and *N* = 1 replicate for panel C with approximately 50 worms/condition. *P < 0.05 by post hoc test after two-way ANOVA for A and B. Raw data are located in S2 Data. CeMM, *C*. *elegans* maintenance medium; *hlh-30*, basic helix–loop–helix transcription factor 30; *hlh-30(lf)*, loss-of-function *tm1978* mutation *hlh-30*; SEM, standard error of the mean.
(TIF)

S10 Fig CeMM did not restore lipid metabolites to wild-type levels in starved *hlh-30(lf)* worms. Depiction of significantly altered lipid metabolism pathways, along with significantly regulated candidate metabolites in *hlh-30(lf)* and wild-type worms subjected to 33 hours of starvation in M9 medium (labeled as St.) followed by 15 hours of incubation in CeMM, depicted in heat maps and graphs. Data on some metabolites presented in Fig 3 are also shown here to conform to the pathway depicted. Bars indicate mean ± SEM. *N* = 6 biological replicates/group. *P < 0.05 by post hoc test after two-way ANOVA. *y*-axis values are scaled intensity for each metabolite. See S4 Table for detail. Raw data are located in S2 Data. CeMM, *C*. *elegans* maintenance medium; *hlh-30*, basic helix–loop–helix transcription factor 30; *hlh-30(lf)*, loss-of-function *tm1978* mutation *hlh-30*; SEM, standard error of the mean.
(TIF)

S11 Fig Metabolomic profiling reveals CeMM selectively increases metabolites in the glycolytic pathway and TCA in starved *hlh-30(lf)* worms. Depiction of various metabolic pathways that glucose is channeled into, along with significantly regulated metabolites in *hlh-30(lf)* and wild-type worms subjected to 33 hours of starvation in M9 medium (labeled as St.) followed by 15 hours of incubation in CeMM, depicted in heat maps and graphs. Data on some metabolites presented in Fig 3 are also shown here to conform to the pathway depicted. Bars indicate mean ± SEM. *N* = 6 biological replicates/group. *P < 0.05 by post hoc test after two-way ANOVA. *y*-axis values are scaled intensity for each metabolite. See S4 Table for detail. Raw data are located in S2 Data. CeMM, *C*. *elegans* maintenance medium; *hlh-30*, basic helix–loop–helix transcription factor 30; *hlh-30(lf)*, loss-of-function *tm1978* mutation *hlh-30*; SEM, standard error of the mean; TCA, tricarboxylic acid cycle.
(TIF)

S12 Fig CeMM refeeding of starved *hlh-30(lf)* worms resulted in increased abundance of multiple metabolites in the glycolytic pathway, TCA, and lipid metabolism as compared with *E*. *coli OP50* refeeding. (A–J) *hlh-30(lf)* worms were subjected to metabolomics analyses after 33 hours of starvation and 15 hours of exposure to CeMM or *E*. *coli OP50* to determine levels of α-ketoglutarate (A), fumarate (B), malate (C), citrate (D), glucose-6-phosphate (E), glucose (F), α-FFA (G), γ-FFA (H), LPC (18:3)-1 (I), and LPC (18:3)-2 (J). Data are presented as mean ± SEM. *N* = 4 biological replicates/group. *P < 0.05 by *t* test. Raw data are located in S2 Data. CeMM, *C*. *elegans* maintenance medium; FFA, linolenate; *hlh-30*, basic helix–loop–helix transcription factor 30; *hlh-30(lf)*, loss-of-function *tm1978* mutation *hlh-30*; LPC, lyso phosphatidyl choline; SEM, standard error of the mean; TCA, tricarboxylic acid cycle; α-FFA, α-linolenate; γ-FFA, γ-linolenate.
(TIF)

S13 Fig CeMM did not restore endocannabinoids and protein metabolites to wild-type levels in starved *hlh-30(lf)* worms. Depiction of significantly altered endocannabinoid and protein metabolism pathways, along with significantly regulated candidate metabolites in *hlh-30(lf)* and wild-type worms subjected to 33 hours of starvation in M9 medium (labeled as St.) followed by 15 hours of incubation in CeMM, depicted in heat maps and graphs. Bars indicate mean ± SEM. *N* = 6 biological replicates/group. *P < 0.05 by post hoc test after two-way ANOVA. *y*-axis values are scaled intensity for each metabolite. See S4 Table for detail. Raw data are located in S2 Data. CeMM, *C*. *elegans* maintenance medium; *hlh-30*, basic helix–loop–helix transcription factor 30; *hlh-30(lf)*, loss-of-function *tm1978* mutation *hlh-30*; SEM, standard error of the mean.
(TIF)

S14 Fig Metabolomic analysis reveals up-regulation of nucleic acid salvage pathways in CeMM-refed wild-type worms versus nucleic acid breakdown in CeMM-refed *hlh-30(lf)* worms. Depiction of significantly altered nucleic acid breakdown and salvage pathways, along with significantly regulated candidate metabolites in *hlh-30(lf)* and wild-type worms subjected to 33 hours of starvation in M9 medium (labeled as St.) followed by 15 hours of incubation in CeMM, depicted in heat maps and graphs. *N* = 6 biological replicates/group. *P < 0.05 by post hoc test after two-way ANOVA. Bars indicate mean ± SEM. *y*-axis values are scaled intensity for each metabolite. See S4 Table for detail. Raw data are located in S2 Data. CeMM, *C*. *elegans* maintenance medium; *hlh-30*, basic helix–loop–helix transcription factor 30; *hlh-30(lf)*, loss-of-function *tm1978* mutation *hlh-30*; SEM, standard error of the mean.
(TIF)

S15 Fig Worms deficient in *lipl-1*, *lipl-3*, and *lipl-4* did not display increased sensitivity to starvation stress. (A–D) mRNA abundance determined by qPCR in au for indicated genes in *hlh-30(lf)* and wild-type worms subjected to 33 hours of starvation or with 15 hours of refeeding with *E*. *coli OP50*. Fed worms are included as controls. Values were normalized to the control gene *ama-1*. Bars indicate mean ± SEM. *N* = 3 biological replicates/group. *P < 0.05 by post hoc test after two-way ANOVA. (E, F) Wild-type, *lipl-1(lf)*, *lipl-3(lf)*, and *lipl-4(lf)* worms were analyzed after variable periods of starvation for Alive after Starvation (E) and Alive after Refeeding (F) as described in the Fig 1 legend. Values are mean ± SEM. *N* = 3 biological replicates of approximately 25 worms/time point. *P < 0.05 versus wild type by post hoc test after two-way ANOVA. Raw data are located in S2 Data. *ama-1*, amanitin-binding subunit of RNA polymerase II; au, arbitrary unit; *hlh-30*, basic helix–loop–helix transcription factor 30; *hlh-30(lf)*, loss-of-function *tm1978* mutation *hlh-30*; *lipl-1(lf)*, loss-of-function mutation in lysosomal lipase 1; SEM, standard error of the mean.
(TIF)

S16 Fig *hlh-30* overexpression confers modest rescue in *lipl-2*–deficient worms. Wild-type, *lipl-2(lf)*, and *hlh-30(oe);lipl-2(lf)* worms were analyzed after variable periods of starvation for Alive after Refeeding as described in the legend for Fig 1A. Values are mean ± SEM. *N* = 3 biological replicates of approximately 50 worms/time point. *P < 0.05 for *hlh-30(oe);lipl-2(lf)* versus wild type and #*P* < 0.05 for *hlh-30(oe);lipl-2(lf)* versus *lipl-2(lf)* by post hoc test after two-way ANOVA. Raw data are located in S2 Data. *hlh-30*, basic helix–loop–helix transcription factor 30; *hlh-30(lf)*, loss-of-function *tm1978* mutation *hlh-30*; *hlh-30(oe)*, overexpressed HLH-30; *lipl-2(lf)*, loss-of-function mutation in lysosomal lipase 2; SEM, standard error of the mean.
(TIF)

S17 Fig Ultrastructural analyses reveal preserved intestinal brush border and mitochondria in starved *hlh-30(lf)* worms, with restoration of abundance of autophagic structures upon CeMM refeeding. Transmission electron microscopic analyses of L1 stage wild-type and *hlh-30(lf)* worms analyzed in the fed state, after 33 hours of starvation, and after 15 hours of exposure to complete CeMM. Representative images are from one experiment; we performed two biological replicates/group. Black arrowheads point to the intestinal brush border, large white arrows point to mitochondria, and thin white arrows point to autophagic structures (autophagosomes and autolysosomes). Representative of *N* = 2 independent trials per group. Scale bars = 500 nm. CeMM, *C*. *elegans* maintenance medium; *hlh-30*, basic helix–loop–helix transcription factor 30; *hlh-30(lf)*, loss-of-function *tm1978* mutation *hlh-30*; L1, first larval stage.
(TIF)

S18 Fig RNAi-mediated *bec-1* knockdown did not attenuate CeMM-induced rescue of starved *hlh-30(lf)* worms. (A) Wild-type and *hlh-30(lf)* worms were analyzed after 33 hours of starvation and 0 or 15 hours of exposure to complete CeMM for “Alive after Refeeding” as described in the legend for Fig 2A. Worms were exposed to feeding RNAi during the L1 to L4 stages to reduce the level of *bec-1* mRNA or L4440 as control and analyzed at the L4 stage. Bars indicate mean ± SEM. *N* = 3 biological replicates/group. *P < 0.05 by post hoc test after two-way ANOVA versus *hlh-30(lf)* with 0 hours of CeMM treatment (labeled *OP50*) or as indicated by horizontal line. (B) *bec-1* mRNA abundance in au with values normalized to the control gene *ama-1* determined by qPCR for fed wild-type and *hlh-30(lf)* worms. Bars indicate mean ± SEM. *P* = NS indicates that no statistically significant differences were noted by *t* test. (C) *bec-1* mRNA abundance was analyzed in wild-type and *hlh-30(lf)* L4 stage worms 72 hours after starvation. The control RNAi (L4440) value was set equal to 1.0 for both genotypes, and *bec-1* values were normalized to the expression of *ama-1*. Bars indicate mean ± SEM. *N* = 3 biological replicates/group. *P < 0.05 by post hoc test after one-way ANOVA. Raw data are located in S2 Data. *ama-1*, amanitin-binding subunit of RNA polymerase II; au, arbitrary unit; *bec-1*, *C*. *elegans* ortholog of human BECN1; CeMM, *C*. *elegans* maintenance medium; *hlh-30*, basic helix–loop–helix transcription factor 30; *hlh-30(lf)*, loss-of-function *tm1978* mutation *hlh-30*; L1, first larval stage; L4, fourth larval stage; NS, not significant; qPCR, quantitative PCR; RNAi, RNA interference; SEM, standard error of the mean.
(TIF)

S19 Fig Glucose and linoleic acid supplementation are sufficient to drive transcriptional up-regulation of *vha* genes in starved *hlh-30(lf)* worms. (A–L) mRNA abundance in au with values normalized to the control gene *ama-1* determined by qPCR for the indicated genes. *hlh-30(lf)* L1 worms were starved for 33 hours and then transferred to NGM dishes with *E*. *coli OP50* for 15 hours or fed CeMM minimal supplemented with glucose and linoleic acid (as described in Fig 2E) for 15 hours. Bars indicate mean ± SEM. *N* = 4–8 biological replicates/group. *P < 0.05 by *t* test. Raw data are located in S2 Data. *ama-1*, amanitin-binding subunit of RNA polymerase II; au, arbitrary unit CeMM, *C*. *elegans* maintenance medium; *hlh-30*, basic helix–loop–helix transcription factor 30; *hlh-30(lf)*, loss-of-function *tm1978* mutation *hlh-30*; L1, first larval stage; NGM, nematode growth medium; qPCR, quantitative PCR; SEM, standard error of the mean; *vha*, vacuolar H^+^-ATPase.
(TIF)

S20 Fig Differential effects of refeeding with *E*. *coli OP50* and CeMM on gene expression in starved *hlh-30(lf)* worms. (A–S) mRNA abundance in au with values normalized to the control gene *ama-1* determined by qPCR for the indicated genes. Wild-type and *hlh-30(lf)* L1 worms were starved for 33 hours and transferred to NGM dishes with *E*. *coli OP50* for 15 hours or fed CeMM for 15 hours. Bars indicate mean ± SEM. *N* = 3 biological replicates/group. *P < 0.05 by post hoc test after two-way ANOVA. Raw data are located in S2 Data. *ama-1*, amanitin-binding subunit of RNA polymerase II; au, arbitrary unit; CeMM, *C*. *elegans* maintenance medium; *hlh-30*, basic helix–loop–helix transcription factor 30; *hlh-30(lf)*, loss-of-function *tm1978* mutation *hlh-30*; L1, first larval stage; NGM, nematode growth medium; qPCR, quantitative PCR; SEM, standard error of the mean.
(TIF)

S21 Fig Overexpression of *nhr-31* was not sufficient to confer rescue in refed *hlh-30(lf)* worms. *nhr-31* overexpressing *hlh-30(lf)* worms (*nhr-31(oe);hlh-30(lf)*) have approximately 17-fold higher levels of *nhr-31* transcripts compared to the wild type. Animals were subjected to 33 hours of starvation followed by refeeding with CeMM minimal (see Fig 2D) supplemented with glucose or β-sitosterol with Tween 80. “Alive after Refeeding” was scored as described in the legend for Fig 2A. Bars indicate mean ± SEM. *N* = 3 biological replicates/group. No significant differences were observed between groups by two-way ANOVA. Raw data are located in S2 Data. CeMM, *C*. *elegans* maintenance medium; *hlh-30*, basic helix–loop–helix transcription factor 30; *hlh-30(lf)*, loss-of-function *tm1978* mutation *hlh-30*; *nhr-31*, nuclear hormone receptor 31; *nhr-31(oe)*, overexpressed *nhr-31*; SEM, standard error of the mean.
(TIF)

S22 Fig TOR pathway genes are significantly regulated by *hlh-30*. (A–H) mRNA abundance determined by qPCR in au with values normalized to the control gene *ama-1* for genes (as named) in L1 stage wild-type and *hlh-30(lf)* animals in the fed state (fed), after starvation for 33 hours (starved), and after starvation for 33 hours followed by refeeding on *E*. *coli OP50* (OP50) or CeMM (CeMM) for 15 hours. Bars indicate mean ± SEM. *N* = 6 biological replicates/group. *P < 0.05 by post hoc test after one-way ANOVA. Raw data are located in S2 Data. *ama-1*, amanitin-binding subunit of RNA polymerase II; au, arbitrary unit; CeMM, *C*. *elegans* maintenance medium; *hlh-30*, basic helix–loop–helix transcription factor 30; *hlh-30(lf)*, loss-of-function *tm1978* mutation *hlh-30*; L1, first larval stage; qPCR, quantitative PCR; SEM, standard error of the mean; TOR, target of rapamycin.
(TIF)

S23 Fig Analysis of *ragc-1* mRNA abundance in worms that overexpress *ragc-1*. *ragc-1* mRNA abundance in au with values normalized to the control gene *ama-1* was analyzed in fed L4 stage worms in the following groups: wild-type, *hlh-30(lf)*, and *hlh-30(lf);ragc-1(oe)* (*hlh-30(lf);amEx324* worms that overexpress *ragc-1* from an extrachromosomal array utilizing its endogenous promoter). Bars indicate mean ± SEM. *N* = 3–6 biological replicates/group. *P < 0.05 by post hoc test after one-way ANOVA. *ama-1*, amanitin-binding subunit of RNA polymerase II; au, arbitrary unit; *hlh-30*, basic helix–loop–helix transcription factor 30; *hlh-30(lf)*, loss-of-function *tm1978* mutation *hlh-30*; L4, fourth larval stage; *ragc-1*, the ortholog for mammalian RagC/D GTPases; *ragc-1(oe)*, overexpressed *ragc-1*; SEM, standard error of the mean.
(TIF)

S24 Fig RNAseq analysis on starved and refed *hlh-30(lf)* mutants and wild-type worms. (A, B) Spearman correlation matrix (A) and multidimensional scaling plot (B) depicting various RNA samples from wild-type and *hlh-30(lf)* L1 worms subjected to 33 hours of starvation, 33 hours of starvation followed by 3 or 15 hours of CeMM exposure (as in Fig 2B), or studied in the fed state and subjected to RNAseq. CeMM, *C*. *elegans* maintenance medium; *hlh-30*, basic helix–loop–helix transcription factor 30; *hlh-30(lf)*, loss-of-function *tm1978* mutation *hlh-30*; L1, first larval stage; RNAseq, RNA sequencing.
(TIF)

S1 Movie Wild-type worms (L1 stage) after 33 hours of starvation. L1, first larval stage.
(MP4)

S2 Movie Wild-type worms starved for 33 hours at the L1 stage and refed on *E*. *coli OP50* dishes for 48 hours. L1, first larval stage.
(MP4)

S3 Movie *hlh-30(lf)* worms (L1 stage) after 33 hours of starvation. *hlh-30*, basic helix–loop–helix transcription factor 30; *hlh-30(lf)*, loss-of-function *tm1978* mutation *hlh-30*; L1, first larval stage.
(MP4)

S4 Movie *hlh-30(lf)* worms starved for 33 hours at the L1 stage and refed on *E*. *coli OP50* dishes for 48 hours. *hlh-30*, basic helix–loop–helix transcription factor 30; *hlh-30(lf)*, loss-of-function *tm1978* mutation *hlh-30*; L1, first larval stage.
(MP4)

S5 Movie Wild-type worms starved for 33 hours at the L1 stage and refed with CeMM for 15 hours, followed by transfer to *E*. *coli OP50* dishes for 48 hours. CeMM, *C*. *elegans* maintenance medium; L1, first larval stage.
(MP4)

S6 Movie *hlh-30(lf)* worms starved for 33 hours at the L1 stage and refed with CeMM for 15 hours, followed by transfer to *E*. *coli OP50* dishes for 48 hours. All movies were obtained at identical magnification. CeMM, *C*. *elegans* maintenance medium; *hlh-30*, basic helix–loop–helix transcription factor 30; *hlh-30(lf)*, loss-of-function *tm1978* mutation *hlh-30*; L1, first larval stage.
(MP4)

S1 Table Description of mutant alleles used in this study. (PDF)

S2 Table Underlying processed data. (XLSX)

S3 Table List of *C*. *elegans* strains used in this study. (PDF)

S4 Table Underlying processed data. (XLSX)

S5 Table Underlying processed data. (XLSX)

S6 Table Primer description for qPCR analysis of indicated genes. qPCR, quantitative PCR.
(PDF)

S1 Data Raw data underlying this paper. (XLSX)

S2 Data Raw data underlying this paper. (XLSX)

## Abbreviations

AA: arachidonic acid
*ama-1*: amanitin-binding subunit of RNA polymerase II
*asp-1*: aspartyl protease 1
*atg*: autophagy yeast Atg homolog
*au*: arbitrary unit
*Baf-A1*: bafilomycin A1
*bec-1*: *C*. *elegans* ortholog of human BECN1
*bp*: base pair
*BSA*: bovine serum albumin
CeMM: *Caenorhabditis elegans* maintenance medium
CGC: Caenorhabditis Genetics Center
*cpr-1*: cysteine protease 1
Ct: cycle threshold
CTWF: corrected total worm fluorescence
*daf-15*: ortholog of mammalian raptor
*daf-16(lf)*: loss-of-function mutant for the sole member of the *C*. *elegans* FOXO Forkhead transcription factor family
DGLA: di-homo-gamma-linoleic acid
*elo-2*: fatty acid elongase
EPA: eicaso-pentaenoiec acid
*fat*: fatty acid desaturase
GAPDH: glyceraldehyde 3-phosphate dehydrogenase
GEF: guanine nucleotide exchange factor
GFP: green fluorescent protein
GPC: glycerolphosphocholine
*hlh-30(lf)*: loss-of-function *tm1978* mutation *hlh-30*
*hlh-30(mNLS)(oe)*: overexpressed HLH-30 with a mutant nuclear localization signal
*hlh-30(oe)*: overexpressed HLH-30
*hlh-30*: basic helix–loop–helix transcription factor 30
HNF-4α: hepatocyte nuclear factor 4α
INCL: infantile ceroid lipofuchsinosis
KEGG: Kyoto Encyclopedia of Genes and Genomes
L1: first larval stage
L2: second larval stage
L4: fourth larval stage
LB: Luria broth
LC-MS/MS: liquid chromatography–tandem mass spectrometry
*let-363*: ortholog of mammalian mTOR
*lgg-1*: ortholog of mammalian LC3, GABARAP and GATE-16 family
*lipl*: lysosomal lipase
*lipl-2*: lysosomal lipase 2
*lmp-1*: lysosome membrane protein 1
LPC: lyso phosphatidyl choline
LSD: lysosome storage disease
LYNUS: lysosomal nutrient sensing
MiT: Microphthalmia-associated transcription factor
MPS: mucopolysaccharidoses
mTORC1: mammalian TOR complex 1
NGM: nematode growth medium
NHR: nuclear hormone receptor
NS: not significant
oe: overexpression
OEA: oleyl ethanolamine
OOB: out of bag
PBS: phosphate-buffered saline
*phlh-8::gfp*: transcriptional fusion of basic helix–loop–helix promoter and GFP integrated into the genome
PPARα: peroxisome proliferator-activated receptor α
PUFA: polyunsaturated fatty acid
QC: quality control
*ragc-1(oe);hlh30(lf)*: *ragc-1* extrachromosomal array expressed in the *hlh-30(lf)* mutant
RAGC-1: the ortholog for mammalian RagC/D GTPases
RFP: red fluorescent protein
RNAseq: RNA sequencing
RSD: relative standard deviation
*rsks-1*: ortholog of mammalian S6 kinase
SEM: standard error of the mean
S-medium: liquid medium containing concentrated *OP50*
*sul*: sulfatase domain protein
TCA: tricarboxylic acid cycle
TFEB: Transcription Factor EB
*tm1978*: deletion allele obtained from the National BioResource Project lacks two exons
TOR: target of rapamycin
V-ATPase: vacuolar ATPase
*vha-12*: vacuolar H^+^-ATPase 12
*vit-1*: vitellogenin 1
*vps-11*: related to yeast vacuolar protein sorting factor 11
α-FFA: α-linolenate
γ-FFA: γ-linolenate
