# Supplementary figures and images for: Simple nutrients bypass the requirement for HLH-30 in coupling lysosomal nutrient sensing to survival
Source: PLoS Biol. 2019 May 14;17(5):e3000245. doi: 10.1371/journal.pbio.3000245 (PMC6516633; doi:10.1371/journal.pbio.3000245)

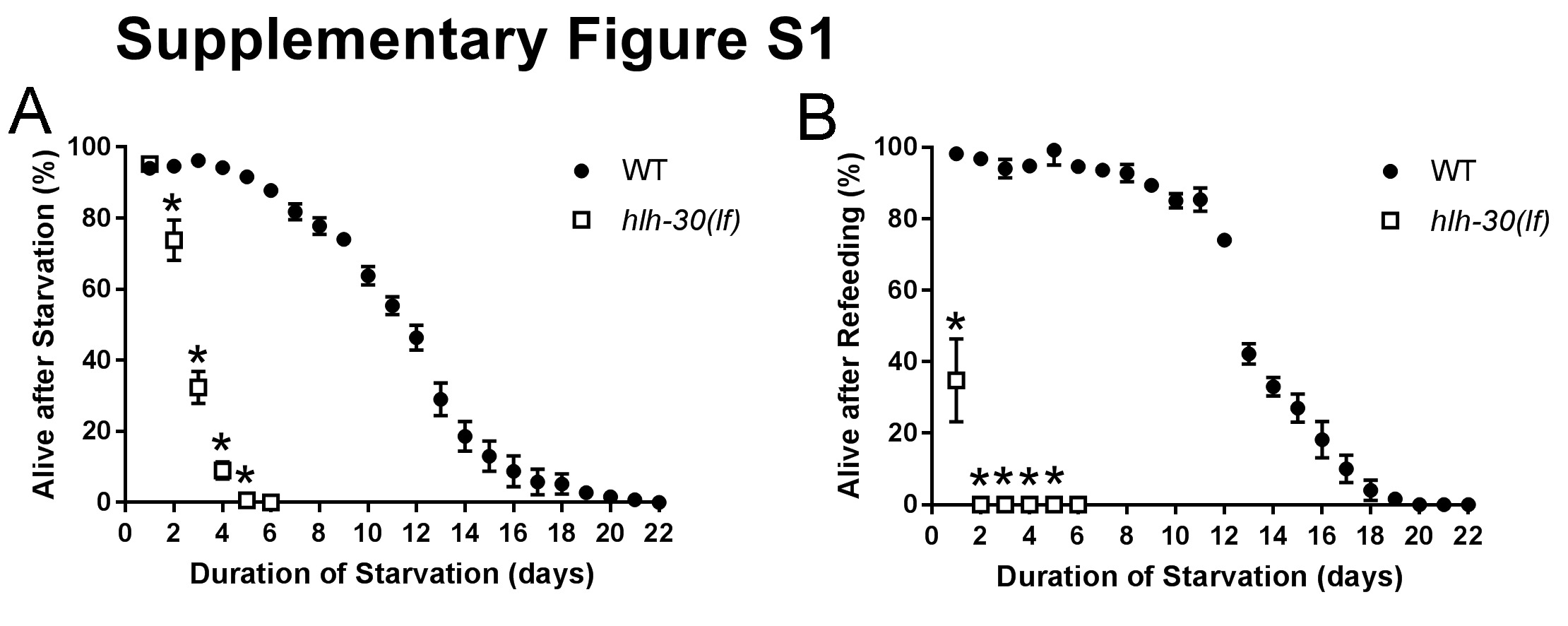

Supplement: S1 Fig — Wild-type and hlh-30(lf) worms were analyzed after variable periods of starvation for Alive after Starvation (A) and Alive after Refeeding (B) as described in the legend for Fig 1A. N = 5 biological replicates of approximately 50 worms/time point; values indicate mean ± SEM. *P < 0.05 by post hoc test following two-way ANOVA. The data are similar to Fig 1, but the extended durations of starvation reveal the sensitivity of wild-type worms. Raw data are located in S2 Data. hlh-30, basic helix–loop–helix transcription factor 30; hlh-30(lf), loss-of-function tm1978 mutation hlh-30; L1, first larval stage; SEM, standard error of the mean. (TIF) [file pbio.3000245.s001.tif]

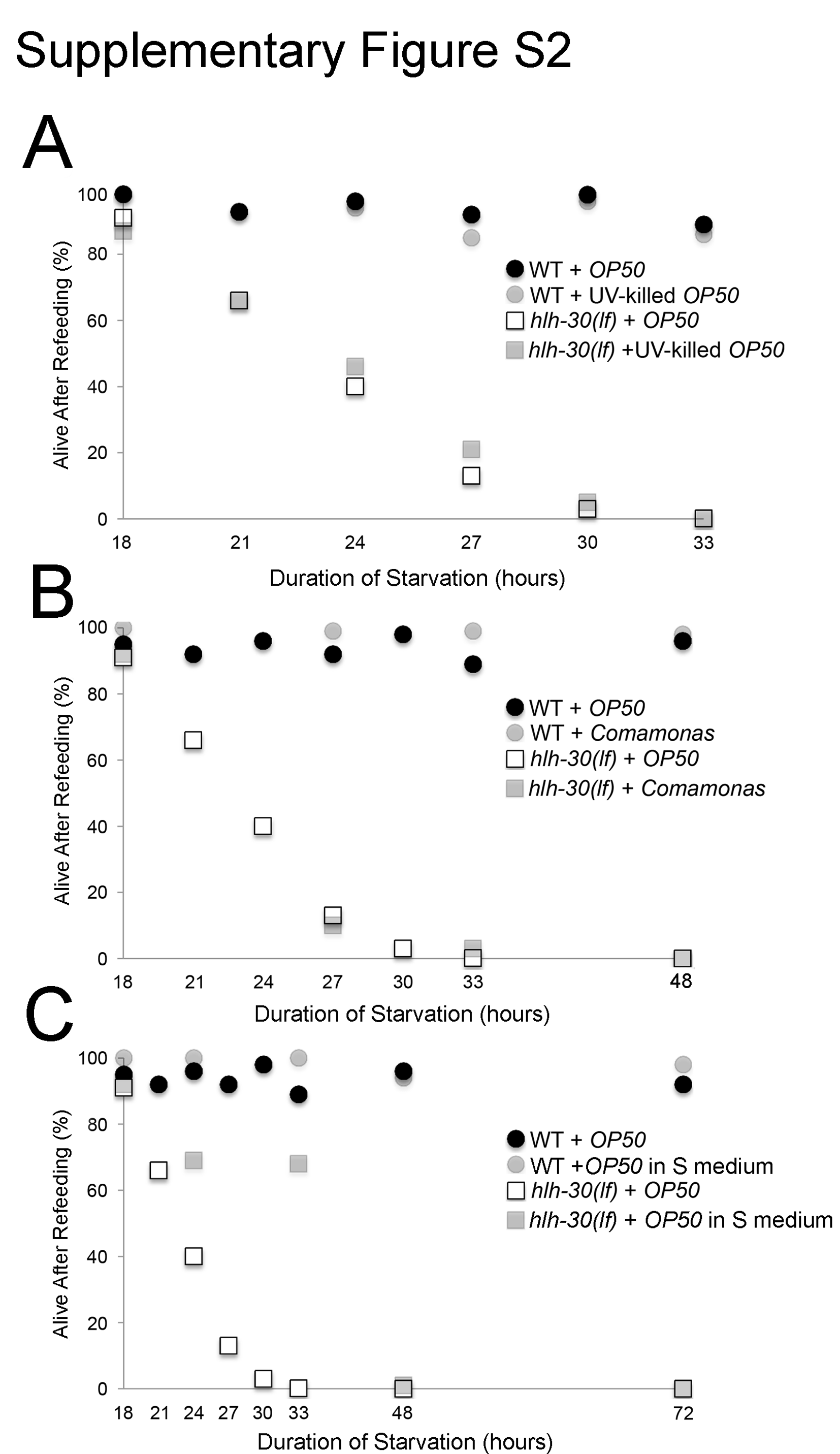

Supplement: S2 Fig — Wild-type and hlh-30(lf) worms were analyzed after refeeding for 48 hours following variable periods of starvation (Alive after Refeeding), as described in the legend for Fig 1A. Worms were refed with live E. coli OP50 on NGM dishes (+ OP50), UV-killed E. coli OP50 on NGM dishes (+ UV-killed OP50, panel A), live Comamonas bacteria on NGM dishes (+ Comamonas, panel B), or live E. coli OP50 in S-basal liquid medium (+ OP50 in S-medium, panel C). N = 50 worms/time point from one biological replicate. Raw data are located in S2 Data. hlh-30, basic helix–loop–helix transcription factor 30; hlh-30(lf), loss-of-function tm1978 mutation hlh-30; L1, first larval stage; NGM, nematode growth medium; S-medium, liquid medium containing concentrated OP50. (TIF) [file pbio.3000245.s002.tif]

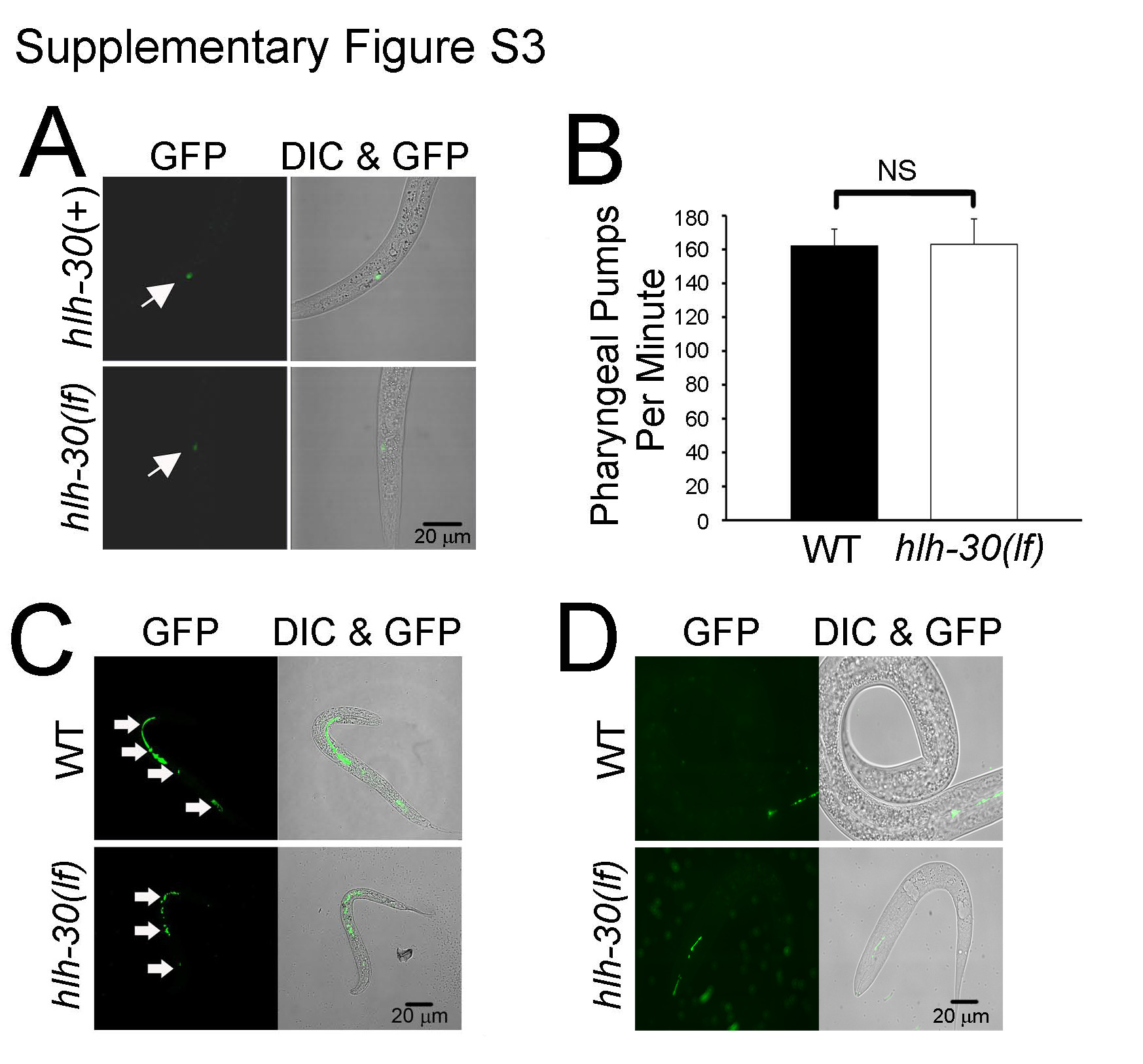

Supplement: S3 Fig — (A) Transgenic worms with the integrated array phlh-8::gfp, which expresses GFP in the precursor M cell, were imaged by fluorescent microscopy. See S3 Table for the strain description. Scale bar is 20 μm. Representative fluorescence and DIC overlay images show a single green precursor M cell (white arrow) in starved L1 worms with a wild-type copy of hlh-30 (hlh-30(+)) and hlh-30(lf). If the hlh-30(lf) mutation abrogated the L1 arrest, then development would progress, resulting in M cell division and multiple green cells. Fifty worms per genotype were examined. (B) Pharyngeal pumping rate was scored using a dissecting microscope. hlh-30(lf) and wild-type L1 worms were scored after 33 hours of starvation and 1 hour of refeeding on NGM dishes with live E. coli OP50. Values are the average of 50 worms, and bars are standard error. The values were not significantly different (NS, P = 0.96). Raw data are located in S2 Data. (C) hlh-30(lf) and wild-type L1 worms were starved for 33 hours in M9 medium and cultured for 1 hour on NGM dishes with live E. coli OP50 admixed with fluorescent microspheres (in a ratio of 1:1). Representative confocal images without (left) and with merged bright-field (DIC) images (right) show green fluorescence (white arrows) that indicates ingestion of the microspheres. 100 worms were examined in each group. Scale bar is 20 μm. (D) hlh-30(lf) and wild-type L1 worms were starved for 33 hours followed by 48 hours of refeeding on NGM dishes seeded with live GFP-expressing E. coli OP50. Representative confocal images with merged bright-field (DIC) and GFP fluorescence demonstrate lack of green bacteria inside the worm intestines. GFP-expressing bacteria were only visualized within and proximal to the pharynx (see arrows). 100 worms were examined in each group. Scale bar is 20 μm. DIC, differential interference contrast; GFP, green fluorescent protein; hlh-30, basic helix–loop–helix transcription factor 30; hlh-30(lf), loss-of-function tm1978 mutation [file pbio.3000245.s003.tif]

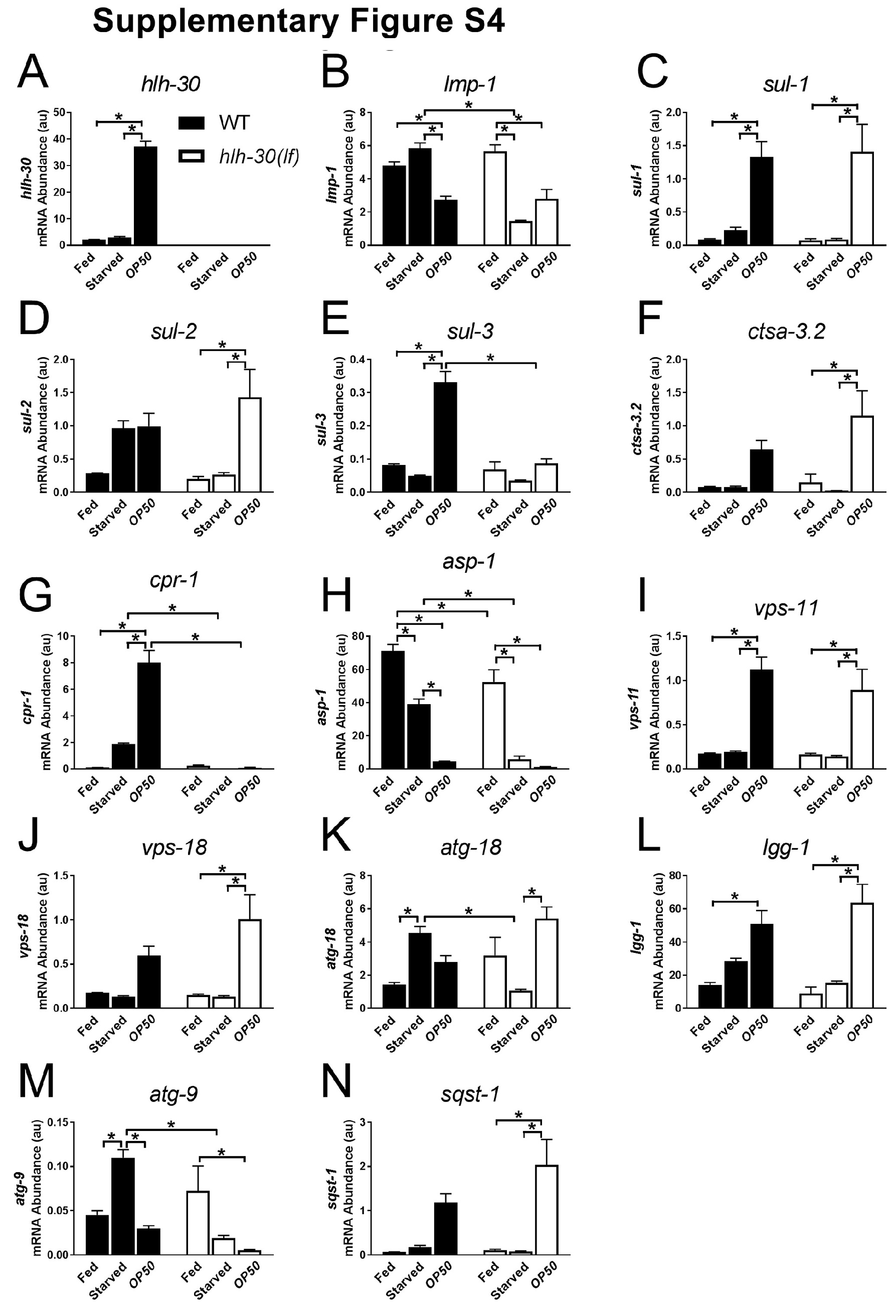

Supplement: S4 Fig — (A–N) mRNA abundance in au with values normalized to the control gene ama-1 determined by qPCR for autophagy and lysosomal machinery genes (as named) in L1 stage wild-type and hlh-30(lf) animals in the fed state (fed), after starvation for 33 hours (starved), and after starvation for 33 hours followed by refeeding on E. coli OP50 for 15 hours (OP50). N = 3 biological replicates/group. Bars indicate mean ± SEM. *P < 0.05 by post hoc test after one-way ANOVA. Raw data are located in S2 Data. ama-1, amanitin-binding subunit of RNA polymerase II; au, arbitrary unit; hlh-30, basic helix–loop–helix transcription factor 30; hlh-30(lf), loss-of-function tm1978 mutation hlh-30; L1, first larval stage; qPCR, quantitative PCR; SEM, standard error of the mean. (TIF) [file pbio.3000245.s004.tif]

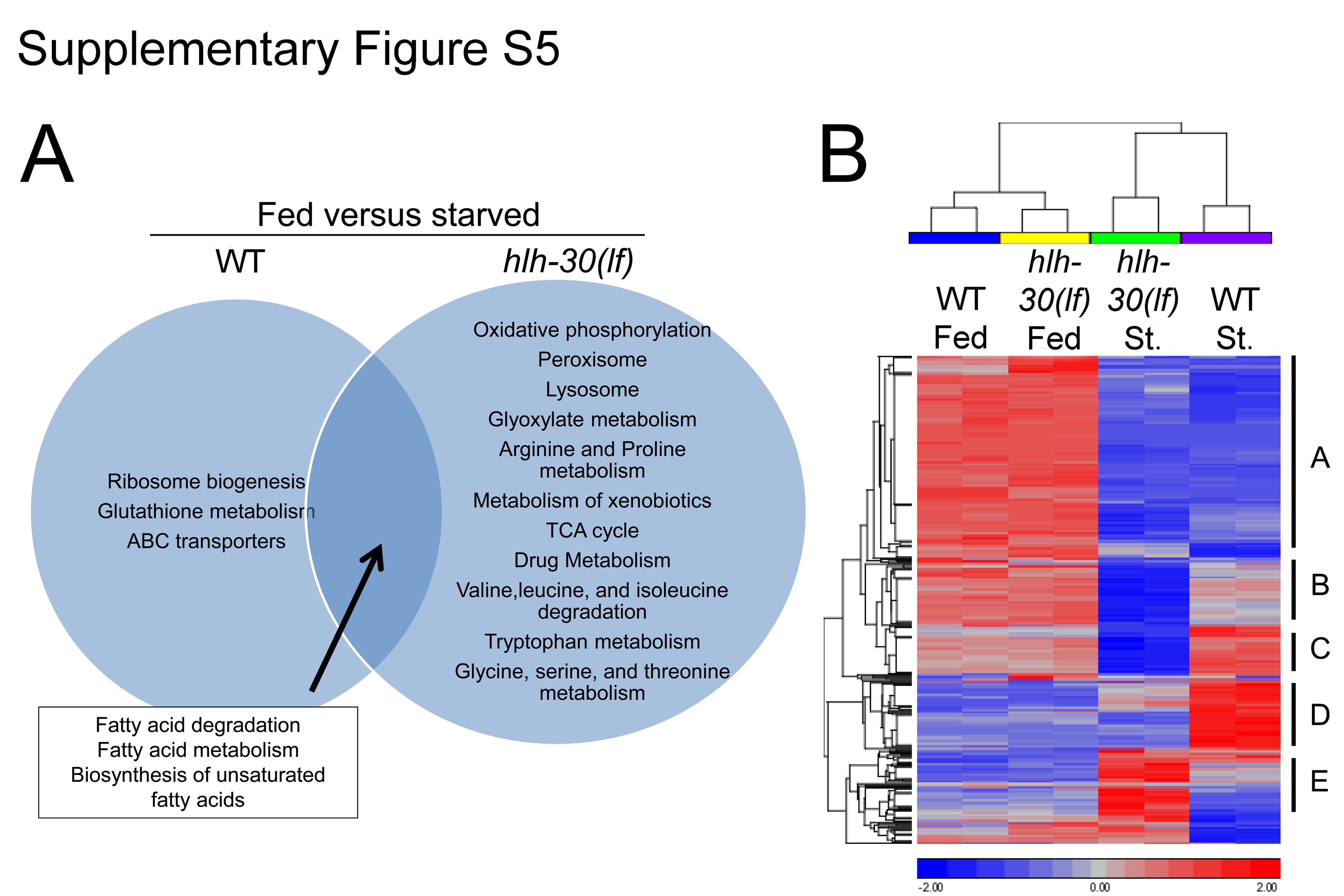

Supplement: S5 Fig — (A) Venn diagram depicting significantly regulated (both up-regulated as well as down-regulated; see S2 Table) KEGG pathways in wild-type and hlh-30(lf) L1 worms that were fed or starved for 33 hours and subjected to RNAseq analysis. N = 2 biological replicates/group. (B) Unsupervised hierarchical clustering of significantly altered transcripts from A. Lists of genes identified under groups labeled A–E are presented in S2 Table. hlh-30, basic helix–loop–helix transcription factor 30; hlh-30(lf), loss-of-function tm1978 mutation hlh-30; KEGG, Kyoto Encyclopedia of Genes and Genomes; L1, first larval stage; RNAseq, RNA sequencing. (TIF) [file pbio.3000245.s005.tif]

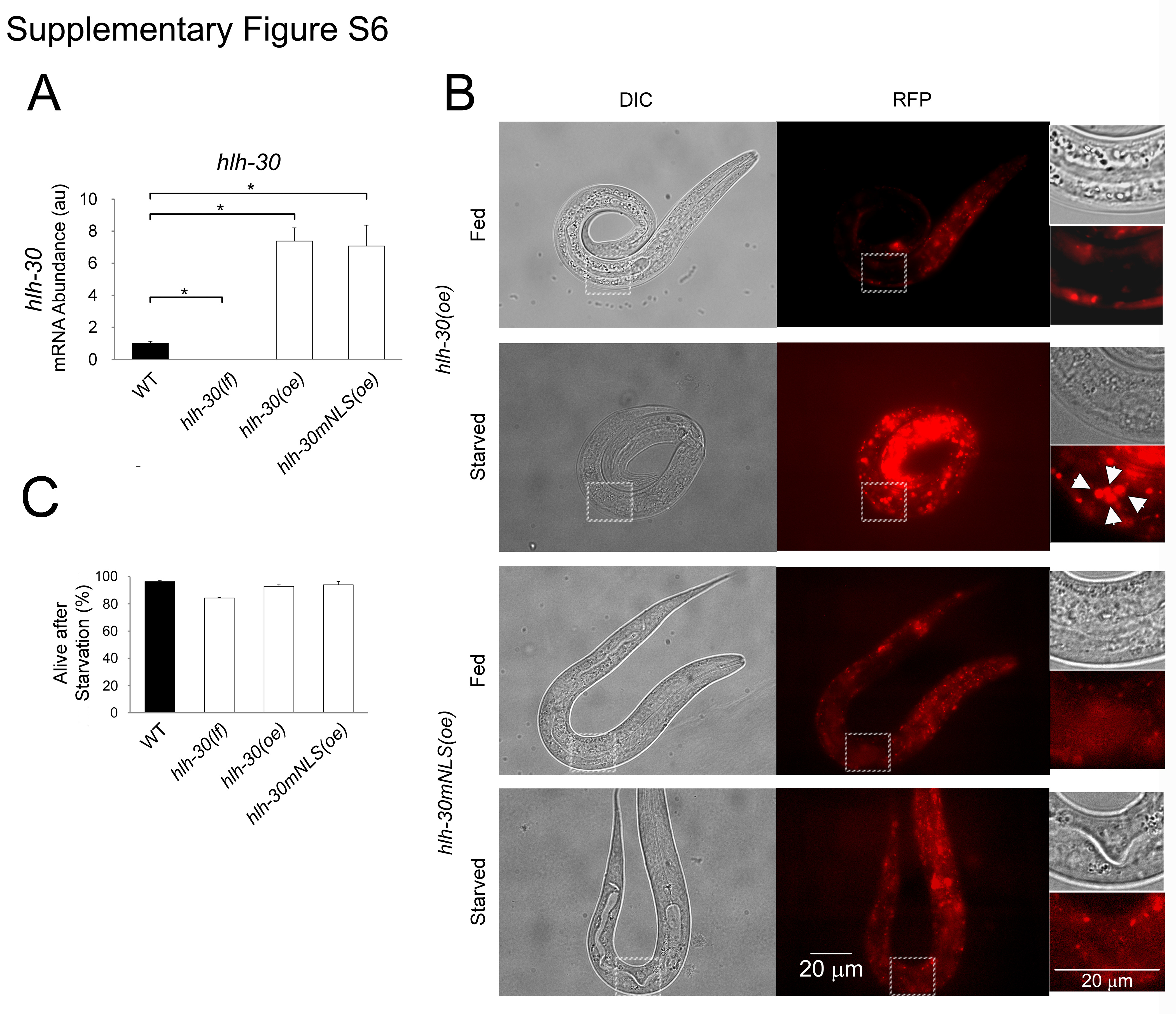

Supplement: S6 Fig — (A) hlh-30 mRNA abundance determined by qPCR was analyzed in the wild type; hlh-30(lf); hlh-30(lf);amEx272, a strain that overexpresses HLH-30::RFP (hlh-30(oe)); and hlh-30(lf);amEx291, a strain that overexpresses HLH-30::RFP with a mutation of the nuclear localization signal: hlh-30(mNLS)(oe). All worms were analyzed at the L1 stage in the fed state. N = 3 biological replicates/group. Bars indicate mean ± SEM. *P < 0.05 by post hoc test after one-way ANOVA. (B) hlh-30(oe) and hlh-30(mNLS)(oe) animals were analyzed at the L1 stage in the fed state or after 33 hours of starvation. Representative images display DIC (left) and fluorescence to reveal HLH-30::RFP (center), and 2.5× magnified insets (right, outlined in the left and center images) to reveal starvation-induced nuclear localization of HLH-30::RFP (arrows). Scale bar is 20 μm. (C) Wild-type, hlh-30(lf), hlh-30(oe), and hlh-30(mNLS)(oe) worms were analyzed after 33 hours of starvation for “Alive after Starvation” (C) as described in the Fig 1 legend. N = 3 biological replicates/group of approximately 50 worms. Bars indicate mean ± SEM. *P < 0.05 by post hoc test after one-way ANOVA. Data for hlh-30(oe) and hlh-30(mNLS)(oe) are the analysis of one transgenic strain depicted in A. Eleven other independently derived hlh-30(oe) strains and one other independently derived hlh-30(mNLS)(oe) strain displayed similar results in these assays. Raw data are located in S2 Data. DIC, differential interference contrast; hlh-30, basic helix–loop–helix transcription factor 30; hlh-30(mNLS)(oe), overexpressed HLH-30 with a mutant nuclear localization signal; hlh-30(lf), loss-of-function tm1978 mutation hlh-30; hlh-30(oe), overexpressed HLH-30; qPCR, quantitative PCR; RPF, red fluorescent protein; SEM, standard error of the mean. (TIF) [file pbio.3000245.s006.tif]

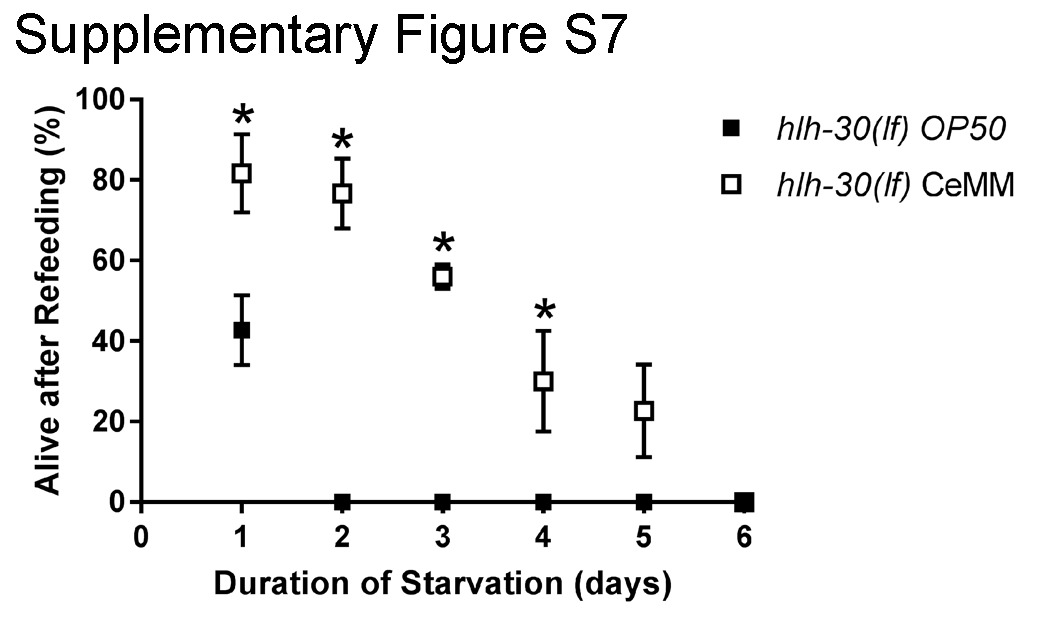

Supplement: S7 Fig — hlh-30(lf) worms were starved for the indicated duration of time (on the y-axis), refed with E. coli OP50 or CeMM, and analyzed for “Alive after Refeeding” as described in the legend for Fig 2A. N = 3 biological replicates with approximately 50 worms/time. Data are shown as mean ± SEM. *P < 0.05 versus hlh-30(lf) CeMM by post hoc test after two-way ANOVA. Raw data are located in S2 Data. CeMM, C. elegans maintenance medium; hlh-30, basic helix–loop–helix transcription factor 30; hlh-30(lf), loss-of-function tm1978 mutation hlh-30; SEM, standard error of the mean. (TIF) [file pbio.3000245.s007.tif]

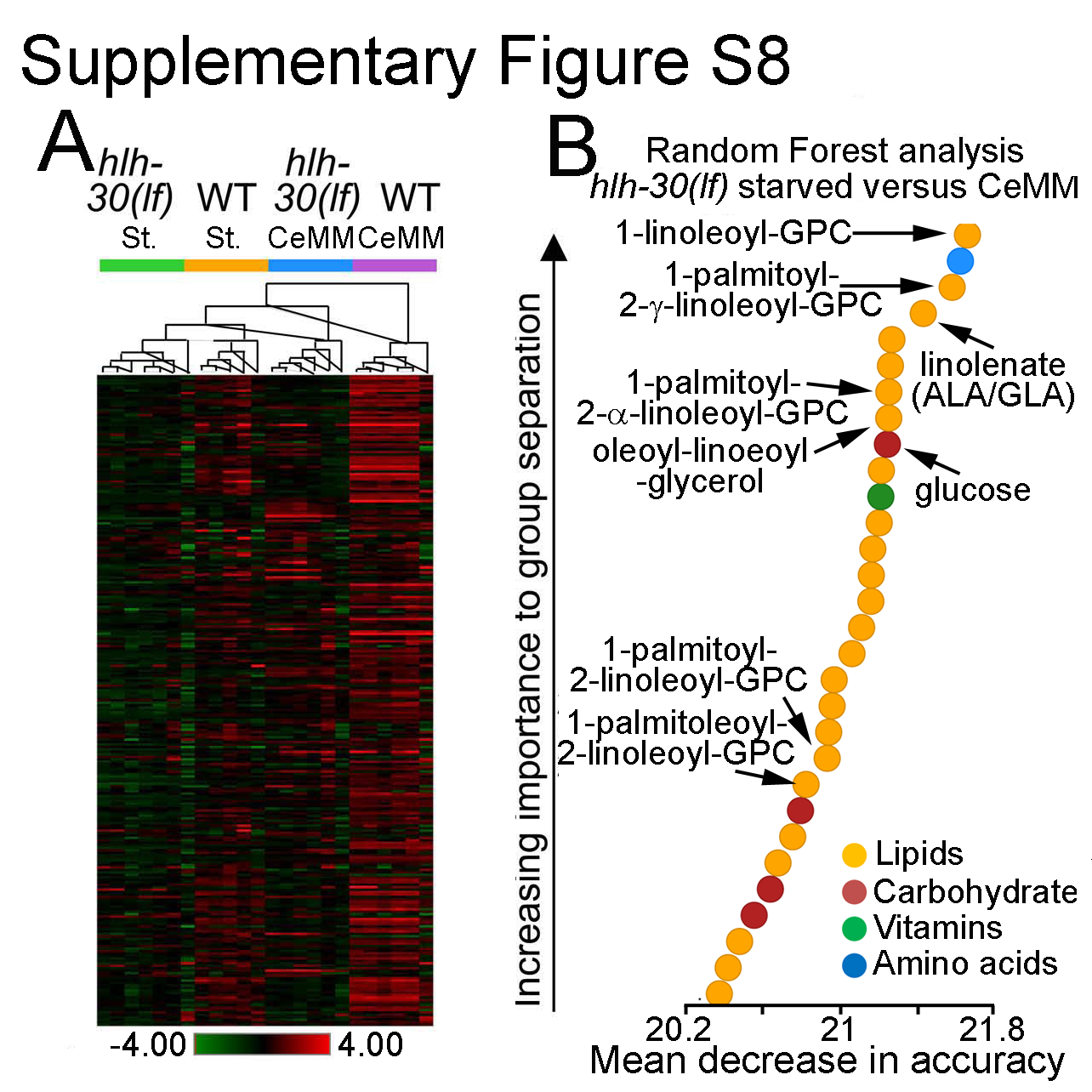

Supplement: S8 Fig — (A) Hierarchical cluster analysis of metabolites measured in wild-type and hlh-30(lf) L1 stage worms subjected to 33 hours of starvation and analyzed immediately (St.) or analyzed after 15 hours in complete CeMM. N = 6 biological replicates/group with approximately 150,000 animals per replicate. (B) Random forest analysis of metabolites that accurately segregated hlh-30(lf) worms into starved or refed groups. See S4 Table for the entire list of measured metabolites. Glucose and lipid metabolites with common acyl group (linoleoyl) are labeled. CeMM, C. elegans maintenance medium; hlh-30, basic helix–loop–helix transcription factor 30; hlh-30(lf), loss-of-function tm1978 mutation hlh-30; L1, first larval stage. (TIF) [file pbio.3000245.s008.tif]

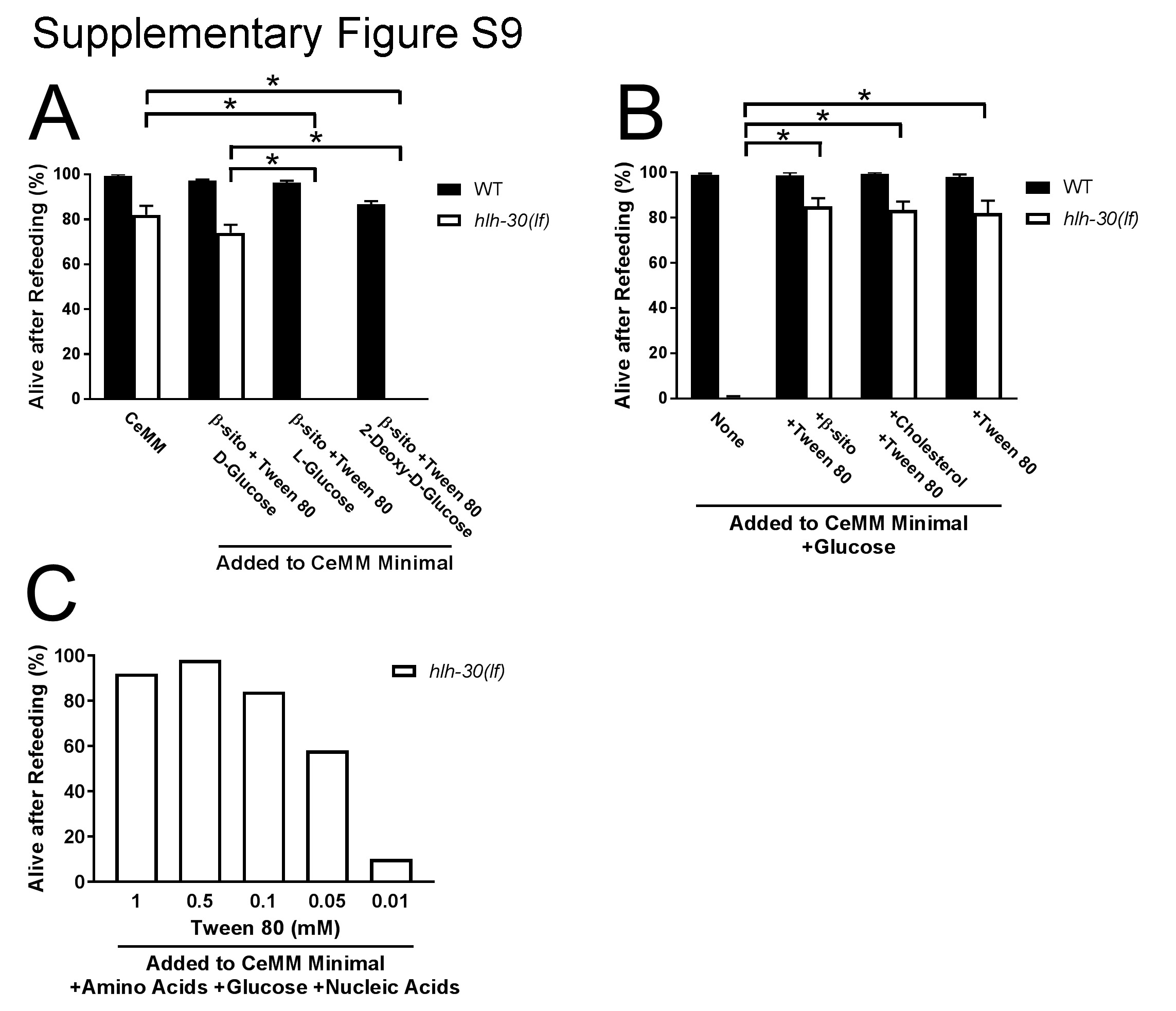

Supplement: S9 Fig — (A–C) Wild-type and hlh-30(lf) worms were analyzed after 33 hours of starvation and 15 hours of exposure to complete or modified formulations of CeMM for “Alive after Refeeding” as described in the legend for Fig 2A. Individual nutrients (shown below) were added to CeMM minimal solution (which lacks glucose, β-sitosterol in Tween 80, amino acids, and nucleic acids) in panel A, CeMM minimal solution containing glucose in panel B, or CeMM minimal solution containing amino acids, glucose, and nucleic acids in panel C. Bars indicate mean (± SEM). N = 3 biological replicates/group for panels A and B, and N = 1 replicate for panel C with approximately 50 worms/condition. *P < 0.05 by post hoc test after two-way ANOVA for A and B. Raw data are located in S2 Data. CeMM, C. elegans maintenance medium; hlh-30, basic helix–loop–helix transcription factor 30; hlh-30(lf), loss-of-function tm1978 mutation hlh-30; SEM, standard error of the mean. (TIF) [file pbio.3000245.s009.tif]

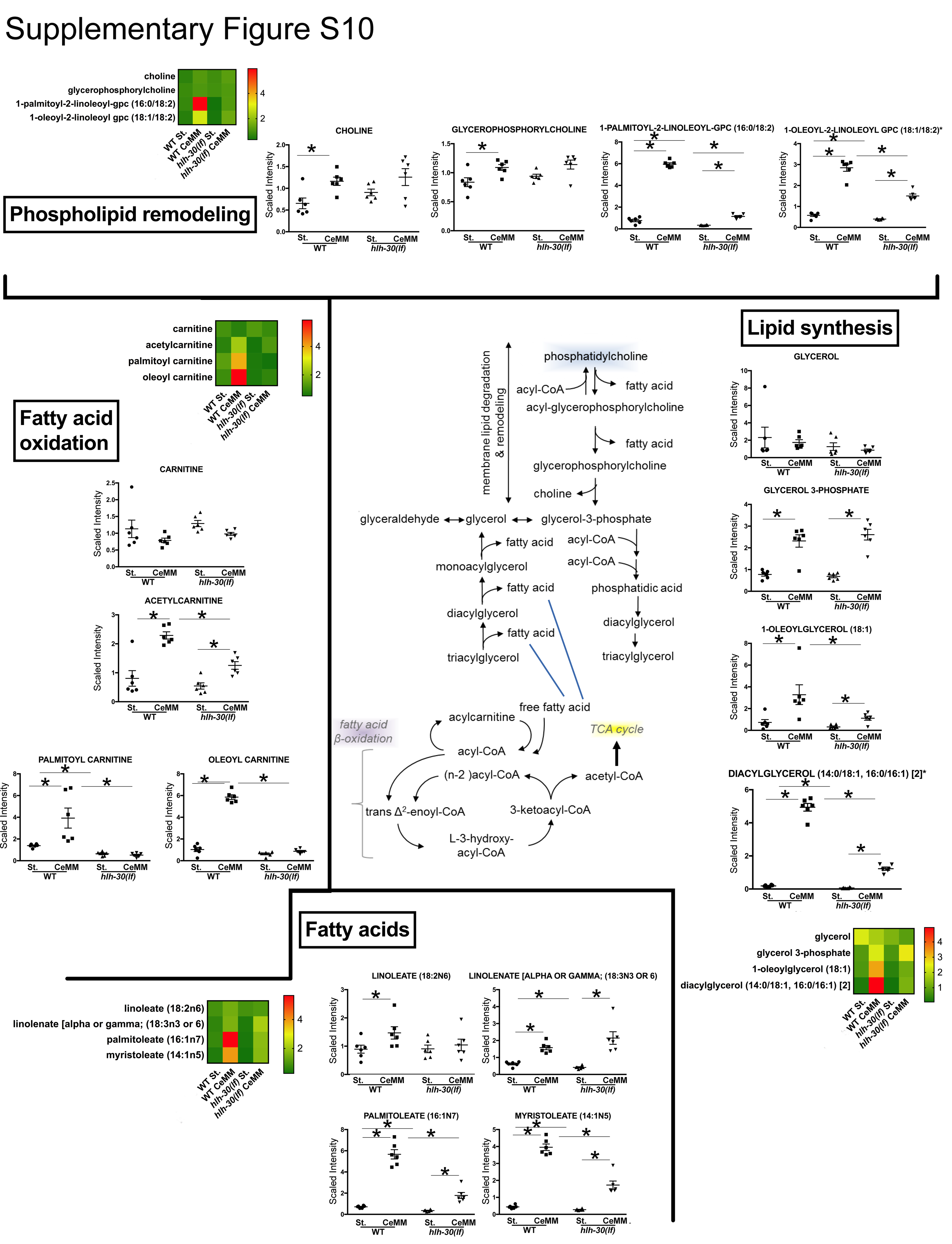

Supplement: S10 Fig — Depiction of significantly altered lipid metabolism pathways, along with significantly regulated candidate metabolites in hlh-30(lf) and wild-type worms subjected to 33 hours of starvation in M9 medium (labeled as St.) followed by 15 hours of incubation in CeMM, depicted in heat maps and graphs. Data on some metabolites presented in Fig 3 are also shown here to conform to the pathway depicted. Bars indicate mean ± SEM. N = 6 biological replicates/group. *P < 0.05 by post hoc test after two-way ANOVA. y-axis values are scaled intensity for each metabolite. See S4 Table for detail. Raw data are located in S2 Data. CeMM, C. elegans maintenance medium; hlh-30, basic helix–loop–helix transcription factor 30; hlh-30(lf), loss-of-function tm1978 mutation hlh-30; SEM, standard error of the mean. (TIF) [file pbio.3000245.s010.tif]

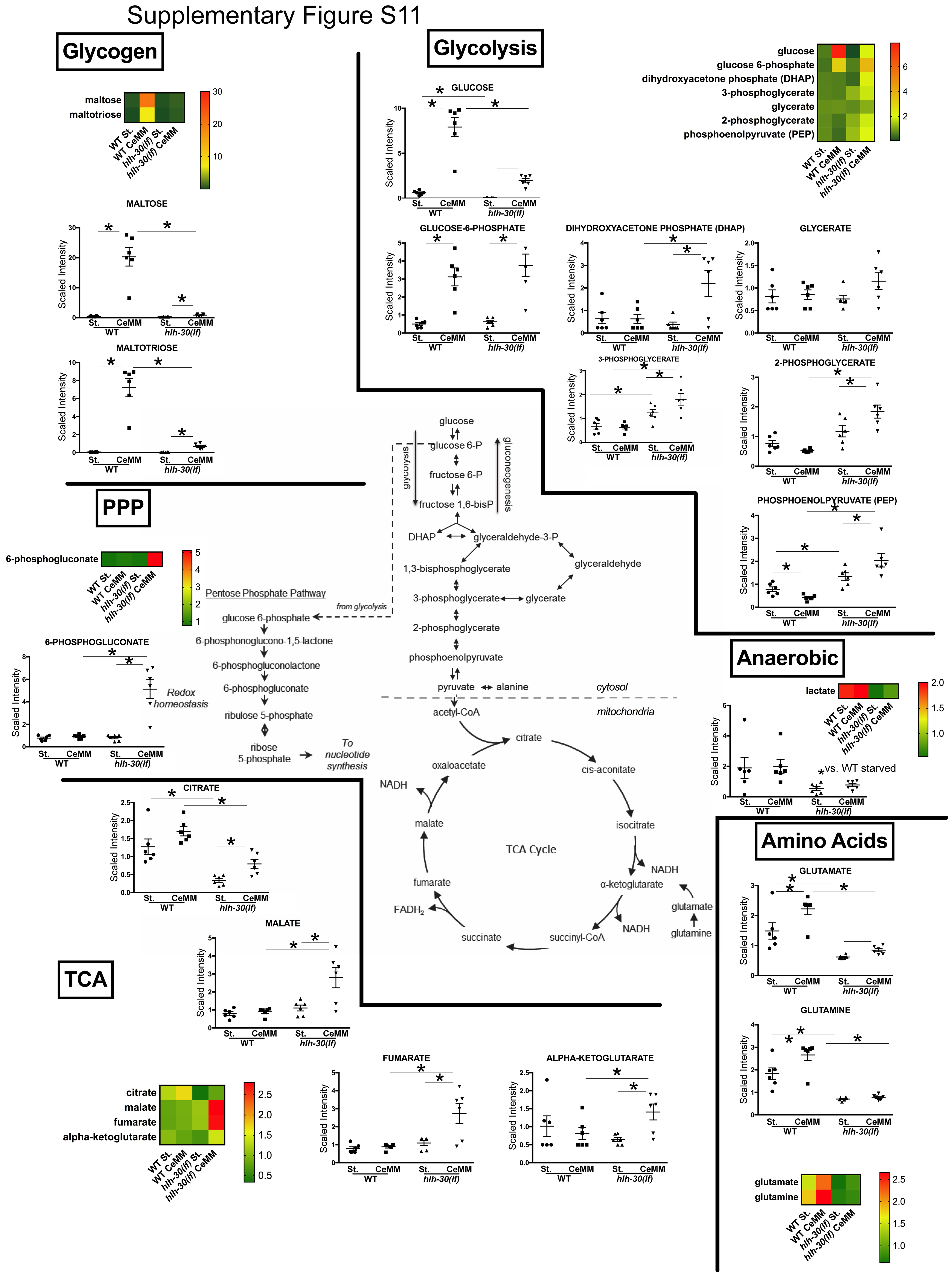

Supplement: S11 Fig — Depiction of various metabolic pathways that glucose is channeled into, along with significantly regulated metabolites in hlh-30(lf) and wild-type worms subjected to 33 hours of starvation in M9 medium (labeled as St.) followed by 15 hours of incubation in CeMM, depicted in heat maps and graphs. Data on some metabolites presented in Fig 3 are also shown here to conform to the pathway depicted. Bars indicate mean ± SEM. N = 6 biological replicates/group. *P < 0.05 by post hoc test after two-way ANOVA. y-axis values are scaled intensity for each metabolite. See S4 Table for detail. Raw data are located in S2 Data. CeMM, C. elegans maintenance medium; hlh-30, basic helix–loop–helix transcription factor 30; hlh-30(lf), loss-of-function tm1978 mutation hlh-30; SEM, standard error of the mean; TCA, tricarboxylic acid cycle. (TIF) [file pbio.3000245.s011.tif]

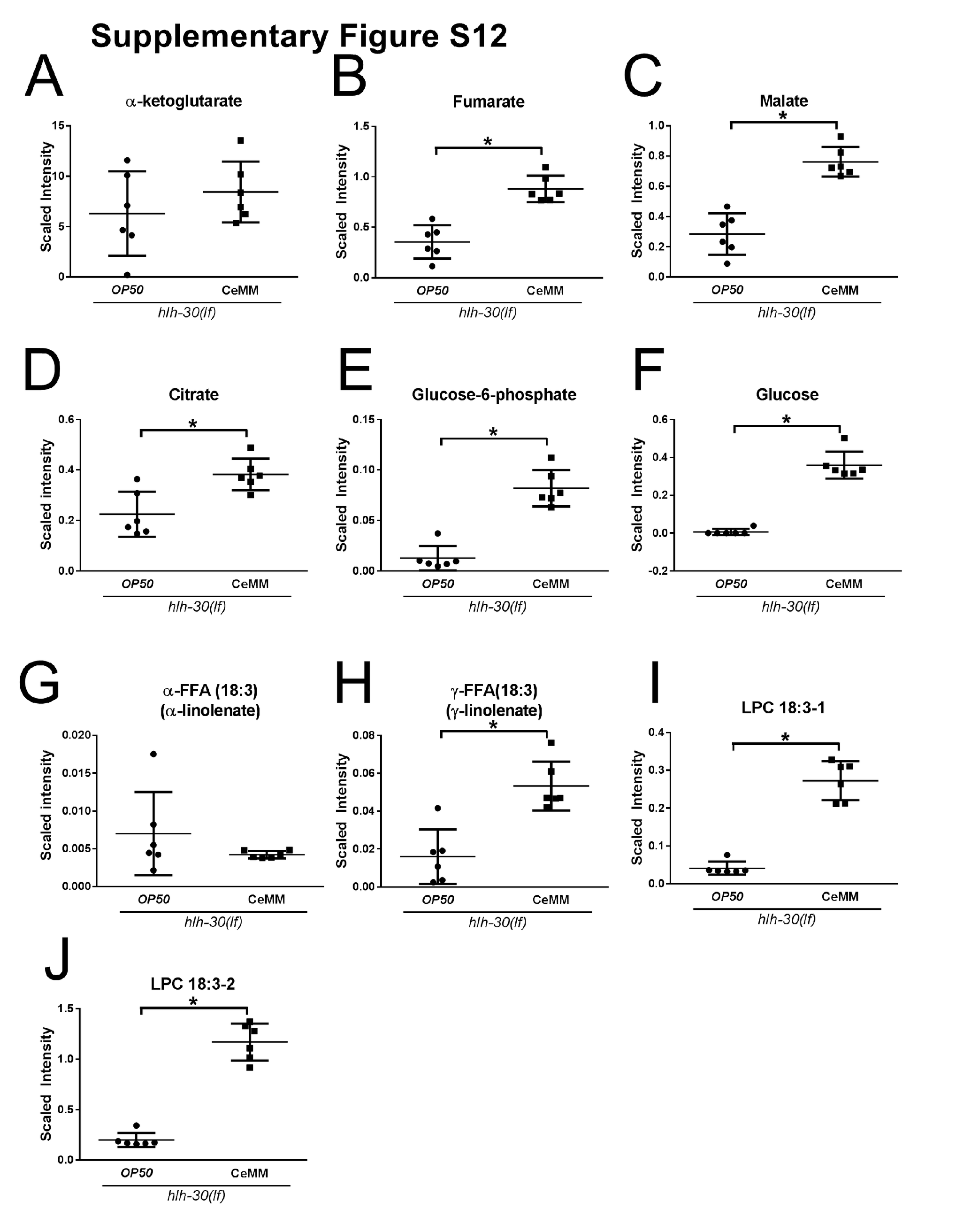

Supplement: S12 Fig — (A–J) hlh-30(lf) worms were subjected to metabolomics analyses after 33 hours of starvation and 15 hours of exposure to CeMM or E. coli OP50 to determine levels of α-ketoglutarate (A), fumarate (B), malate (C), citrate (D), glucose-6-phosphate (E), glucose (F), α-FFA (G), γ-FFA (H), LPC (18:3)-1 (I), and LPC (18:3)-2 (J). Data are presented as mean ± SEM. N = 4 biological replicates/group. *P < 0.05 by t test. Raw data are located in S2 Data. CeMM, C. elegans maintenance medium; FFA, linolenate; hlh-30, basic helix–loop–helix transcription factor 30; hlh-30(lf), loss-of-function tm1978 mutation hlh-30; LPC, lyso phosphatidyl choline; SEM, standard error of the mean; TCA, tricarboxylic acid cycle; α-FFA, α-linolenate; γ-FFA, γ-linolenate. (TIF) [file pbio.3000245.s012.tif]

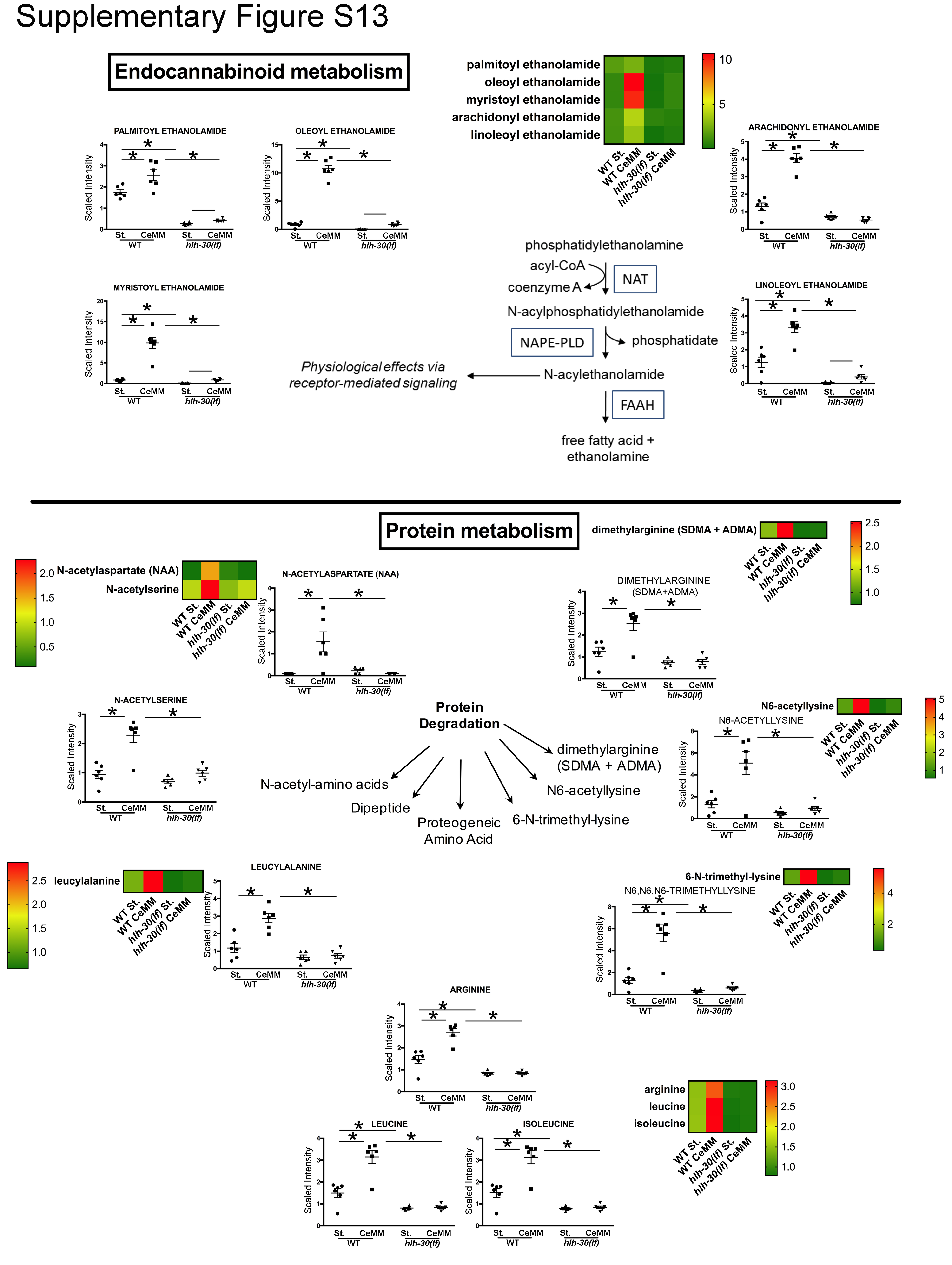

Supplement: S13 Fig — Depiction of significantly altered endocannabinoid and protein metabolism pathways, along with significantly regulated candidate metabolites in hlh-30(lf) and wild-type worms subjected to 33 hours of starvation in M9 medium (labeled as St.) followed by 15 hours of incubation in CeMM, depicted in heat maps and graphs. Bars indicate mean ± SEM. N = 6 biological replicates/group. *P < 0.05 by post hoc test after two-way ANOVA. y-axis values are scaled intensity for each metabolite. See S4 Table for detail. Raw data are located in S2 Data. CeMM, C. elegans maintenance medium; hlh-30, basic helix–loop–helix transcription factor 30; hlh-30(lf), loss-of-function tm1978 mutation hlh-30; SEM, standard error of the mean. (TIF) [file pbio.3000245.s013.tif]

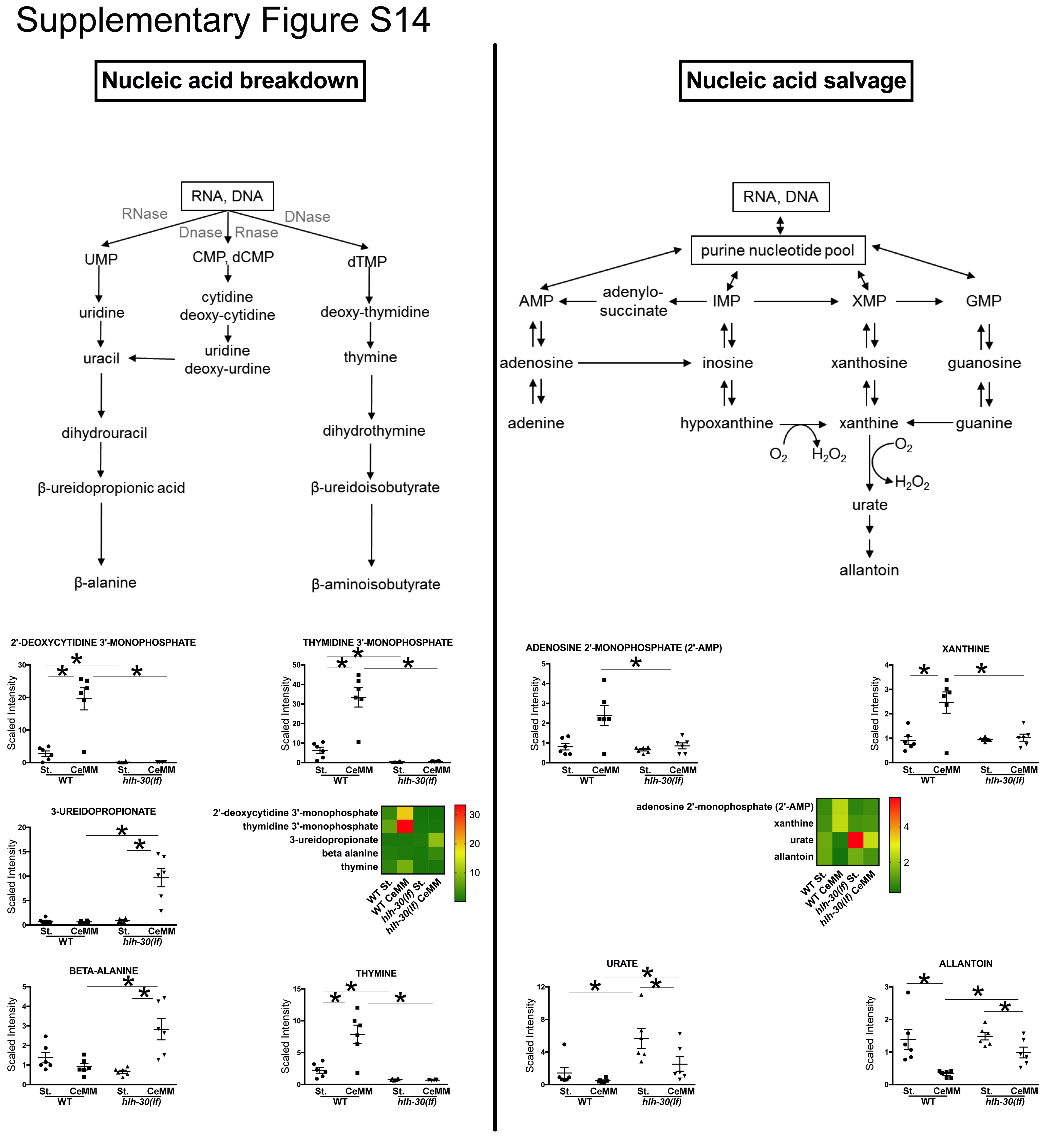

Supplement: S14 Fig — Depiction of significantly altered nucleic acid breakdown and salvage pathways, along with significantly regulated candidate metabolites in hlh-30(lf) and wild-type worms subjected to 33 hours of starvation in M9 medium (labeled as St.) followed by 15 hours of incubation in CeMM, depicted in heat maps and graphs. N = 6 biological replicates/group. *P < 0.05 by post hoc test after two-way ANOVA. Bars indicate mean ± SEM. y-axis values are scaled intensity for each metabolite. See S4 Table for detail. Raw data are located in S2 Data. CeMM, C. elegans maintenance medium; hlh-30, basic helix–loop–helix transcription factor 30; hlh-30(lf), loss-of-function tm1978 mutation hlh-30; SEM, standard error of the mean. (TIF) [file pbio.3000245.s014.tif]

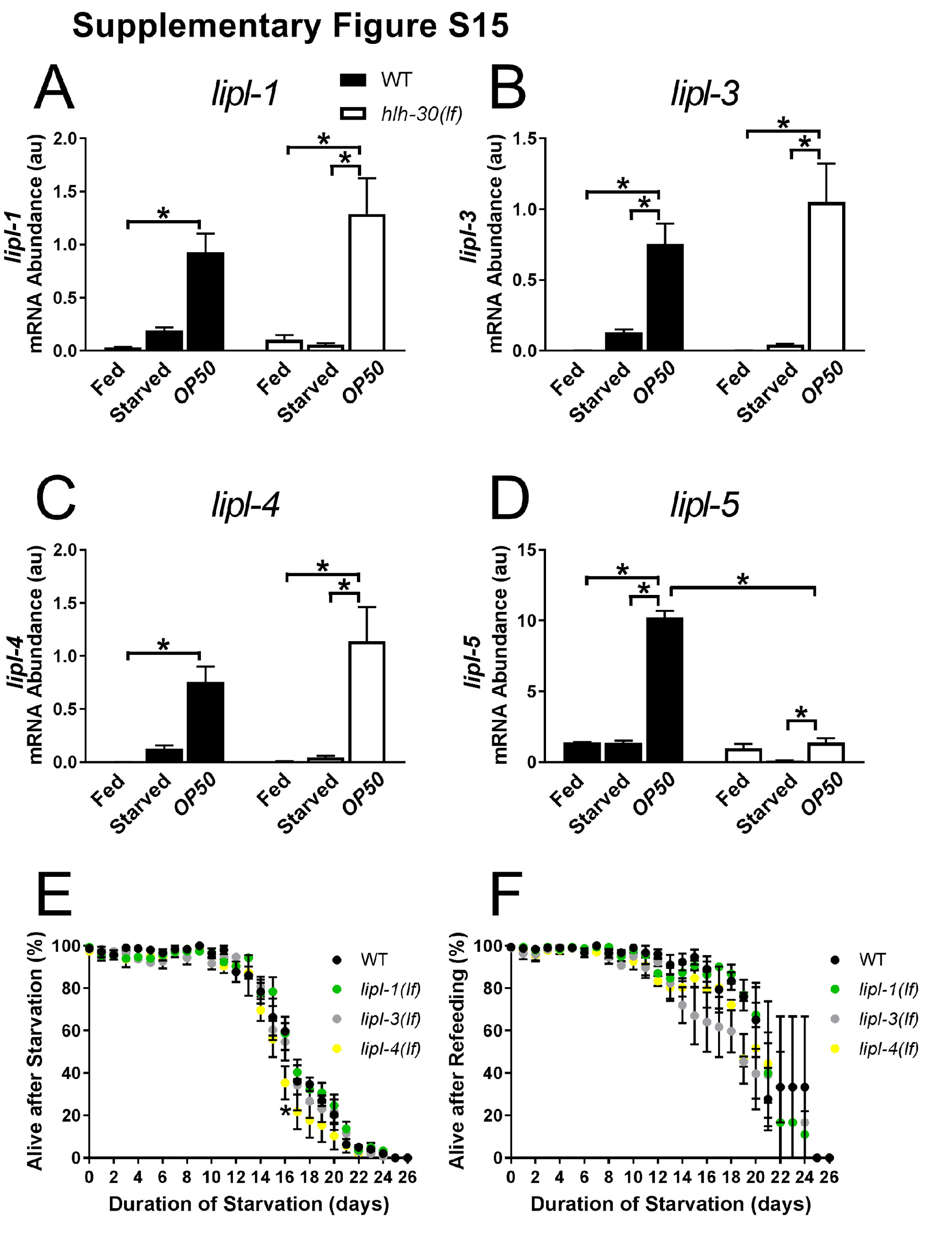

Supplement: S15 Fig — (A–D) mRNA abundance determined by qPCR in au for indicated genes in hlh-30(lf) and wild-type worms subjected to 33 hours of starvation or with 15 hours of refeeding with E. coli OP50. Fed worms are included as controls. Values were normalized to the control gene ama-1. Bars indicate mean ± SEM. N = 3 biological replicates/group. *P < 0.05 by post hoc test after two-way ANOVA. (E, F) Wild-type, lipl-1(lf), lipl-3(lf), and lipl-4(lf) worms were analyzed after variable periods of starvation for Alive after Starvation (E) and Alive after Refeeding (F) as described in the Fig 1 legend. Values are mean ± SEM. N = 3 biological replicates of approximately 25 worms/time point. *P < 0.05 versus wild type by post hoc test after two-way ANOVA. Raw data are located in S2 Data. ama-1, amanitin-binding subunit of RNA polymerase II; au, arbitrary unit; hlh-30, basic helix–loop–helix transcription factor 30; hlh-30(lf), loss-of-function tm1978 mutation hlh-30; lipl-1(lf), loss-of-function mutation in lysosomal lipase 1; SEM, standard error of the mean. (TIF) [file pbio.3000245.s015.tif]

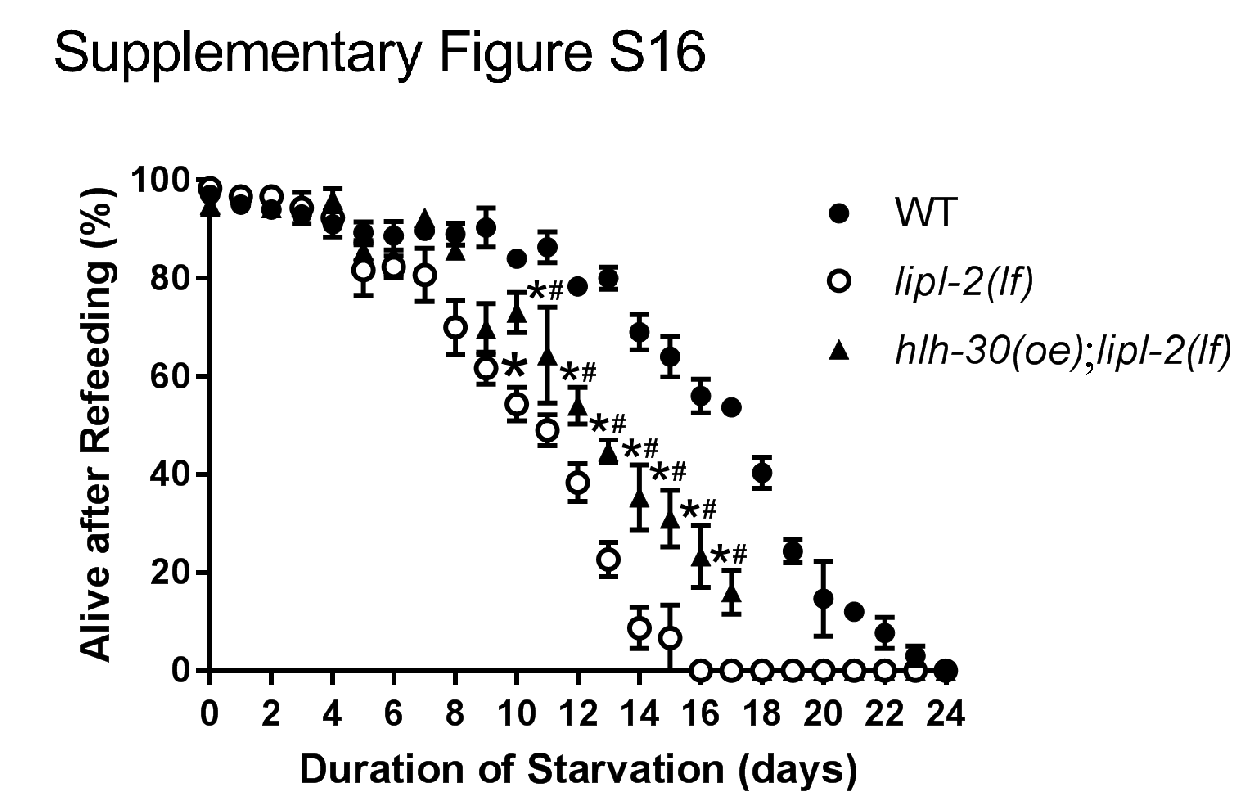

Supplement: S16 Fig — Wild-type, lipl-2(lf), and hlh-30(oe);lipl-2(lf) worms were analyzed after variable periods of starvation for Alive after Refeeding as described in the legend for Fig 1A. Values are mean ± SEM. N = 3 biological replicates of approximately 50 worms/time point. *P < 0.05 for hlh-30(oe);lipl-2(lf) versus wild type and #P < 0.05 for hlh-30(oe);lipl-2(lf) versus lipl-2(lf) by post hoc test after two-way ANOVA. Raw data are located in S2 Data. hlh-30, basic helix–loop–helix transcription factor 30; hlh-30(lf), loss-of-function tm1978 mutation hlh-30; hlh-30(oe), overexpressed HLH-30; lipl-2(lf), loss-of-function mutation in lysosomal lipase 2; SEM, standard error of the mean. (TIF) [file pbio.3000245.s016.tif]

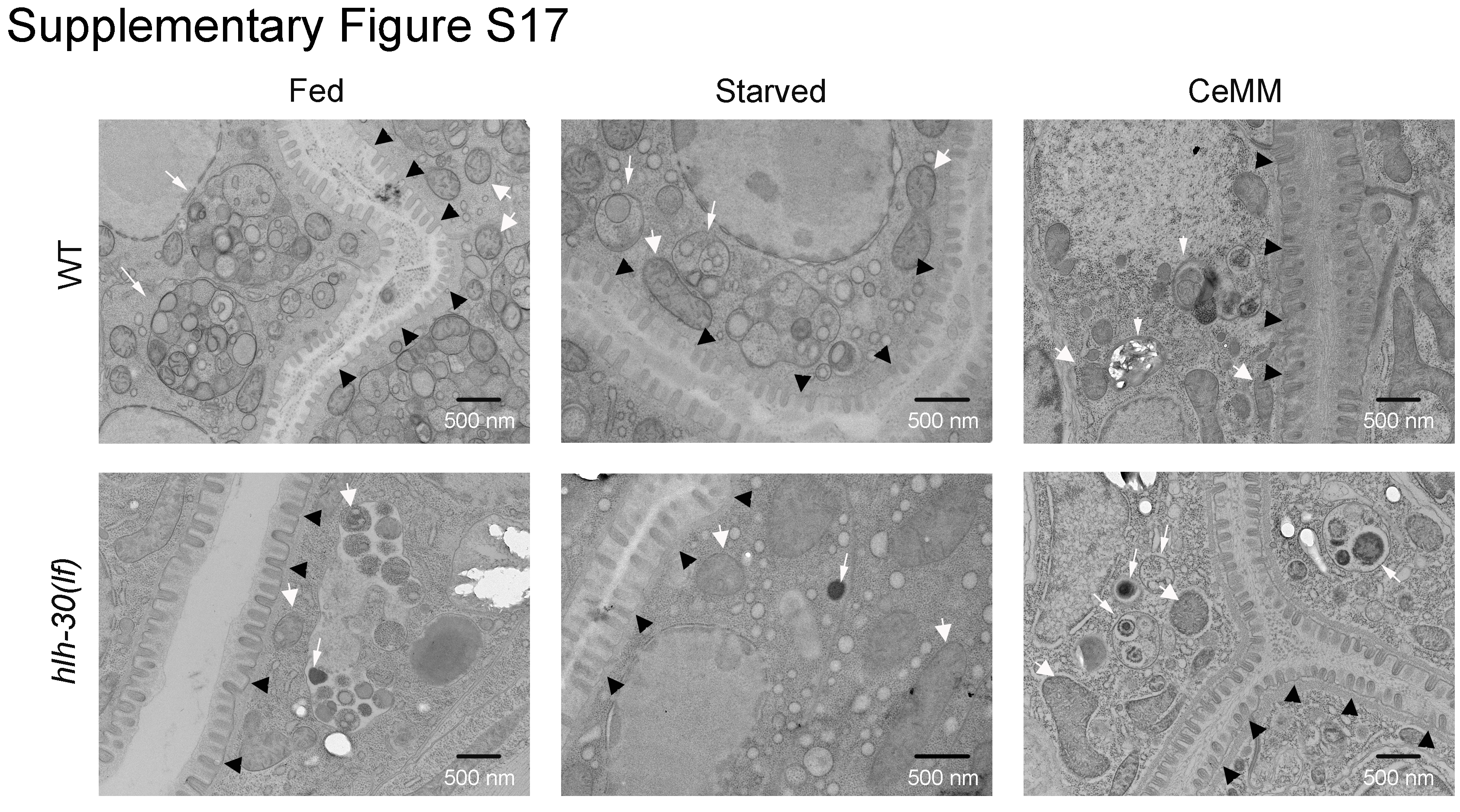

Supplement: S17 Fig — Transmission electron microscopic analyses of L1 stage wild-type and hlh-30(lf) worms analyzed in the fed state, after 33 hours of starvation, and after 15 hours of exposure to complete CeMM. Representative images are from one experiment; we performed two biological replicates/group. Black arrowheads point to the intestinal brush border, large white arrows point to mitochondria, and thin white arrows point to autophagic structures (autophagosomes and autolysosomes). Representative of N = 2 independent trials per group. Scale bars = 500 nm. CeMM, C. elegans maintenance medium; hlh-30, basic helix–loop–helix transcription factor 30; hlh-30(lf), loss-of-function tm1978 mutation hlh-30; L1, first larval stage. (TIF) [file pbio.3000245.s017.tif]

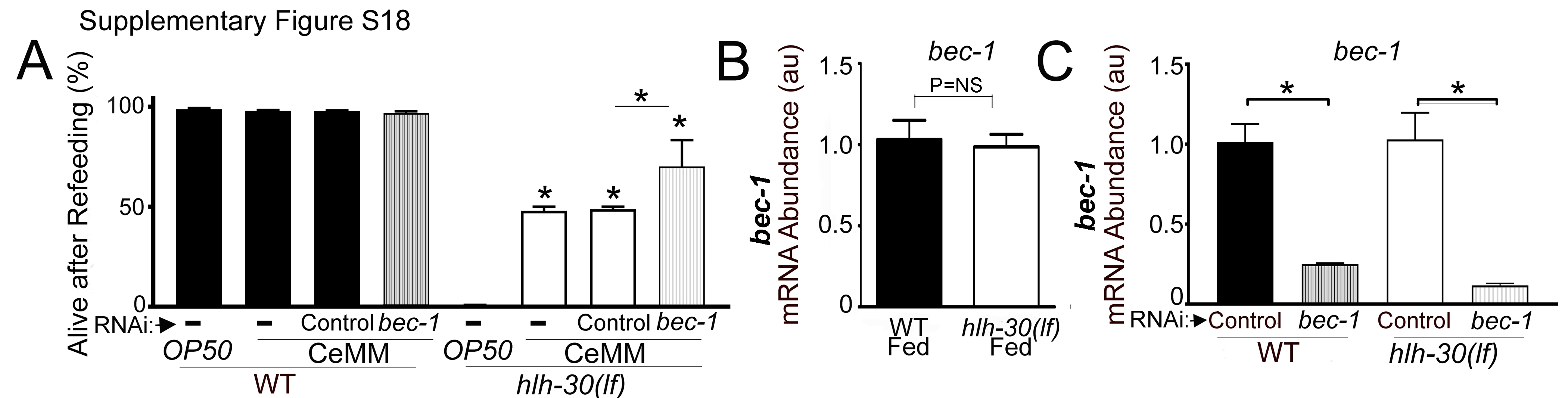

Supplement: S18 Fig — (A) Wild-type and hlh-30(lf) worms were analyzed after 33 hours of starvation and 0 or 15 hours of exposure to complete CeMM for “Alive after Refeeding” as described in the legend for Fig 2A. Worms were exposed to feeding RNAi during the L1 to L4 stages to reduce the level of bec-1 mRNA or L4440 as control and analyzed at the L4 stage. Bars indicate mean ± SEM. N = 3 biological replicates/group. *P < 0.05 by post hoc test after two-way ANOVA versus hlh-30(lf) with 0 hours of CeMM treatment (labeled OP50) or as indicated by horizontal line. (B) bec-1 mRNA abundance in au with values normalized to the control gene ama-1 determined by qPCR for fed wild-type and hlh-30(lf) worms. Bars indicate mean ± SEM. P = NS indicates that no statistically significant differences were noted by t test. (C) bec-1 mRNA abundance was analyzed in wild-type and hlh-30(lf) L4 stage worms 72 hours after starvation. The control RNAi (L4440) value was set equal to 1.0 for both genotypes, and bec-1 values were normalized to the expression of ama-1. Bars indicate mean ± SEM. N = 3 biological replicates/group. *P < 0.05 by post hoc test after one-way ANOVA. Raw data are located in S2 Data. ama-1, amanitin-binding subunit of RNA polymerase II; au, arbitrary unit; bec-1, C. elegans ortholog of human BECN1; CeMM, C. elegans maintenance medium; hlh-30, basic helix–loop–helix transcription factor 30; hlh-30(lf), loss-of-function tm1978 mutation hlh-30; L1, first larval stage; L4, fourth larval stage; NS, not significant; qPCR, quantitative PCR; RNAi, RNA interference; SEM, standard error of the mean. (TIF) [file pbio.3000245.s018.tif]

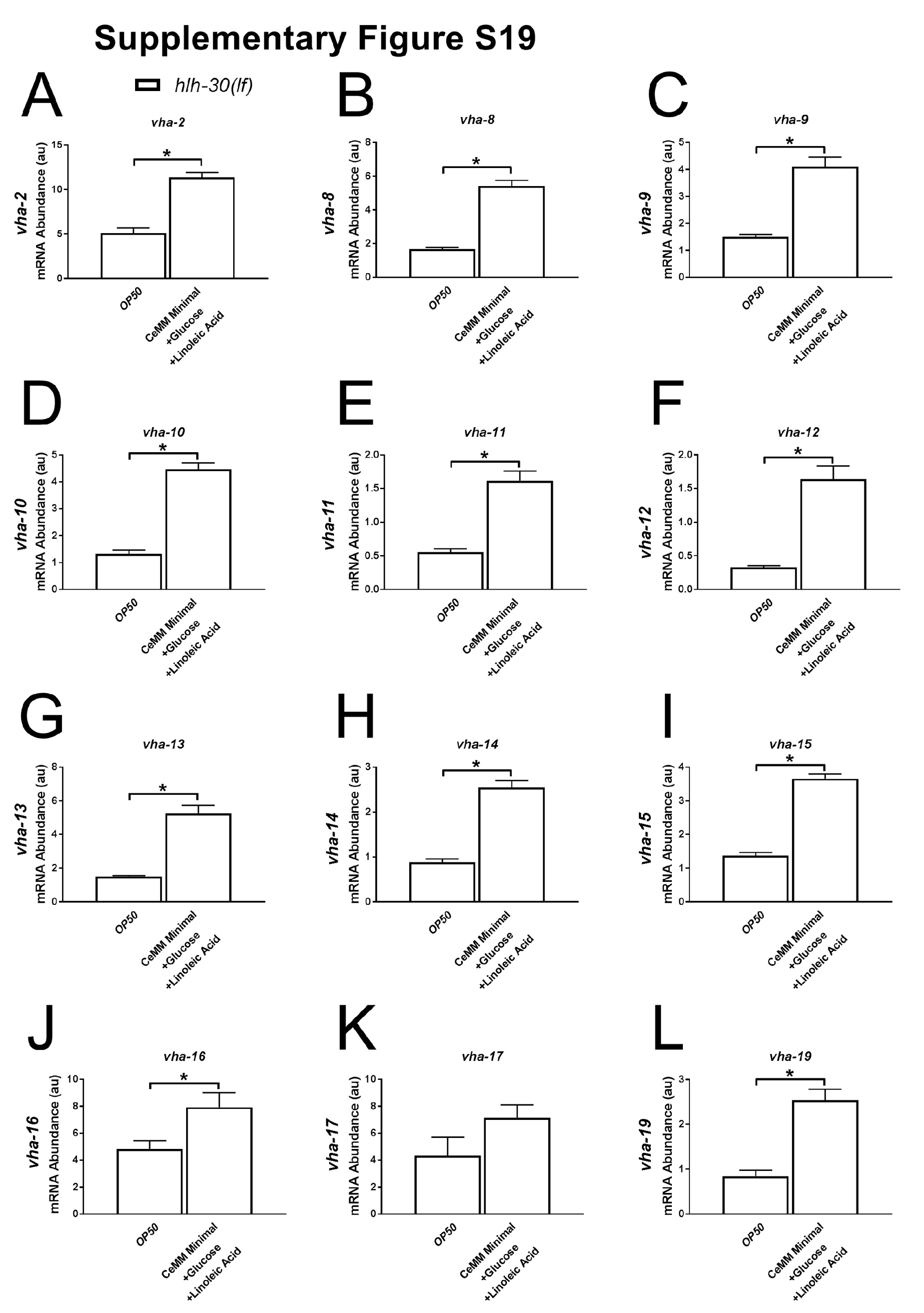

Supplement: S19 Fig — (A–L) mRNA abundance in au with values normalized to the control gene ama-1 determined by qPCR for the indicated genes. hlh-30(lf) L1 worms were starved for 33 hours and then transferred to NGM dishes with E. coli OP50 for 15 hours or fed CeMM minimal supplemented with glucose and linoleic acid (as described in Fig 2E) for 15 hours. Bars indicate mean ± SEM. N = 4–8 biological replicates/group. *P < 0.05 by t test. Raw data are located in S2 Data. ama-1, amanitin-binding subunit of RNA polymerase II; au, arbitrary unit CeMM, C. elegans maintenance medium; hlh-30, basic helix–loop–helix transcription factor 30; hlh-30(lf), loss-of-function tm1978 mutation hlh-30; L1, first larval stage; NGM, nematode growth medium; qPCR, quantitative PCR; SEM, standard error of the mean; vha, vacuolar H+-ATPase. (TIF) [file pbio.3000245.s019.tif]

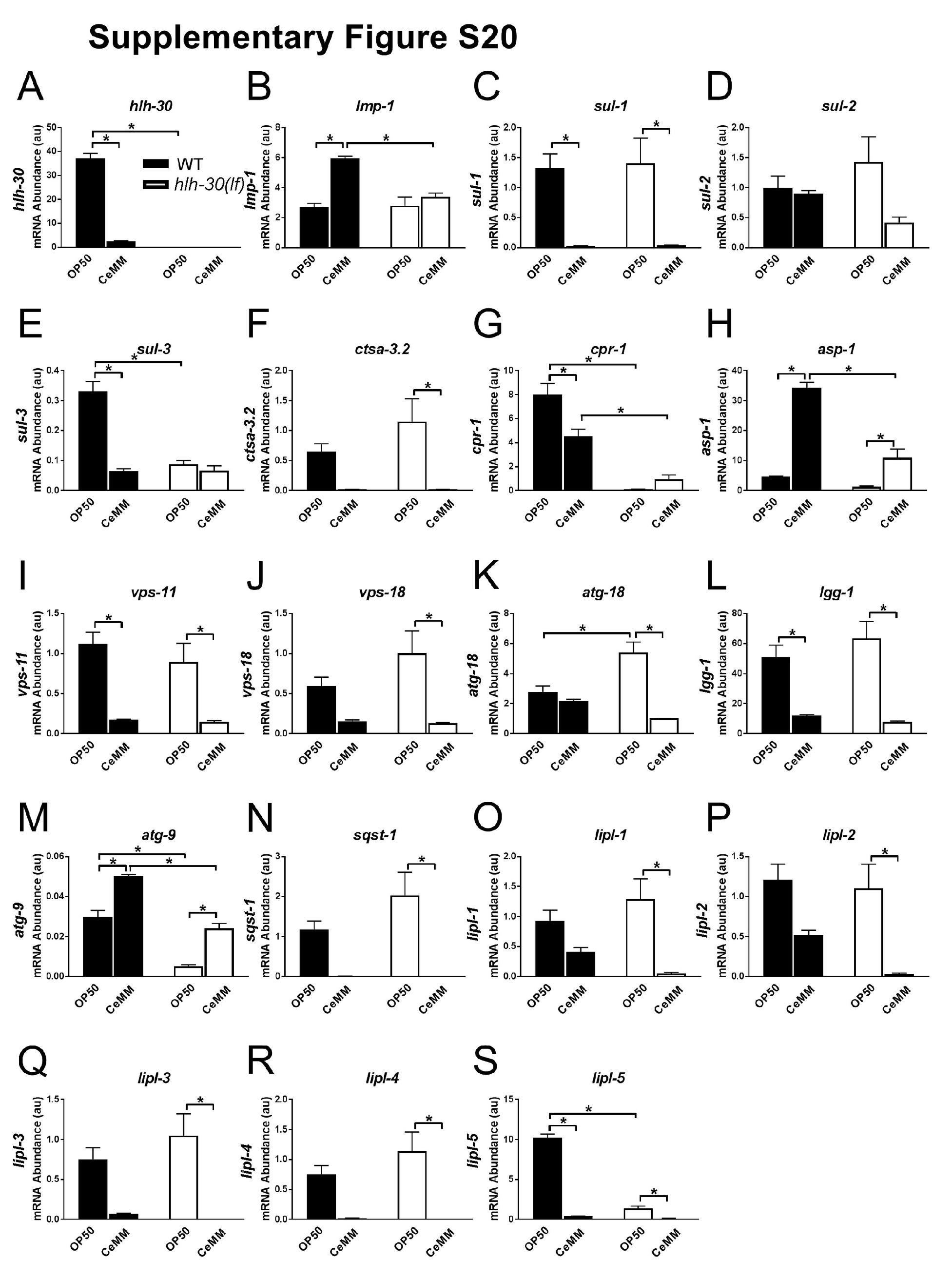

Supplement: S20 Fig — (A–S) mRNA abundance in au with values normalized to the control gene ama-1 determined by qPCR for the indicated genes. Wild-type and hlh-30(lf) L1 worms were starved for 33 hours and transferred to NGM dishes with E. coli OP50 for 15 hours or fed CeMM for 15 hours. Bars indicate mean ± SEM. N = 3 biological replicates/group. *P < 0.05 by post hoc test after two-way ANOVA. Raw data are located in S2 Data. ama-1, amanitin-binding subunit of RNA polymerase II; au, arbitrary unit; CeMM, C. elegans maintenance medium; hlh-30, basic helix–loop–helix transcription factor 30; hlh-30(lf), loss-of-function tm1978 mutation hlh-30; L1, first larval stage; NGM, nematode growth medium; qPCR, quantitative PCR; SEM, standard error of the mean. (TIF) [file pbio.3000245.s020.tif]

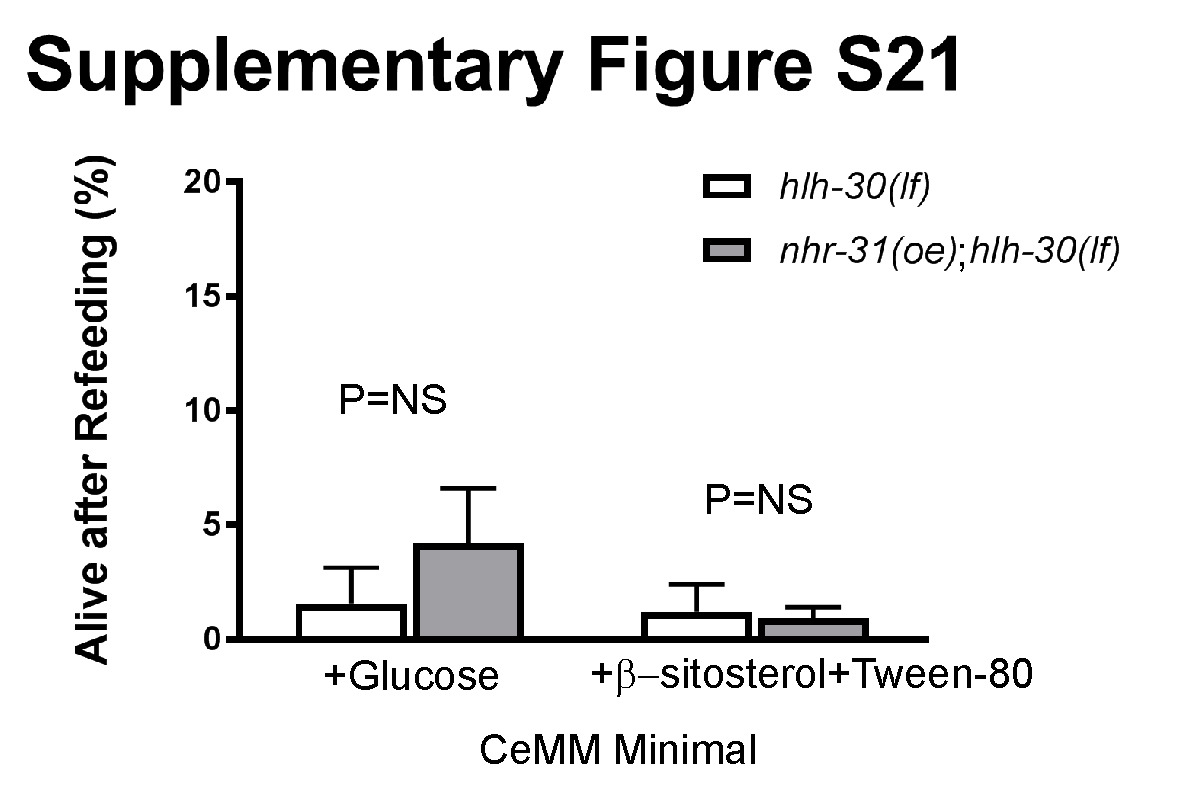

Supplement: S21 Fig — nhr-31 overexpressing hlh-30(lf) worms (nhr-31(oe);hlh-30(lf)) have approximately 17-fold higher levels of nhr-31 transcripts compared to the wild type. Animals were subjected to 33 hours of starvation followed by refeeding with CeMM minimal (see Fig 2D) supplemented with glucose or β-sitosterol with Tween 80. “Alive after Refeeding” was scored as described in the legend for Fig 2A. Bars indicate mean ± SEM. N = 3 biological replicates/group. No significant differences were observed between groups by two-way ANOVA. Raw data are located in S2 Data. CeMM, C. elegans maintenance medium; hlh-30, basic helix–loop–helix transcription factor 30; hlh-30(lf), loss-of-function tm1978 mutation hlh-30; nhr-31, nuclear hormone receptor 31; nhr-31(oe), overexpressed nhr-31; SEM, standard error of the mean. (TIF) [file pbio.3000245.s021.tif]

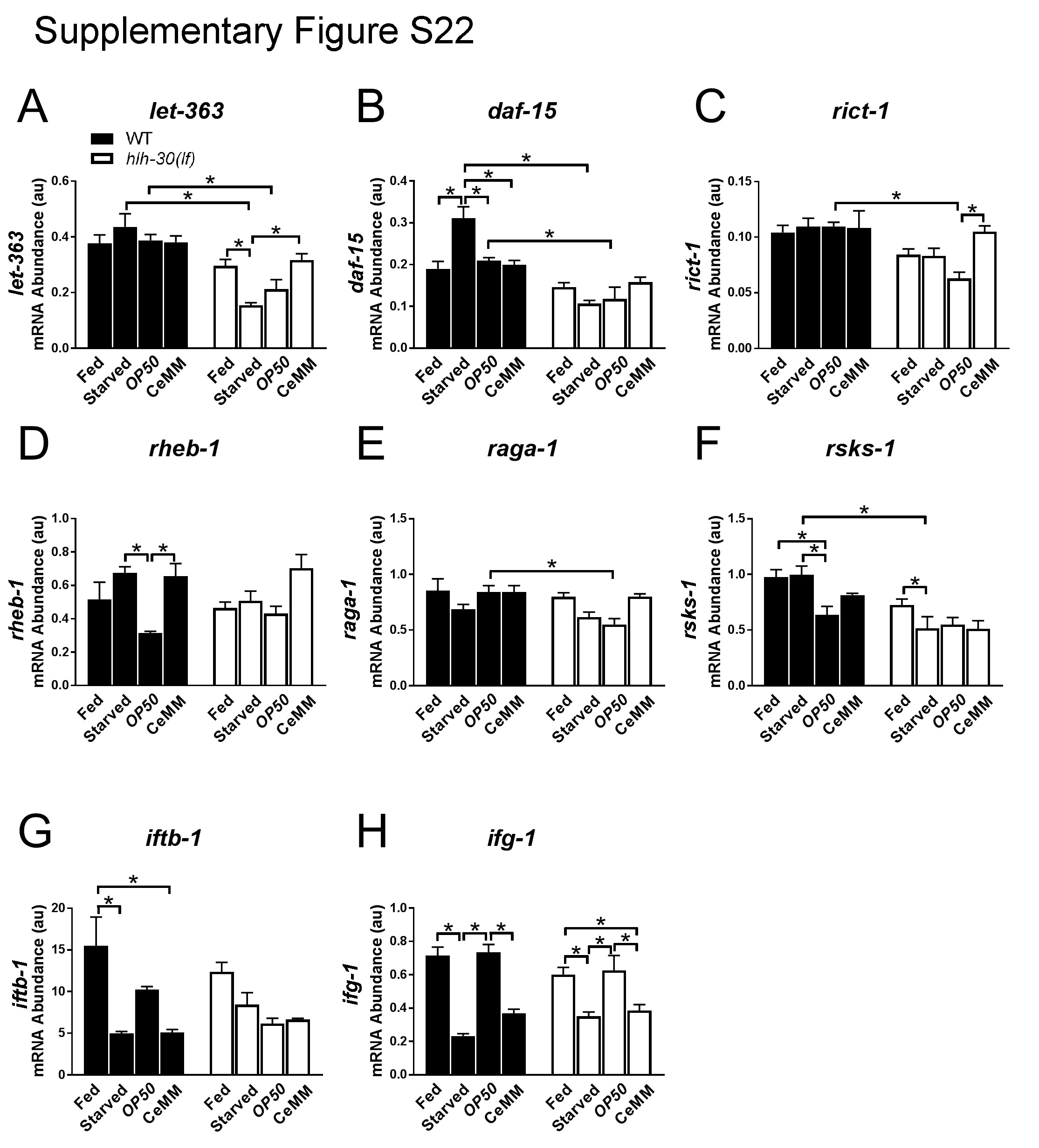

Supplement: S22 Fig — (A–H) mRNA abundance determined by qPCR in au with values normalized to the control gene ama-1 for genes (as named) in L1 stage wild-type and hlh-30(lf) animals in the fed state (fed), after starvation for 33 hours (starved), and after starvation for 33 hours followed by refeeding on E. coli OP50 (OP50) or CeMM (CeMM) for 15 hours. Bars indicate mean ± SEM. N = 6 biological replicates/group. *P < 0.05 by post hoc test after one-way ANOVA. Raw data are located in S2 Data. ama-1, amanitin-binding subunit of RNA polymerase II; au, arbitrary unit; CeMM, C. elegans maintenance medium; hlh-30, basic helix–loop–helix transcription factor 30; hlh-30(lf), loss-of-function tm1978 mutation hlh-30; L1, first larval stage; qPCR, quantitative PCR; SEM, standard error of the mean; TOR, target of rapamycin. (TIF) [file pbio.3000245.s022.tif]

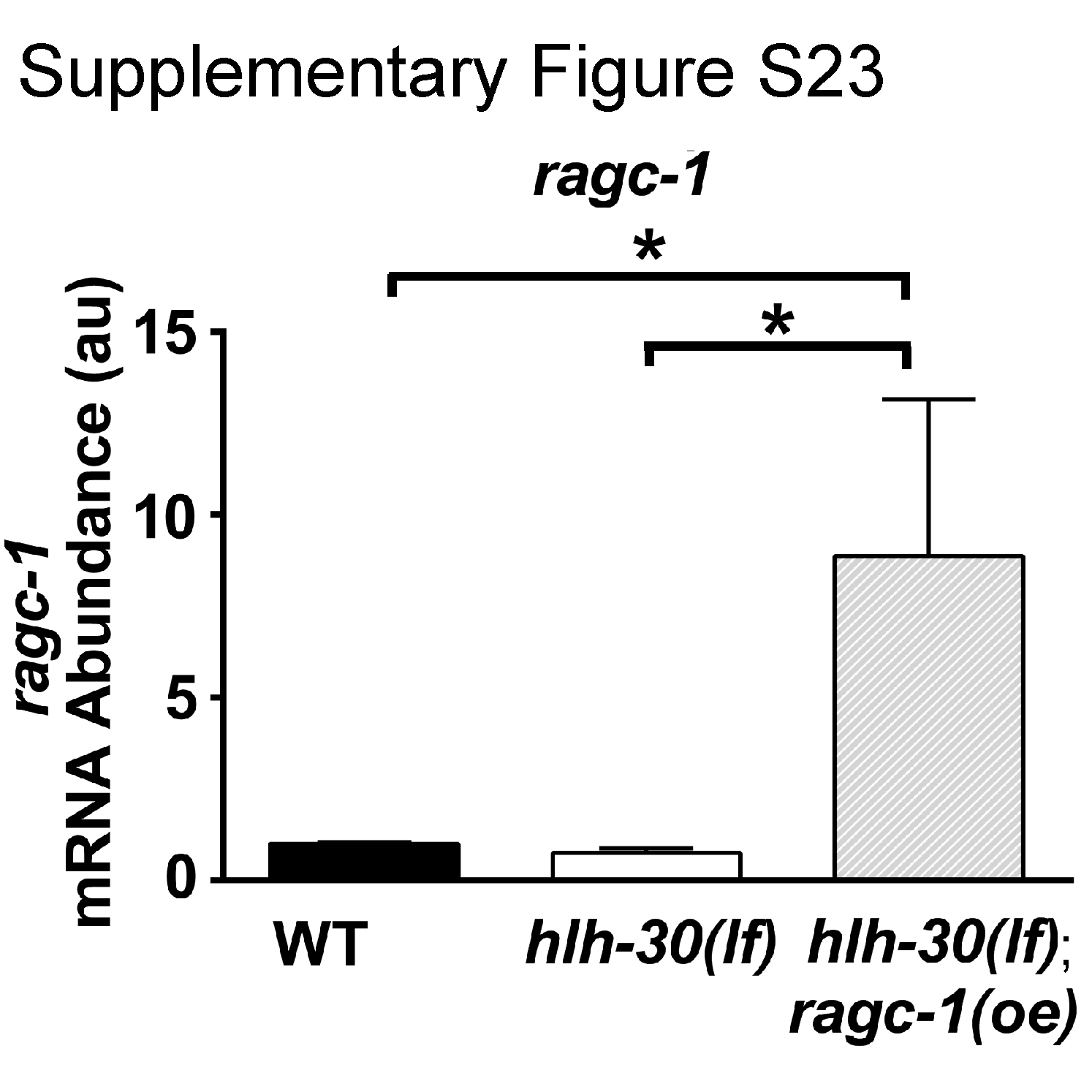

Supplement: S23 Fig — ragc-1 mRNA abundance in au with values normalized to the control gene ama-1 was analyzed in fed L4 stage worms in the following groups: wild-type, hlh-30(lf), and hlh-30(lf);ragc-1(oe) (hlh-30(lf);amEx324 worms that overexpress ragc-1 from an extrachromosomal array utilizing its endogenous promoter). Bars indicate mean ± SEM. N = 3–6 biological replicates/group. *P < 0.05 by post hoc test after one-way ANOVA. ama-1, amanitin-binding subunit of RNA polymerase II; au, arbitrary unit; hlh-30, basic helix–loop–helix transcription factor 30; hlh-30(lf), loss-of-function tm1978 mutation hlh-30; L4, fourth larval stage; ragc-1, the ortholog for mammalian RagC/D GTPases; ragc-1(oe), overexpressed ragc-1; SEM, standard error of the mean. (TIF) [file pbio.3000245.s023.tif]

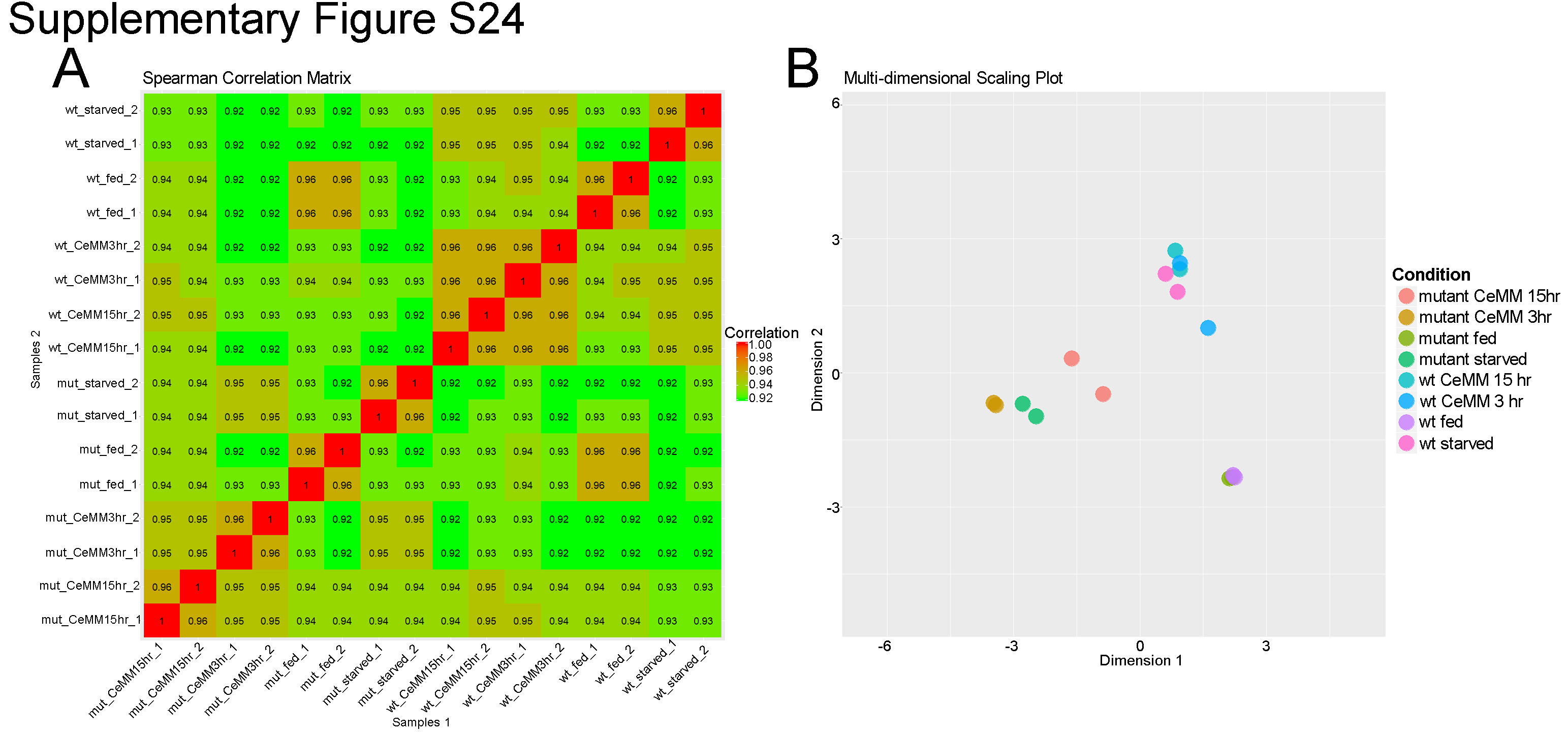

Supplement: S24 Fig — (A, B) Spearman correlation matrix (A) and multidimensional scaling plot (B) depicting various RNA samples from wild-type and hlh-30(lf) L1 worms subjected to 33 hours of starvation, 33 hours of starvation followed by 3 or 15 hours of CeMM exposure (as in Fig 2B), or studied in the fed state and subjected to RNAseq. CeMM, C. elegans maintenance medium; hlh-30, basic helix–loop–helix transcription factor 30; hlh-30(lf), loss-of-function tm1978 mutation hlh-30; L1, first larval stage; RNAseq, RNA sequencing. (TIF) [file pbio.3000245.s024.tif]
