# Supplementary material for: Simple nutrients bypass the requirement for HLH-30 in coupling lysosomal nutrient sensing to survival
Source: PLoS Biol. 2019 May 14;17(5):e3000245. doi: 10.1371/journal.pbio.3000245 (PMC6516633; doi:10.1371/journal.pbio.3000245)
Supplement: S1 Table — (PDF) [file pbio.3000245.s031.pdf]

Table S1: Description of mutant alleles used in this study:

Genotype(Allele)	Abbreviated Name	Genetic Lesion
hlh-30(tm1978)	*hlh-30(lf)*	710bp deletion; eliminates two exons shared by all isoforms
lipl-1(tm1954)	*lipl-1(lf)*	1033bp deletion and 8bp insertion; eliminates 418bp of exon three
lipl-2(tm4324)	*lipl-2(lf)*	510bp deletion; eliminates exon two and three; and 240bp of exon four
lipl-3(tm4498)	*lipl-3(lf)*	192bp deletion; eliminates exon two
lipl-4(tm4417)	*lipl-4(lf)*	452bp deletion and 6bp insertion; eliminates 412bp in exon four of isoform a and 412bp in exon two of isoform b
ragc-1(tm1974)	*ragc-1(lf)*	409bp deletion; eliminates 44bp in exon one, all of exon two and 143bp in exon three of isoform a
