# Supplementary material for: Simple nutrients bypass the requirement for HLH-30 in coupling lysosomal nutrient sensing to survival
Source: PLoS Biol. 2019 May 14;17(5):e3000245. doi: 10.1371/journal.pbio.3000245 (PMC6516633; doi:10.1371/journal.pbio.3000245)
Supplement: S3 Table — (PDF) [file pbio.3000245.s033.pdf]

Table S3: List of *C. elegans* strains used in this study:

Strain	Abbreviated Name	Details
N2	WT	N2 Bristol or wild-type strain
PD4666	*phlh-8::gfp*	*ayls6[hlh::GFP fusion + dpy-20(+)]*
WU1598	*hlh-30(oe)*	*hlh-30p::hlh-30::RFP* in *hlh-30(lf)* with *rol-6(su1006)* co-injected as marker
WU1617	*hlh-30mNLS(oe)*	*hlh-30p::hlh-30mNLS::RFP* in *hlh-30(lf)* with *rol-6(su1006)* co-injected as marker
WU1761	*ragc-1(oe) hlh-30(lf)*	*ragc-1p::ragc-1* in *hlh-30(lf)* with *sur5::GFP* co-injected as marker
WU1838	*nhr-31(oe) hlh-30(lf)*	*nhr-31p::FLAG3X::nhr-31* in *hlh-30(lf)* with *sur5::GFP* co-injected as marker
WU1874	*ragc-1(oe)*	*ragc-1p::ragc-1* in N2 with *sur5::GFP* co-injected as marker
WU1875	*hlh-30(oe) lipl-2(lf)*	*hlh-30p::hlh-30::RFP* in *lipl-2(lf)* with *rol-6(su1006)* co-injected as marker
WU1876	*phlh-8::gfp*;*hlh-30(lf)*	*ayls6 [hlh::GFP fusion + dpy-20(+)]; hlh-30(tm1978)*
