# Supplementary material for: Simple nutrients bypass the requirement for HLH-30 in coupling lysosomal nutrient sensing to survival
Source: PLoS Biol. 2019 May 14;17(5):e3000245. doi: 10.1371/journal.pbio.3000245 (PMC6516633; doi:10.1371/journal.pbio.3000245)
Supplement: S6 Table — qPCR, quantitative PCR. (PDF) [file pbio.3000245.s036.pdf]

Table S6: Primer description for quantitative PCR analysis of indicated genes.

Worm genes	Forward primer (5'-3')	Reverse primer (5'-3')
ama-1	ATCGGAGCAGCCAGGAACTT	GACTGTATGATGGTGAAGCTGG
asp-1	GGTGCCAACGCACAAGACCG	AGCCTGGGTCTTGCATGGGG
atg-9	GGCCGCCATCCACTCATCGG	TTGACGTCGTGCCGCCGTAG
atg-18	AAATGGACATCGGCTCTTTG	TGATAGCATCGAACCATCCA
cpr-1	ACCTTGGCCTTTCCGGCGAC	CGCCAAGGACAAGCACTTCGGA
ctsa-1	TTCTCCTCGAGGCGCGGGAT	TCCAACGCCAATTGGGGACTC
glo-1	TGGGATATTTCAGGCCAAGA	TCTTCCAGCGTAAAGCACCT
glo-3	GTGTTGGTGTTTGCCAATGA	ACGGTCGTGAGATCAGTGTG
hlh-30	ACGAGTTCGATCGACATTGAG	CGGTCGCGGTAATAGTCCT
lgg-1	ACCCAGACCGTATTCCAGTG	ACGAAGTTGGATGCGTTTTC
lipl-1	GTTTGTGACGATGTGATGTTCC	AAGTTCCTGCGGGTGTATG
lipl-2	AGAGGCTGAACTCTGTGATAATG	GCTGGATCCTGTGAACTGTAA
lipl-3	CCGCTATCAAGGGAACTCAAA	TTCAAGCGAGTCAGCAAGTAA
lipl-4	GTATTGCGGATGTTCCCATCTA	CTTCCCTGAACGACTTGAGAAT
lipl-5	GGGAACCAAGACGAACAAGA	GCATCACTCCAGTAGAGGTAAAT
lmp-1	ATCCGCCACCGCTTCGCATT	TCGAGCTCCCACTCTTTGGCG
rps-23	AAGGCTCACATTGGAACTCG	AGGCTGCTTAGCTTCGACAC
sqst-1	TGGCTGCTGCATCATCCGCT	TCAATCGTGCCGAGACCGGG
sul-1	CAGGATGGGATGAGTGGCACG	GGGTCTTCTGGTCCATGCGGC
sul-2	ATGGCAGCAGAAGGCACCCG	GCCATTTTCCAACCATGCCAGTTGC
sul-3	ACATGGAGCCTGCCGGTGTG	TGGTACGCTTTTTCCGCTGCCA
vha-2	TGCTCAATGGGTGTGATGAG	AACCATAGCCACGACCAATC
vha-8	CTGCTGGAGGAGTTGAACTTT	ACTTGTGGGACAATCTGGTTAG
vha-9	CGAAGATACCGTTGTTGGATTTC	TTGGACAGTGGTCTGCTTATC
vha-10	GAGCAAGATTCGTCGTGATACT	GTTGAAGGAGACGGACAATGA

vha-11	GCAAATGATGCGTGGGATAAG	AGGTTCCGACCTTCAAATCAG
vha-12	CTGCGAGTTCACTGGAGATATT	GTCCCTTGTCAATTGGCTTTC
vha-13	TTGGAACCGTCGATGAGAAC	GGGTGTCCTCAACTGTGTATT
vha-14	GATGCGTTGAATCTTCGTTTCC	CAGCCTCTTTCATAACCTCTCC
vha-15	CGAGGTTCGTTCCGGACGTCTT	CCTCGGCAGTCAGGAGACGC
vha-16	AGGCGCTGACTCGCGGACTT	TGGTCTCTGGTGAAGAGTTCCGGTG
vha-17	TCGGTTTCCGCCTTCTGGGC	ACATCCAGCAGCAAACAGCAGTCA
vha-19	AATCCCGCGTGGAACTTT	GGAGCTCATCTGGTTGACTATG
vps-11	TCCGCTTGTCGTCCTGGAGC	TCACACGCCGAGCACTTGGT
vps-18	CGAGCCGGCGCCAGTTGTAA	TCCATCCGGCGAAAGCCACG
raga-1	CATGGAGGACAAGCTGAAGAA	GAGAACAGCATGCGCAATAAC
ragc-1	GCCGAGAATGGCTCAAACTA	CTTGTTGACTTGACGGAGGAA
nhr-31	TGTCGAAGATTGGGAGTATTGG	CCTCGCATCATTGGAACTCT
act-1	GCTGGACGTGATCTTACTGATT	GTAGCAGAGCTTCTCCTTGATG
ifg-1	ATTGGATAGGAACCGAGGAATG	CATCGAAGAGAGCGAGATGTT
iftb-1	TTGAAAGTGTCCTCCGGAAAT	TGGAGGAAGAAGAGACGAGTAT
rict-1	GAGACCGGCAAGGAAGATTAC	CTCGTCGAACTAGAGCACTTTC
rsks-1	GGCAAGGGAGGATATGGAAAG	GGCTTTCGTGTGAGCTGTAT
rheb-1	GACAATGGGATGCGAAGTTTG	CGCTCCGTTGGTGATAGATT
let-363	GAGCTGGTTGATCGTTGTCTT	GGCGAGTCGACGAATTTGAT
daf-15	CAATCTAGACAACCGCAAGGAA	CGCCGAGAAGTTGAAGGAAATA
